# Variability in foraging ranges of snow petrels and implications for breeding distribution and use of stomach-oil deposits as proxies for paleoclimate

**DOI:** 10.1186/s40462-025-00609-7

**Published:** 2025-11-20

**Authors:** Ewan D. Wakefield, Erin L. McClymont, Sébastien Descamps, W. James Grecian, A. Rus Hoelzel, Eleanor M. Honan, Anna S. Rix, Henri Robert, Vegard Sandøy Bråthen, Richard A. Phillips

**Affiliations:** 1 Department of Geography, Durham University, Lower Mountjoy, South Road, Durham, DH1 3LE UK; 2 Norwegian Polar Institute, Fram Centre, Tromsø, 9296 Norway; 3 Department of Biosciences, Durham University, Durham, DH1 3LE UK; 4 International Polar Foundation, Rue des vétérinaires, 42b/1, Brussels, 1070 Belgium; 5 Norwegian Institute for Nature Research, P.O. Box 5685, Torgarden, Trondheim 7485 Norway; 6 British Antarctic Survey, Natural Environment Research Council, Cambridge, CB3 0ET UK

**Keywords:** Climate change, Palaeoceanography, Resource tracking, Seabird tracking, Sea ice, Sentinels, Sexual segregation, Stomach oil

## Abstract

**Background:**

Pelagic seabirds forage over vast areas, and their movements and diet provide valuable insights into environmental conditions that are otherwise difficult to observe. Snow petrels *Pagodroma nivea* forage largely on sea-ice-associated prey, rendering the energy-rich lipids into stomach oil, some of which is spat defensively at nest sites where it accumulates over tens of millennia. These deposits contain chemical signatures of the foraging environment, providing a unique biological archive of sea-ice conditions in the pre-satellite era. Accurate interpretation of these proxies, however, requires detailed knowledge of foraging ranges—how far the petrels travel, the habitats they target, and how these behaviours vary with season, colony location, and sex.

**Methods:**

To estimate foraging ranges at three colonies located 180–200 km inland in Dronning Maud Land, we tracked 94 snow petrels (34 with light-based geolocators and 60 with GPS loggers). We tested whether foraging latitude is associated with the latitude of the ice edge, estimated via satellite remote sensing. We then projected potential foraging ranges for all known colonies in the study area to reexamine assumptions made in paleoclimate studies.

**Results:**

During most breeding stages, and across breeding seasons, core foraging areas were centred approximately 2° south of the outer sea-ice edge and tracked this habitat as it receded during the spring melt. Female snow petrels were approximately 7% lighter than males but foraged at similar distances and in similar areas. Foraging ranges differed little between colonies but substantially between breeding stages. For example, average median range was ~1400 km (95% CI 1340–1470 km) during the pre-laying exodus vs. ~530 (430–660) km during brood-guard.

**Conclusions:**

Snow petrel stomach-oil deposits potentially integrate environmental conditions over greater and more seasonally variable areas than previously assumed, probably with a bias towards conditions in the marginal ice zone (outer pack ice) during the early summer when stomach oil deposition due to nest competition is likely greatest. Our results are consistent with the hypothesis that snow petrel breeding range in the western Weddell Sea is limited by access to foraging habitat, such as coastal polynyas. Although tracking data from other colonies would be useful to confirm the generality of our foraging range estimates, we hypothesise that as sea ice fluctuated over previous glacial-interglacial cycles, this regulated breeding distribution across the region.

**Supplementary information:**

The online version contains supplementary material available at 10.1186/s40462-025-00609-7.

## Background

Knowledge of animal movements and distributions is fundamental to ecology and wildlife management. Breeding seabirds are central-place foragers, alternating between foraging at sea and courting, defending their nests, incubating their egg(s) and brooding, guarding or feeding their offspring. These duties place energetic and temporal limits on foraging range [1–3], defined here as the distance that a seabird can travel from the nest to gather food while breeding successfully. If foraging range is known, seabird diet and behaviour can be used to infer environmental conditions within a known area at sea. A promising new example is the analysis of stomach-oil deposits from snow petrels *Pagodroma nivea* to infer past climatic and biological conditions around Antarctica [4–6].

Snow petrels breed in the austral summer in cavities in or among snow- and ice-free rocks [7, 8]. At sea, they are highly pagophilic, foraging mainly in association with sea ice [9–11]. Throughout their range, their diet is dominated by pagophilic fish, especially the myctophid *Electrona antarctica* and the nototheniid *Pleuragramma antarctica*, with lesser proportions of zooplankton, cephalopods and carrion [8, 12–15]. Like other Procellariformes, their proventriculus is adapted to separate and retain the energy-rich lipid fraction of their prey both for their own sustenance, and to feed to their chick [16, 17]. They also spit stomach oil at conspecifics during agonistic competition for nest sites and as a defence against their main predator, the south polar skua *Stercorarius maccormicki* [8, 18]. Over periods sometimes > 50,000 years, stomach oil builds up around nest sites in well-stratified, waxy accretions up to tens of cm thick [19–21]. Depending on deposit accumulation rate, each layer of a few millimetres can reflect decades or centuries. Stable carbon and nitrogen isotope analysis and lipid biomarkers have been used to reconstruct temporal variation in diet and, because only some prey are pagophilic, to infer past sea-ice conditions [4, 5]. Sea-ice extent, as well as the timing of ice-sheet advance or retreat in the nesting locality have also been inferred from deposit accumulation rates, which reflect temporal variation in nest occupation [22, 23]. Potentially, these techniques could fill an information gap on sea-ice extent in the pre-satellite era, which is a major source of uncertainty in climate models [24, 25], particularly south of the Antarctic Polar Front [26–28]. However, the utility of stomach-oil deposits as climate proxies is currently limited by lack of data on snow petrel foraging ranges. This also hampers understanding of the effects of the availability and accessibility of sea-ice foraging habitats on their past, current and future nesting distribution [29, 30].

Potential foraging range (i.e., the distance reachable in the absence of extrinsic factors) is ultimately limited by morphological and physiological constraints on locomotion and maintenance of body condition, modulated by temporal and energetic demands that vary across the breeding cycle [1, 31], and with sex, especially among size-dimorphic species [32, 33]. Realised foraging ranges are the product of further modulation by extrinsic effects, including the distribution of prey and suitable foraging habitats [34], the effects of wind on flight costs [31], and intraspecific competition [35, 36].

Snow petrels usually forage in areas of intermediate (~30–60%) sea-ice concentration (SIC), i.e. those typical of the marginal ice zone (MIZ) [9–11, 37], which is defined physically as the outer area of pack ice into which ocean wave energy penetrates, fragmenting floes [38]. Snow petrels breed from October to March [8], and Antarctic sea ice is at its maximum and minimum extents in September and February, respectively [39]. Hence, if snow petrels track the MIZ over the spring melt, they should forage further south, with decreasing foraging range, as the breeding season progresses and the MIZ retreats towards the coast [40–42]. Breeding distribution is expected to be limited by distance to suitable foraging habitat, such as the MIZ [43, 44]. Ship-based surveys suggest that the highest densities during breeding are within ~400 km of colonies [10, 42], but recent tracking indicates foraging ranges of 100s to 1000s of km [11, 45]. Variation in foraging range over the breeding cycle, the effects of taking indirect routes due to the constraints of topography, wind, etc. [31, 46], and whether there is spatial segregation among colonies with potentially overlapping foraging areas are all unknown. Similarly, little is known about variation in foraging range with sex other than one study which showed that although snow petrels are sexually size dimorphic, incubating males and females had similar foraging ranges [11].

**Fig. 1:**
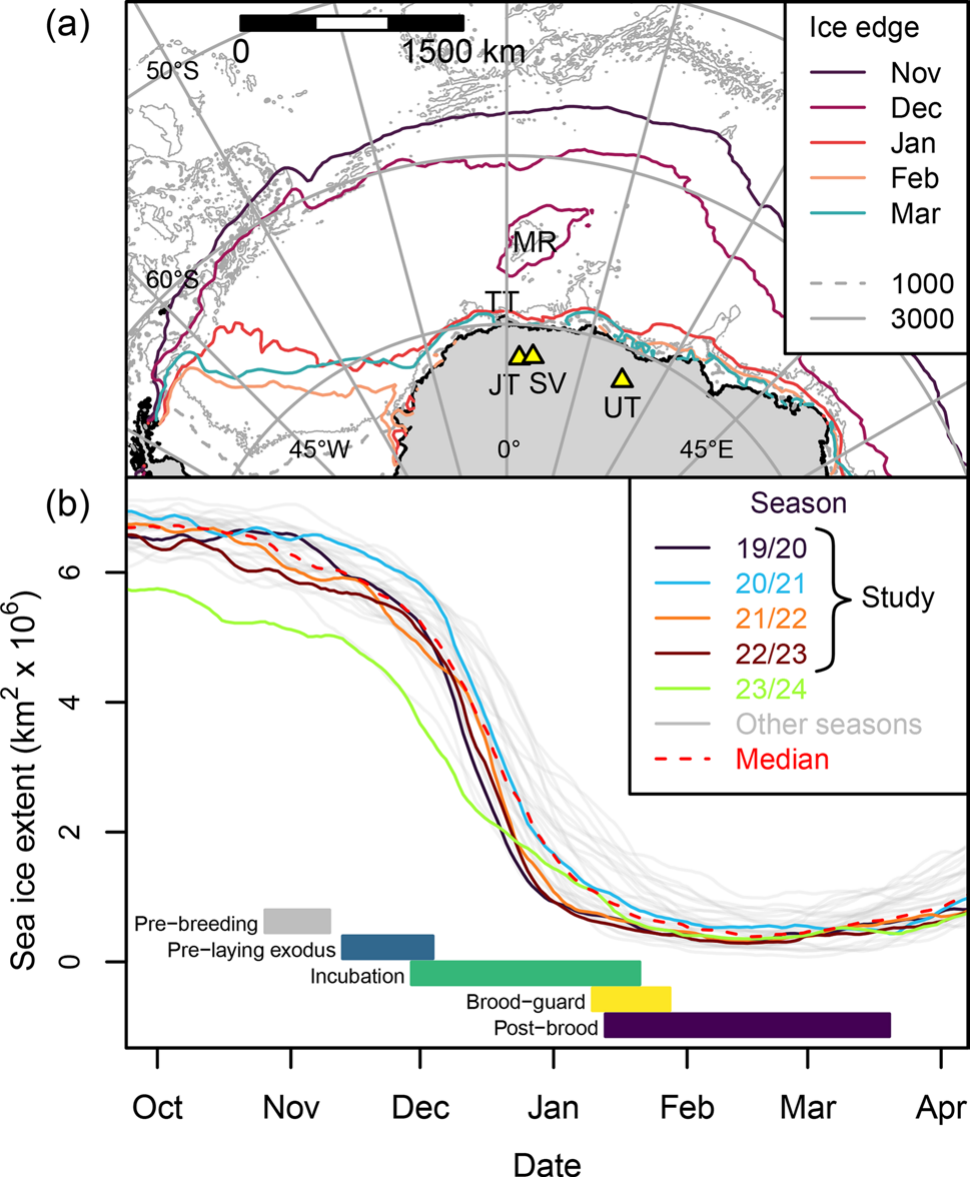
(**A**) Study area showing the 30-year monthly median ice edge during the snow petrel breeding season, study colonies (yellow triangles), and places mentioned in the text (JT Jutulsessen; MR Maud Rise (location of seasonal polynya); TT troll ice Tongue; SV Svarthamaren; UT Utsteinen). (**B**) Evolution of sea-ice cover in the sector 040°W to 040°E during each of the 30 snow petrel breeding seasons from 1994/95 to 2023/24, in relation to the snow petrel breeding cycle. Sea ice metrics derived from National Snow and Ice Data Centre data [47]

The Weddell Sea sector of the Southern Ocean (Fig. 1) is important in terms of sea-ice-climate linkages [48]. Snow petrels breed in its hinterland throughout Dronning Maud Land (DML), and west to the Theron Mountains and Shackleton Range [7]. Stomach-oil deposits from the region have been analysed to infer past occupation history [20, 21], variation in sea-ice conditions since the last glacial period [5, 6, 49] and ice-sheet thinning history [22, 50], but foraging ranges of snow petrels breeding in the region are poorly known [51]. Recent loss of seasonal sea ice has been particularly pronounced in the Weddell Sea sector [52, 53] – a trend predicted to continue under anthropogenic climate change [25, 54]. Development of marine protected areas in the Weddell Sea is currently hampered by a lack of data [55]. As such, better information on foraging range would allow the identification and protection of areas most likely to be used by snow petrels, both now and in the future [56, 57].

## Methods

### Aims

Here, we aim to estimate the foraging ranges and distributions of snow petrels, and test whether they vary with breeding stage, sex and colony. To do so, we tracked birds from three colonies in DML. We illustrate the application of our results for the interpretation of climate proxies derived from stomach-oil deposits, and the relationship between sea-ice conditions and breeding-site occupation, by projecting potential foraging ranges of snow petrels from colonies throughout the Weddell Sea region.

### Study system and data collection

The coastline of DML (20°W-45°E) principally comprises ice shelves which occlude the underlying bedrock [55], and the snow petrels nest 100 - 290 km inland on a discontinuous chain of nunataks, running parallel to the coast [7, 58]. Two forms of snow petrel that differ in size -greater and lesser - have been recognised [59], but these intergrade and their taxonomic status is unresolved [60]. Birds breeding in DML are all considered to be of the lesser form [8]. We tracked breeding snow petrels from three colonies located 203, 183 and 190 km from the coast (Fig. 1): Jutulsessen (72°02.1’ S, 2°28.2’ E; ~3000 pairs); Svarthamaren (71°53.4’ S, 5°09.6’ E; ~2000 pairs); and Utsteinen, (71°57.7’ S, 23°20.1’ E~200 pairs) [61, 62, authors’ unpub. data]. Jutulsessen and Svarthamaren are Important Bird Areas and the latter is an Antarctic Specially Protected Area (ASPA) [63].

The breeding schedule of snow petrels (Fig. 1) is similar across Antarctic continental breeding sites [8, 45, 64, 65]. During the spring melt, sea ice off DML recedes southward in the east of the region and south-westwards in the west (Fig. 1a). Open water often occurs over the Maud Rise before the outer ice edge has receded to this feature and breakup is most rapid during December. Minimum sea-ice extent is reached in February (Fig. 1b), when open water occurs along much of the DML coast, but sea ice persists in the western Weddell Sea year-round (Fig. 1a).

Due to logistical constraints, fieldwork was feasible only between late November and late February, i.e. incubation through to mid chick-rearing. Combining GPS loggers (late breeding season) and light-based Global Location Sensors (GLS) allowed bird movements to be tracked throughout the entire breeding period. In our study system, light-based geolocation was feasible from early October to mid-November and early February to mid-March. It was precluded around midsummer by continuous daylight in potential snow petrel foraging areas, and around the equinoxes by the similarity in daylength across latitudes (Fig. S1). The location accuracies of GPS and GLS tracking are approximately ±20 m (PathTrack, unpub. data) and ±200 km, respectively [66–68].

We caught breeding adults at the nest, weighed them (±2.5 g), removed three contour feathers for molecular sexing (see Supplementary Methods) and attached either a GPS or GLS logger. We attached NanoFix GEO remote-download GPS loggers (Pathtrack Ltd., Otley, UK; 3.7 ± 0.3 g (range 3.1–4.1 g); 43 × 15x 9.5 mm, plus 50 mm whip antenna; most fitted with a solar panel) to the base of the middle two rectrices using Tesa tape and C65-SUPER combined GLS-immersion loggers (Migrate Technology, Cambridge, UK; 1 g; 14 × 8 × 6 mm) to the left tarsus using a plastic ring. We deployed GPS loggers at Svarthamaren during incubation, brood-guard and post-brood (2022/23) and at Jutulsessen during post-brood (2024); and GLS loggers at Svarthamaren and Utsteinen between 2019 and 2023 (Table 1). Mean masses of GPS and GLS loggers plus attachment materials were 2.0 ± 0.3% (range 1.3–2.7) and 0.8 ± 0.1% (range 0.5–1.0) of body masses, respectively. Total handling time was 7 ± 2 minutes (range 4–13).

**Table 1:** Numbers of tracking devices deployed on snow petrels at colonies in Dronning Maud Land and from which data were recovered, 2019/20–2023/24

Season	Colony	N deployed, recovered (female, male, unknown)	
		GLS	GPS
2019/20	Svarthamaren	20, 0	
2021/22	Svarthamaren	6, 0	
	Utsteinen	12, 0	
2022/23	Svarthamaren	15, 5 (0,3,2)	44, 36 (11,25,0)
	Utsteinen	0, 7 (4,3,0)	
2023/24	Svarthamaren	0, 22 (2,8,12)	
	Jutulsessen	0, 0	31, 24 (11,13,0)

GLS loggers recorded maximum light intensity every five minutes and wet-dry state either as the number of samples within ten-minute blocks when the logger was immersed, based on a test every 30 s, or the times of state changes between wet and dry that lasted ≥6 s. GPS loggers acquire a fix every 30 minutes when the battery was fully charged or at longer intervals when it was depleted. If possible, GPS loggers deployed during incubation were recovered that season. Those deployed during chick-rearing were not recovered but continued to transmit data to base stations until the tags failed or were lost. Snow petrels moult their rectrices at the end of the breeding season, or earlier if breeding fails [8], so loggers not recovered would have been shed by April. We recovered GLS loggers after 1–4 y, with recovery attempts in all seasons except 2020/21.

All sea-ice metrics are based on analysis of sea-ice concentrations (SIC) derived from measurements by the satellite Special Sensor Microwave Imager/Sounder instrument and downloaded from the National Snow and Ice Data Centre [69] on a 25 km regular grid [47]. To define the ice edge, we followed the commonly applied operational assumption that this corresponded to the 15% SIC contour [70].

### GLS data processing and colony attendance

Following [71], we used the light level data to obtain twice daily locations estimates (see Supplementary Methods). In brief, we used the threshold method to estimate twilight times, and calculated latitude from the inter-twilight interval and longitude from the time of noon or midnight. We did not attempt to estimate locations during equinoctial periods or during bouts of daylight exceeding 24 h (i.e. when birds were at latitudes of > 70°S during midsummer). We filtered both twilights and positions to reduce location errors.

Prior to analysis, we reduced activity data from all loggers to the proportion of each ten-minute period immersed. We then defined putative periods in the colony as those during which loggers were dry > 95% of each 24 h period and bouts on the nest as those in which loggers were shaded (based on the light data) throughout most of the day. We assumed that birds still carrying out nest shifts after January 1^st^ hatched their chick successfully that season and considered all other birds to have been breeding up to the end of their last recorded incubation shift. For subsequent analyses, we retained only locations at sea, defined using the High Resolution Vector Polygons of the Antarctic Coastline (7.6) dataset [72].

### GPS data processing and behavioural classification

Prior to analysis, we split GPS tracks into foraging trips and interpolated locations to 30-minute intervals using a correlated random walk model (Supplementary Methods). The higher resolution of GPS than GLS data also allowed us to exclude bouts of non-foraging behaviours prior to estimation of foraging range, as these potentially occur non-uniformly with respect to distance from the colony. To do so we followed Wakefield et al. [40], using Hidden Markov Models to classify movement based on step length and turning angle as either travelling (step length high, turning angle concentrated), foraging (step length intermediate, turning angle dispersed) or resting (step length low, turning angle very dispersed) [73, 74]. In brief, we implemented models in moveHMM package [75]. We fitted HMMs 25 times, randomly drawing starting parameters from within plausible ranges based on previous studies [40, 76] (Table S1). Using the model with the highest likelihood [77], we predicted the most likely sequence of states using the Viterbi algorithm, retaining only the putative foraging locations for subsequent analyses.

### Estimation of space use and comparisons among groups

To summarise space use, we used utilisation distributions (UDs) estimated via kernel analysis [78]. To avoid possible underestimation of the size of used areas by this technique [79], we used the ctmm package to apply autocorrelated-kernel density estimation to the foraging locations derived from GPS data [80]. We first used the ctmm.select() function to fit a continuous time movement model to foraging locations of each bird and then akde() to estimate their UDs. We then combined these using the pakde() function [81] to estimate population-level UDs and their associated uncertainties for each breeding stage, and where relevant, sex.

The ctmm() package is not currently configured to process GLS data, which are characterised by relatively large and anisotropic errors. We therefore used conventional kernel density analysis to estimate UDs for birds tracked using GLS loggers, assuming that bias due to serial autocorrelation would be low because only 2 locations/day/bird were recorded. To incorporate location errors, we randomly generated 100 locations for each observed location [82], assuming Gaussian latitude and longitude errors reported for seabirds tracked using similar light-based geolocation techniques in a similar geographical area [66]. We used the adehabitatHR package [83] to estimate UDs grids for birds within stages, then averaged the UD within each grid cell across birds to obtain population mean UDs for each stage. Hereafter, we refer to the core foraging area and (general) foraging area as those containing the first 50% and 95% of the cumulative utilisation distribution, respectively. To facilitate comparison of foraging areas across stages, we specified a fixed smoothing parameter *h* for UDs from GLS data of 100 km. We selected *h* ad hoc [84] by identifying the minimum value that resulted in ≤ 2 contiguous 95% UD contour polygons in each breeding stage. As UDs for birds tracked with GPS and GLS were estimated using different methods, their extents are not directly comparable.

We used two approaches to compare overlap of foraging distributions among breeding stages, sexes and colonies. For both GLS and GPS-tracked groups, we calculated the Home Range Overlap Index, *HROI* [35], defined here as the mean of the proportion of the core foraging area of each group intersected by that of the other group:

1 $$HROI = {1 \over 2}\left( {{{{A_{i,j}}} \over {{A_i}}} + {{{A_{i,j}}} \over {{A_j}}}} \right),$$

where ${A_i}$ and ${A_j}$ are the areas of group *i* and *j*’s core foraging areas and ${A_{i,j}}$ is the area of their intersection (cf. Fieberg and Kochanny’s *HR* index [85]). *HROI* ranges from 0 (no overlap) to 1 (complete overlap). We tested the null hypothesis that observed core foraging areas did not differ between groups (male vs. female or colony 1 vs. colony 2) by randomly shuffling bird identities across the two groups without replacement and recalculating *HROI* 1000 times, then calculating the *p* value as the proportion of null *HROI* values that were less than the observed value, considering this a one-sided test [11, 86]. For GPS-tracked groups, we also used the used the overlap() function in ctmm package to calculate Bhattacharyya’s coefficient (BC), and its associated uncertainty.

2 $$BC = \sum\nolimits_{{\rm{All\,}}x} \sqrt {{{\widehat {UD}}_i}\left( x \right){{\widehat {UD}}_j}\left( x \right)} $$

where ${\widehat {UD}_i}$ and ${\widehat {UD}_j}$ are the observed UDs of birds from groups *i* and *j* on a grid comprising cells *x* and ranges from 0 (complete dissimilarity) to 1 (identical). The overlap() function propagates uncertainty due to serial autocorrelation and corrects for a tendency towards negative bias in BC at small effective sample sizes [87].

### Foraging range estimation, comparison, and projection

When commuting between the colony and the coast, birds followed beeline tracks modified by wind drift similar to those of sympatric Antarctic petrels *Thalassoica antarctica* described by Tarroux et al. [88] (see Sect. 4.3). Once at sea, snow petrels largely avoided crossing land or ice shelves, even when travelling to destinations that could have been reached more directly over land. Moreover, presumably due to prevailing easterly winds, they crossed the coast further west on the outward leg than on the inward leg of most foraging trips (see Results). We therefore used biological distance – i.e., distance via the shortest paths conforming to these constraints [46], to quantify foraging range. We approximated the biological distance *d* from the colony to a location *x* as

3 $${d_x} = \left( {{d_{c,{s_1}}} + {d_{{s_1},x}} + {d_{x,{s_2}}} + {d_{{s_2},c}}} \right)/2,$$

where ${d_{c,{s_1}}}$ is the great circle distance from the colony to ${s_1}$, the location at which the outward commuting bird crosses the coast; ${d_{{s_1},x}}$ is the distance by sea from there to *x*; ${d_{x,{s_2}}}$ is the is the distance by sea from *x* to ${s_2}$, the inward coast crossing; and ${d_{{s_2},c}}$ is the great circle distance from there to the colony (Fig. S2). We calculated ${d_{{s_1},x}}$ and ${d_{x,{s_2}}}$ on a 3.125 km regular grid using the gdistance package [89]. Location resolution was insufficient to determine where birds tracked with GLS crossed the coast, so we assumed they did so at the median longitudes of birds tracked with GPS.

For each individual, we then quantified foraging range as the median ($${d_{50}})$$, 95^th^ percentile ($${d_{95}})$$ and maximum $$({d_{{\rm{max}}}})$$ biological distance of at-sea locations within each foraging trip within stages for GPS-tracked birds or each breeding stage for GLS-tracked birds (GLS data resolution was insufficient to discriminate individual foraging trips). For GPS-tracked birds, we use the subscript *f* to denote statistics that summarise *d* across foraging locations (e.g., $${d_{f,50}}$$, etc.), whereas for GLS-tracked birds we use the subscript *u*, denoting all forms of utilisation but assume that these largely reflect foraging distribution. We defined the mean heading during overland commutes between the colony and the first location at sea as $${\theta _{out}}$$, and between the last location at sea and the colony as $${\theta _{in}}$$.

To summarise foraging distances and test for differences among breeding stages, etc., we analysed the GPS and GLS datasets separately. For the GLS dataset we had one distance per bird, so we fitted a Generalised Linear Models (GLM). The GPS dataset contained multiple trips per bird, so we fitted Generalised Linear Mixed Models (GLMM) in the lme4 package [90], treating Bird ID as a random intercept. Foraging ranges were strictly positive and right-skewed, so we specified either inverse-Gaussian error distribution with an inverse link function or we log-transformed the response and specified Gaussian errors and an identity link function. We also scaled the response by dividing by standard deviation prior to model fitting. We checked conformity to model assumptions using Q-Q and residual plots and we carried out multiple comparisons between breeding stages and calculated marginal mean foraging ranges and their 95% confidence intervals using the emmeans package [91]. For simple two-sample or paired data, we used *t* or Wilcoxon tests, checking equality of variance and normality using *F* and Shapiro-Wilk tests. We estimated repeatability *R* [92] in foraging range using the rptR package [93]. In almost all cases, test for differences in foraging range among groups led to the same conclusions whether they were carried out on median, 95^th^ percentile or maximum foraging ranges. For brevity, we therefore only present results for tests on median foraging range unless the 95^th^ percentile or maximum foraging ranges had different significant effects.

Given recent declines in Antarctic sea-ice extent and the possible links between foraging rage and the location of the ice edge, we checked whether conditions during our study were representative in terms of climatic conditions. For each day within each breeding stage, for 30 years prior to the end of the study, we calculated the mean biological distance between each study colony and all SIC cells intersecting the 15% SIC contour within 2330 km of that colony (the latter is the maximum observed foraging range). We then calculated the annual mean across days within breeding stages for each colony and graphically compared study years to the climatic means.

To illustrate the potential foraging ranges of birds from other colonies in the study area (defined as the Antarctic continent between 30ºW and 25ºE), we obtained colony coordinates from Francis et al. [94]. We then calculated rasters of biological distances from those locations using the methods outlined above, assuming that outward and return flight directions of birds foraging from colonies in the Antarctic interior were 317º and 173º, respectively. We then extracted distance contours corresponding to the foraging ranges in Table 2.

**Table 2:** Foraging ranges^1^ (km) of snow petrels tracked from three colonies in Dronning Maud in 2020/21–2023/24

Tracking Method^2^	Stage	Season	Colony^3^	N	Mean (95% confidence interval; range)^4^		
					Median	95^th^percentile^5^	Maximum^5^
GLS	Pre-breeding	2020/21	SV	5	1739 (1529–1979; 1491, 2091)	1949 (1738–2185; 1704, 2248)	2008 (1794–2247; 1727, 2332)
		2021/22	SV	5	1876 (1649–2134; 1486, 2864)	2100 (1873–2355; 1790, 3072)	2138 (1910–2393; 1826, 3077)
		2022/23	SV	8	1652 (1492–1829; 1418, 1902)	1883 (1721–2062; 1630, 2030)	1942 (1776–2122; 1660, 2189)
		2022/23	UT	7	1636 (1467–1825; 1424, 1797)	1836 (1667–2023; 1536, 2096)	1859 (1691–2045; 1544, 2099)
		2023/24	SV	22	1229 (1155–1306; 836, 1471)	1416 (1341–1496; 989, 1673)	1457 (1381–1537; 1109, 1703)
		**All**	**All**	**47**	1611 (1534–1691; 836, 2864)	1821 (1744–1902; 989, 3072)	1865 (1787–1946; 1109, 3077)
	Pre-laying exodus	2020/21	SV	5	1667 (1463–1899; 1507, 1804)	1886 (1659–2145; 1811, 1958)	1935 (1696–2208; 1875, 1970)
		2021/22	SV	5	1308 (1148–1490; 1061, 1476)	1373 (1207–1561; 1111, 1575)	1391 (1220–1587; 1148, 1612)
		2022/23	SV	15	1413 (1310–1523; 923, 1881)	1590 (1476–1712; 936, 2020)	1615 (1496–1742; 938, 2102)
		2022/23	UT	7	1420 (1272–1586; 1326, 1526)	1578 (1416–1759; 1374, 1724)	1624 (1453–1816; 1382, 1731)
		2023/24	SV	22	1245 (1170–1325; 933, 1684)	1405 (1322–1494; 1082, 1850)	1436 (1348–1529; 1103, 1857)
		All	All	54	1403 (1339–1471; 923, 1881)	1556 (1486–1630; 936, 2020)	1589 (1515–1667; 938, 2102)
	Post-brood^6^	2019/20	SV	2	981 (486–1981; 625, 1540)	1239 (693–2217; 915, 1678)	1302 (726–2337; 986, 1720)
		2020/21	SV	4	1099 (669–1806; 738, 1550)	1420 (941–2142; 1049, 1842)	1572 (1039–2376; 1302, 1940)
		2021/22	SV	3	730 (411–1295; 569, 1170)	1202 (748–1933; 922, 1458)	1479 (917–2383; 1415, 1553)
		2021/22	UT	3	1552 (874–2754; 1285, 1830)	1953 (1215–3139; 1720, 2215)	2008 (1246–3237; 1791, 2272)
		2022/23	SV	19	831 (662–1044; 482, 2325)	1121 (929–1354; 612, 2483)	1190 (984–1438; 628, 2644)
		All	All	31	1003 (788–1276; 482, 2325)	1359 (1113–1659; 612, 2483)	1485 (1215–1815; 628, 2644)
GPS	Incubation	2022/23	SV	27	861 (727–1022; 388, 1626)	1072 (928–1238; 587, 1746)	1090 (944–1260; 602, 1782)
	Brood-guard	2022/23	SV	8	533 (428–665; 377, 726)	620 (539–712; 538, 787)	625 (545–717; 543, 792)
	Post-brood^7^	2022/23	SV	11	597 (507–704; 291, 1011)	751 (643–877; 389, 1439)	761 (655–884; 424, 1447)
		2023/24	JT	24	713 (651–779; 258, 1485)	831 (766–901; 300, 1770)	843 (779–913; 300, 1781)
		All	All	35	652 (592–718; 258, 1485)	790 (719–867; 300, 1770)	801 (731–878; 300, 1781)

^1^Shortest distance from the colony, given movement constraints (see Methods). ^2^ For GLS- and GPS-tracked birds, distances refer respectively to all at-sea locations or foraging locations only. ^3^ JT = Jutulsessen, SV = Svarthamaren, UT = Utsteinen. ^4^ Calculated at the individual (GLS data) or trip level (GPS data), then marginal means and 95% confidence intervals estimated using Generalised Linear Models. ^5^ May be inflated due to geolocation error. ^6^ Observed Feb 5^th^ to Mar 16^th^. ^7^ Observed 19^th^ Jan −1^st^ Mar

## Results

### Sample sizes, breeding phenology and sea-ice conditions

**Fig. 2:**
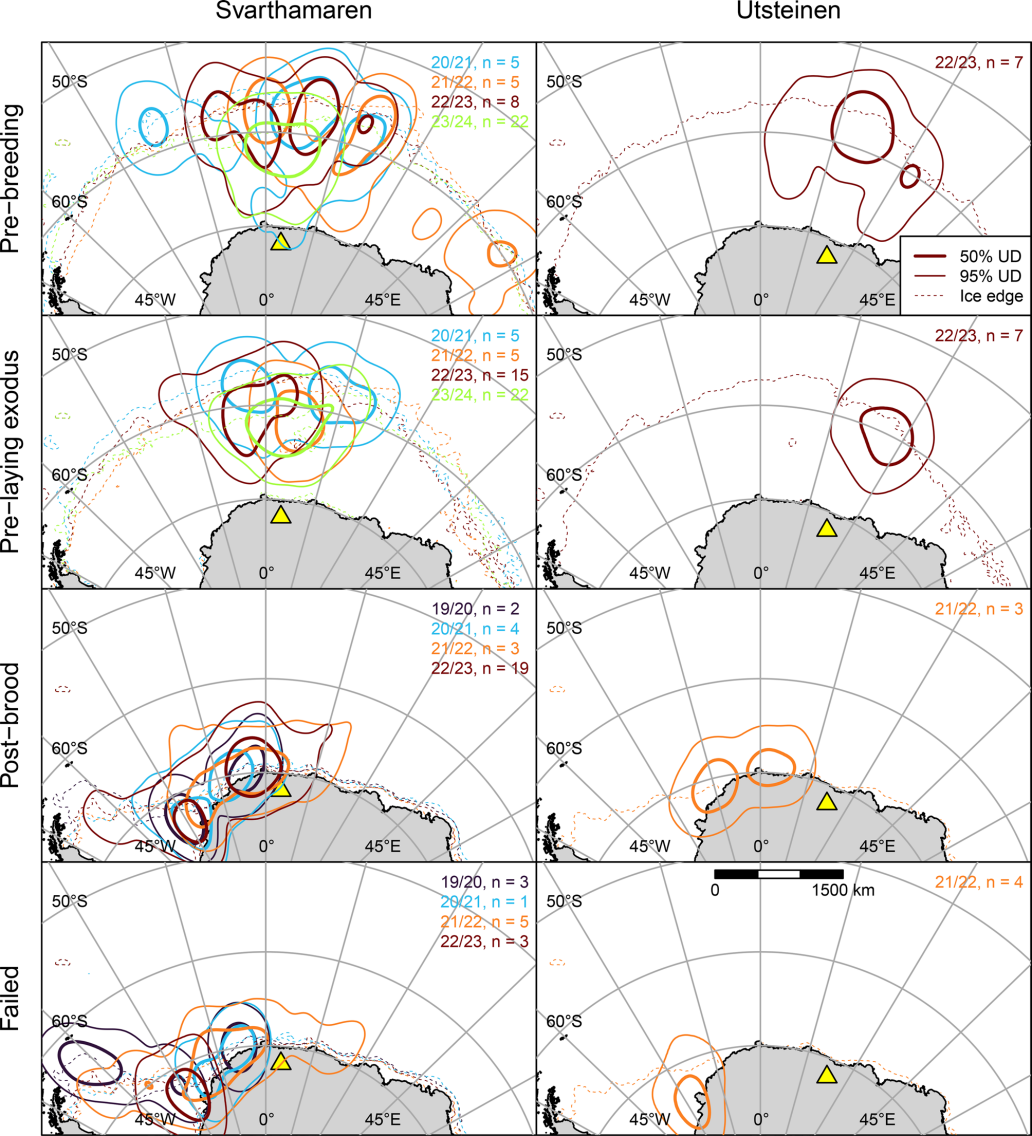
Approximate foraging areas and core foraging areas (50 and 95% cumulative utilisation distributions (UDs), respectively) of snow petrels tracked via light-based geolocation during three breeding stages (rows) from two colonies (columns) in Dronning Maud Land in five breeding seasons (colours). *N* = number of birds tracked in each stage/season, plus failed breeders. Yellow triangles show study colonies. Sea ice metrics derived from National Snow and Ice Data Centre data [47]

We deployed GLS loggers on 53 birds, the majority (41 birds) at Svarthamaren, and retrieved data from 34 (Table 1), tracked for a median of 307 d (IQR 301 – 351 d; range 287 – 1462 d). Data coverage was biased towards the pre-laying exodus, and the last two study seasons. We deployed GPS loggers on 75 birds (Table 1) and retrieved data from 60 birds, the majority from incubation at Svarthamaren in 2022/23 and post-brood at Jutulsessen in 2023/24 (Fig. 2). Birds were tracked by GPS for a median of 2 trips (IQR 1–3; max 9) over 10.8 d (IQR 7.2–19.7; range 2.9, 40.3) at an interval of 30 min. (IQR 30–60; range 30, 120). Across colonies, females were 16 g (95% CI 4–28 g) lighter than males (Tables S2 & S3) and female mass was lower at Jutulsessen (232 ± 24 g) than at Svarthamaren and Utsteinen (~258 ± 32 g at both).

Compared to the 30-year average, spring sea-ice retreat occurred relatively early during the 2019/20, 2021/22 and 2022/23 seasons (Fig. 1). However, mean distance from the ice edge to the study colonies was generally within ~1 sd of the climatic mean in all study seasons/stages, except 2023/24 (Fig. S3). The latter season commenced with sea-ice extent ~1 million km^2^ lower than average. In consequence, the ice edge during pre-breeding, pre-laying exodus and incubation, respectively, was ~185, 250 and 90 km closer to study colonies.

Based on GLS data, the pattern of first nest attendance was similar across individuals and colonies: Excluding one individual that attended its nest from 14 to 16 October, the first full day spent on the nest was 07 November ±3 d (range 03 November − 16 November) and did not differ between Svarthamaren and Untsteinen (*t*(32) = 0.578, *p* = 0.567). Most individuals experienced 24 h daylight for 1 d prior to this, suggesting that they arrived at the latitude of the colony a day before entering the nest cavity. The majority (82%) of birds spent one bout (9.1 ± 2.8 d) at the breeding site prior to incubation, with males spending 3.8 d longer than females (*t*(18) = −7.217, *p* < 0.001). Respectively, 12 and 6% of birds undertook a second and third pre-laying bout (2.5 ± 0.8 d and 3.5 ± 2.1 d). The first bout on the nest was 3.8 d longer among males than females (*t*(18) = −7.217, *p* < 0.001). The pre-laying exodus began on 18 November ±5 d (range 11 November − 04 December) and the first incubation shift on 04 December ±4 d (range 30 November − 11 December). Direct nest monitoring at Svarthamaren (*n* = 78 nests) recorded laying on 04 December ±4 d and hatching on 14 January ±4 d. Mean (range) trip durations of GPS-tracked birds were: incubation 7.0 ± 2.5 (2.9–11), brood-guard 3.8 ± 0.7 (2.9–4.7) and post-brood 4.6 ± 1.2 (2.1–7.5) d.

### Space use and foraging range

Most GLS-tracked snow petrels spent the pre-breeding period and pre-laying exodus in a 45° sector north of the respective colonies (Fig. 2). Core foraging areas in both stages were centred around 100 km south of the ice edge. Within these stages, core foraging areas of birds from Svarthamaren overlapped partially among seasons (*HROI* ranges from 0.14 to 0.44 for pre-breeding and 0.05 to 0.68 for pre-playing exodus, Fig. S4). During spring 2022, when birds from both Svarthamaren and Utsteinen were tracked, core foraging areas of the respective populations overlapped very little during pre-breeding (*HROI* = 0.04) and not at all during the pre-laying exodus (Fig. 2). However, within these stages, foraging range (Table 2) did not differ significantly between colonies (${d_{u, 50}}$ Svarthamaren vs Utsteinen pre-breeding, *t*(13) = 0.234, *p* = 0.819; pre-laying exodus, *t*(19.3) = 0.135, *p* = 0.894) so we aggregated foraging range across colonies for comparisons among years. In addition, as individual repeatability within life-history stages for birds that were tracked in multiple seasons was low (*R* for ${d_{u, 50}}$ ≤ 0.3), we treated repeated measures on individuals across years as independent. Within these stages, ${d_{u, 50}}$ varied significantly among years (Table S4). Prior to breeding, birds used locations approximately 530 km closer to their colonies in 2023/24 than in other seasons. During the pre-laying exodus, ${d_{u, 50}}$ was approximately 250–420 km lower after 2020/21. For birds tracked during both pre-breeding and the pre-laying exodus, ${d_{u, 50}}$ was ~170 km lower (*t*(14) = 3.361, *p* = 0.005) during the former stage in 2022/23 but did not differ between these stages in 2023/24 (*t*(21) = −0.459, *p* = 0.651). Among 20 known-sex birds, males foraged closer to the colony than females by ~ 220 km during the pre-laying exodus (*t*(18) = 2.439, *p* = 0.025) and ~210 km during the pre-breeding period, but the significance of the latter was marginal (*t*(18) = 2.085, *p* = 0.052).

Birds tracked by GLS from Svarthamaren during late post-brood (05 February to 16 March) concentrated in Weddell Sea coastal waters, from approximately 0 to 38°W (Fig. 2). Foraging area overlap was relatively high among years (*HROI* range 0.46–0.70, Fig. S4) but sample sizes were insufficient to test for differences in $${d_{u, 50}} $$among colonies and seasons (Table 1). Six birds tracked from Utsteinen and Svarthamaren in late post-brood in 2022 used a similar area (*HROI* = 0.71). Failed breeders used areas further west, centred either in the southeast Weddell Sea or in 2020, the northwest Weddell Sea (Fig. 2).

Paths followed by birds tracked with GPS during overland commuting segments of foraging trips were complex (Fig. S5) but headings on outward and inward overland commutes were approximately northwest and south, respectively (Table 3).Hence, birds from Svarthamaren and Jutulsessen crossed the coast on average 167 ± 159 km and 141 ± 141 km further east on the outbound than return commute (one-sample *t*-test, one trip per bird: Svarthamaren *t*(32) = 5.244, *p* = < 0.001; Jutulsessen *t*(23) = 6.641, *p* = < 0.001). Having reached the coast, they largely avoided crossing land or ice shelves again until their inward commute, excepting a few instances when they crossed the Troll Ice Tongue west to east.

**Table 3:** Headings of snow petrels tracked by GPS on overland commuting legs of foraging trips from two colonies in Dronning Maud Land

Colony	Leg (n birds)	Mean heading (ρ) ^1^
Jutulsessen	Outward (24)	313.1° (0.97)
	Inward (24)	166.5° (0.91)
Svarthamaren	Outward (35)	321.8° (0.92)
	Inward (35)	179.6° (0.94)

^1^*ρ* = mean resultant length

**Fig. 3:**
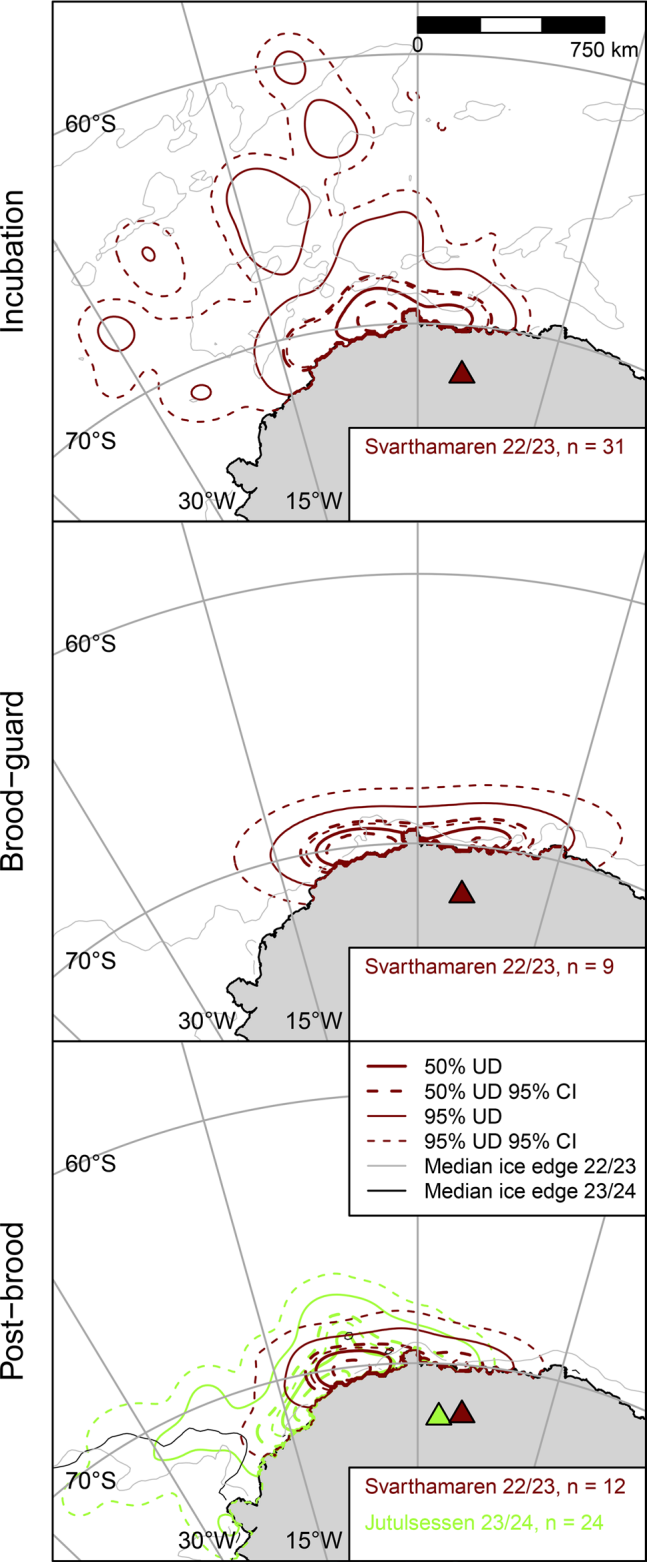
Foraging areas and core foraging areas (50 and 95% cumulative utilisation distributions (UDs), respectively) of snow petrels tracked with GPS during three breeding stages (rows) from two colonies in Dronning Maud Land in two breeding seasons (colours). *N* = number of birds tracked in each stage/season/colony and triangles show study colonies. Sea-ice metrics derived from National Snow and Ice Data Centre data [47]

Where sample sizes were sufficient for testing (Svarthamaren, incubation, 2022/23; Jutulsessen, post-brood, 2023/24), space use and foraging range based on GPS data did not differ significantly between the sexes (Table 4, Fig. S6). Hence, we pooled sexes in subsequent analyses. Among birds tracked by GPS in 2023/24 (early December to late February), foraging range was greatest during incubation, followed by post-brood and brood-guard (Table 2). Differences between these stages were all significant except ${\bar d_{u,50}}$ during brood-guard vs. post-brood (Table 5). During incubation, birds foraged mainly < 200 km from the coastline, between 7°E and 15°W but also made long trips over a wide area as far north as 58° S, up to 1782 km from the colony (Fig. 3). During brood-guard, they foraged nearer the coast, between 0 and 8° E (*HROI*_IN,BG_ = 0.41, *p* = 0.039; *BC*_IN,BG_ (95% CI) = 0.89 (0.58–1.00)). During post-brood, they used a similar core area (*HROI*_BG,PB_ = 0.50, *p* = 0.292; *BC*_BG,PB_ (95% CI) = 0.96 (0.61–1.00)) but also made longer trips to the northwest and west, into the Weddell Sea embayment, as far as 28° W, ≤1447 km from the colony. Post-brood birds GPS-tracked from Jutulsessen in 2024 travelled up to 1781 km from their colony to similar locations to 2023 post-brood birds from Svarthamaren (*HROI*_sv,JT_ = 0.70, *p* = 0.182; *BC*_sv,JT_ (95% CI) = 0.92 (0.65–1.00)).

**Table 4:** Comparison between foraging distributions and median foraging ranges (${\bar{d}}_{u,50}$) of male and female snow petrels tracked with GPS from two colonies/breeding stages in Dronning Maud Land

Group	HROI^1^	BC (95% CI)	${\bar{d}}_{u,50}$ ± sd km (n birds)		
			Females	Males	Test^2^
Svarthamaren (incubation)	0.66, p = 0.478	0.80 (0.30-1.00)	869 ± 310 (11)	847 ± 386 (14)	t = -0.066, p = 0.948
Jutulsessen (post-brood)	0.81, p = 0.393	0.99 (0.85 - 1.00)	658 ± 109 (11)	730 ± 206 (13)	W = 58, p = 0.459

^1^ Probability of core forgaing area overlap determined via randomisation of bird identities; ^2^ Two-sample t-test or Wilcoxon test

**Table 5:** Fixed effects in a generalized linear mixed-effects models of foraging range of snow petrels tracked by GPS from Svarthamaren, Dronning Maud Land in 2022/23 as a function of breeding stage (IN = Incubation, BG = brood-guard, PB = post-brood)

Foraging range	Stage	Parameter	SE	t	p	Tukey contrasts p	
						BG	PB
Median	IN (intercept)	0.207	0.034	6.046	<0.001	<0.001	0.003
	BG	0.237	0.051	4.627	<0.001		0.063
	PB	0.120	0.037	3.281	0.001		
95^th^ %	IN (intercept)	0.172	0.028	6.088	<0.001	<0.001	0.019
	BG	0.268	0.05	5.419	<0.001		<0.001
	PB	0.080	0.03	2.674	0.008		
Maximum	IN (intercept)	0.173	0.028	6.213	<0.001	<0.001	0.014
	BG	0.278	0.049	5.724	<0.001		<0.001
	PB	0.082	0.029	2.803	0.005

### Foraging latitude vs. ice-edge latitude

**Fig. 4:**
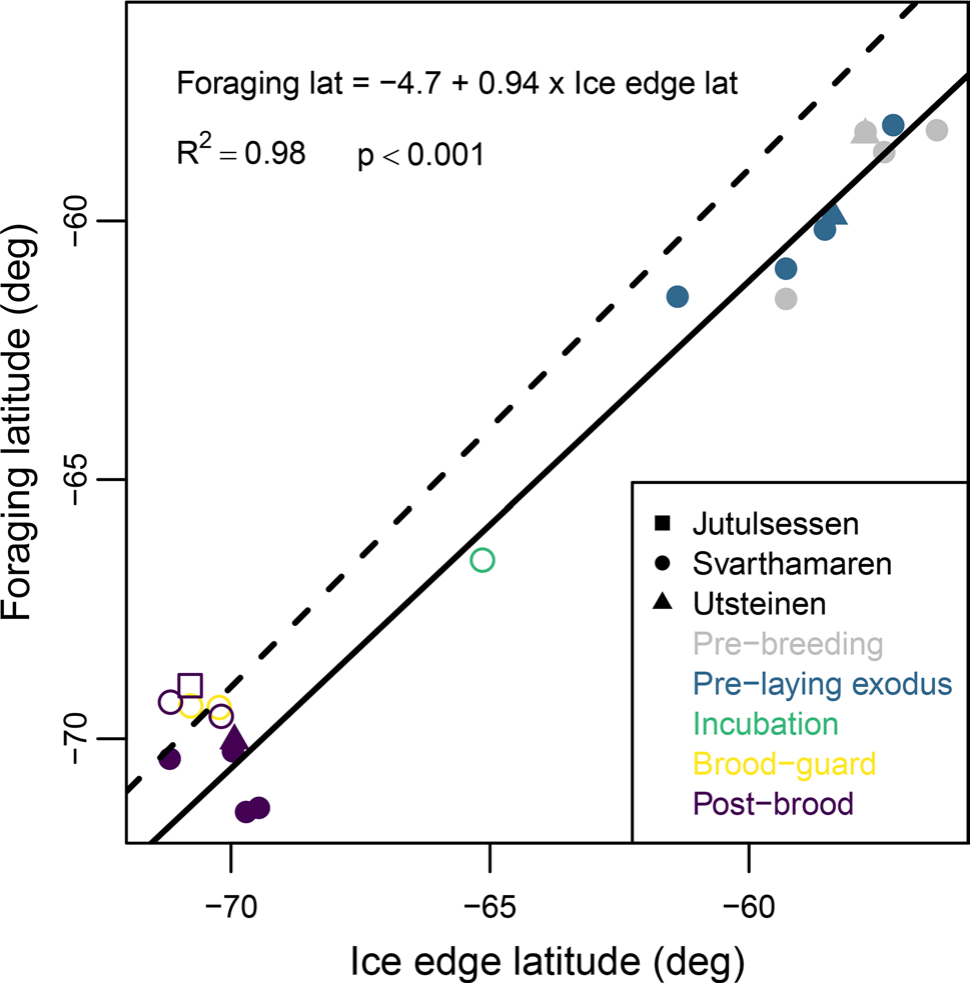
Median foraging latitudes of snow petrels tracked from three colonies in Dronning Maud Land averaged across individuals within stage/colony/season vs. median latitude of the ice edge between 35°W and 25°E during the same periods. The solid line is a linear model fitted to the data from birds tracked with GLS (solid symbols). For comparison, open symbols show data derived from GPS tracking and the dashed line a 1:1 relationship

Across breeding stages and seasons, median foraging latitude was strongly associated with median ice edge latitude (Fig. 4), with a linear model fitted to the GLS data showing that birds foraged approximately 2° S of the ice edge. This was also evident during the pre-breeding and pre-laying exodus stages, but not within post-brood. Median foraging latitude estimated from GPS data followed the same trend during incubation, but during chick-rearing it coincided with the latitude of the median ice edge.

## Discussion

### Comparison with previous studies

Based the distance of breeding sites from suitable foraging habitat, the foraging range of snow petrels has previously been assumed to be at least 480 km [43]. Our data show that even during brood-guard, when birds are most constrained, realised foraging ranges can be ~25% greater. Indeed, the median foraging range of snow petrels varies from ~530 km during brood-guard to ~ 1400 km during the pre-laying exodus, reflecting the relative central-place constraint associated with different breeding stages and seasonal fluctuations in the location of the MIZ, where snow petrels typically forage. The mean foraging range of snow petrels tracked with GLS from Ile des Pétrels, Terre Adélie during the pre-laying exodus and late post-brood was 2648 ± 1054 (max 4978) km [45]. This is around 1000 km greater than we observed in either stage, but the Terre Adélie dataset may have included failed breeders. In contrast, birds tracked via GPS from the same colony during incubation had a median foraging range of only ~120 km [Fig. 1 in 11], ~600 km less than we observed. In part, this may be because Svarthamaren is ~ 175 km further inland but given the relationship we found between meridional foraging range and sea-ice extent, another likely cause is regional differences in sea-ice distribution and dynamics. In December, the median ice edge is approximately 580 km from Svarthamaren but only 40 km from Ile des Pétrels (Fig. 4). In addition, intraspecific competition may force birds from DML to forage further from their colonies because regional population size is apparently relatively high compared to Terre Adélie [7]. Regional differences in interspecific competition or diet could also play a role, but there is insufficient information on these factors in Dronning Maud Land to make comparisons with Terre Adélie [55].

While it has sometimes been assumed that the breeding distribution of snow petrels is limited by distance to the coast [22, 44], it is more likely that distance to the MIZ is limiting, because this is their preferred foraging habitat [10] (Sect. 4.4). During early chick-rearing, when snow petrels are most constrained and sea-ice extent is near its seasonal minimum, the maximum distance between any currently occupied colony and the median 50% SIC contour is 694 km [95]. This is similar to the maximum foraging range that that we observed for this stage (median 608, IQR 570- 655, overall maximum 791 km).

### Study limitations

Our results have several potential limitations. Firstly, the generality of our findings may have been affected by sea-ice conditions. Within breeding stages, the mean distance from study colonies to the ice edge has a standard deviation of ~ 100 km between years (Fig. S3), so we would expect a similar degree of variation among years in foraging ranges. Although sea ice around Antarctica as a whole declined during our study [52, 96], it was anomalously low in our study area only during 2023/24 (Fig. 1, Fig. S3). Hence, though sea-ice extent was very low at the start of that season and the ice edge was unusually near the coast, likely resulting in relatively low foraging ranges prior to brood-guard (Sect. 4.3), foraging ranges during the reminder of the study were likely typical of the long-term mean. Secondly, while our samples sizes were probably sufficient to identify core foraging areas, and by extension presumably median foraging ranges [97], they likely underestimated the foraging area, and presumably therefore 95^th^ percentile and maximum foraging ranges. Tracking of more birds from our study colonies, others would be required to refine our foraging range estimates, which in the meantime should be regarded as provisional. Thirdly, although the tracking devices were relatively small [98], their extra mass and drag could have affected foraging range [99], more likely leading to a reduction, than an increase [100, 101]. Fourthly, during late post-brood, our GLS dataset may have included some failed breeders which were no longer under a central-place constraint, biasing foraging ranges upwards. This would not however have affected our estimate for GPS tracked birds, which were known to be breeding.

Our results for the pre-laying and late post-brood stages derive from light-based geolocation, necessitating several more caveats. GLS errors are approximately bivariate normally distributed [68]. Our estimates of the median foraging range should theoretically therefore be unbiased, but this may not be the case for the 95^th^ percentile and maximum forage ranges derived from GLS data, as these will be sensitive to outliers and should be treated with caution. It is also possible that estimated locations had a systematic bias. Longitudinal bias is unlikely, because this is normally caused by persistent zonal movement in one direction (e.g. migration) [102], whereas birds in our study were commuting regularly in opposite directions. Shading of the light sensor on the logger, for example due to tucking of the legs into contour feathers, could have resulted in underestimation of day length and therefore a systematic northward bias in latitude [102]. Due to the geometry of the study system, this would result in an overestimate of foraging range; however, visual inspection of light curves did not indicate systematic shading. The relatively large size of geolocation errors means that we may have overestimated the extent of foraging areas and therefore overlap between years and colonies. In addition, errors are often greater in latitude than longitude [102, 103]. In our study, this could have increased error in foraging range up until hatching, when birds make long foraging trips with a predominantly meridional displacement. However, calibration studies on other mid-to-high latitude pelagic seabirds report relatively isotropic errors [66, 68]. In addition, light-based geolocation cannot resolve latitude around the equinoxes and neither latitude nor longitude can be resolved in spatiotemporal regions of 24 h daylight [104]. Locations calculated during pre-breeding would have been largely unaffected by the equinox (Fig. S1), but the pre-laying exodus occurred when 24 h daylight extended to 64°S, so that locations of any birds foraging in coastal waters would have been unresolved. In practice, we suspect that this would not have caused a large bias because the general pattern (confirmed by GPS tracking) through to the end of incubation was for birds to forage 2° south of the outer ice edge, which in the pre-laying exodus was located between ~58–61°S (Fig. 2). During late post-brood, 24 h daylight could also have resulted in failure to resolve foraging locations far from colonies in the southern Weddell Sea (cf. Fig. S1 and Fig. 2). However, we estimated a greater late post-brood foraging range via GLS compared to early post-brood estimates via GPS, consistent with foraging range increasing over the chick-rearing period observed in other species [e.g. [105, 106], See Sect. 4.3]. For these reasons, we have greater confidence in the results from the GPS data (i.e., from the incubation, brood-guard and post-brood periods) than those resulting from GLS tracking and reiterate that the extents of foraging areas estimated using the two methods are unlikely to be directly comparable.

### Intrinsic and extrinsic effects on foraging range

Seasonal variation in snow petrel foraging ranges conformed to the pattern typically observed among pelagic seabirds, ultimately reflecting intrinsic breeding constraints [1, 3, 31, 105]. Range was greatest prior to breeding, when birds are least constrained, and during the pre-laying exodus, when they return to sea for approximately two weeks. Lowest ranges were recorded during brood-guard, when parents alternate between foraging and caring for the newly-hatched chick, in our study typically swapping every four days.

Flight speed, fasting endurance of adults and chicks, and many other traits scale allometrically in seabirds [107]. Among breeding snow petrels, trip duration is negatively associated with body size [60]. It might therefore be expected that foraging range increases with body size [3, 108]. We studied snow petrels of the ‘lesser’ form [8, 59, 109, 110], and so our results may not be generalisable to the larger form breeding elsewhere, particularly if there is size-mediated competitive exclusion [60].

Size-mediated competitive asymmetry, as well as other sexual differences, can also result in differences between male and female foraging areas and ranges [32, 33, 111–113]. Males in our study were 6–7% heavier than females, which is typical for the species [109, 114, 115]. Our sample of known-sex birds during pre-breeding and the pre-laying exodus was small and the effect of season potentially confounding (Table 1). Nonetheless, our results are consistent with a sex difference in breeding duties suggested by previous, colony-based studies [60, 116]. Among birds tracked in 2022/23 and 2023/24, males foraged around 200 km closer to the colony than females during both pre-breeding and the pre-laying exodus. We assume that this is due to an imperative for males to return earlier to the colony and invest more time in nest defence (also evinced by the greater amount of time they spent at the nest site prior to breeding) and for females to invest more time in egg production [111, 117]. In contrast, and in common with snow petrels tracked from Terre Adélie during incubation [11], we found no evidence of spatial segregation or differences in foraging range between sexes during incubation or chick rearing. This does not preclude niche partitioning between the sexes along axes of habitat (e.g., sea-ice concentration) or diet (as in [11]). Habitat partitioning could occur without broad differences in space use or foraging range as sea-ice concentration in the spring and summer in our study area is highly patchy and dynamic due to temperature, winds and currents [48, 118]. Moreover, we caution that because our sample sizes were small, we may have failed to detect subtle between-sex differences in space use.

Evidence is growing that flying seabirds track particular sea-ice habitat in time and space [40, 119], so sea-ice dynamics might be expected to affect foraging range. We found a strong positive association between the meridional location of the ice edge and foraging latitude, which was around 2° (~220 km) south of the ice edge - i.e., within the MIZ. This relationship was evident not only within but also across breeding seasons (Fig. 4). For example, just prior to colony return and during the pre-laying exodus in 2023, when sea-ice extent was at a record low [120], birds foraged closer to the colony than in previous years (Fig. 2) and in 2021/22 and 2023/24, core foraging areas during the pre-laying exodus encompassed the Maud Rise, where seasonal sea-ice thinning occurs earlier than in the surrounding pack (Fig. 1). Other GLS studies suggest that snow petrels track the ice edge over winter (c.f. Delord et al. [45], Viola et al. [121], Fetterer et al. [69]), and at-sea observations indicate that they do so at both large and fine spatiotemporal scales [41, 42, 122]. In our study, the relationship between the latitudes of snow petrels and the ice edge broke down during chick-rearing (mid-January, Fig. 4), when open water occured all the way to the coast (Fig. 1). From this time, birds increasingly moved west following the MIZ, which continues to retreat in the eastern Weddell Sea until the summer minimum occurs in February (cf. Figures 1, 2 and 3).

Given that sea ice in the vicinity of most snow petrel colonies around Antarctica retreats broadly towards colonies, we hypothesise that breeding schedules and therefore potential foraging ranges, are synchronised to sea-ice dynamics, such that chicks hatch when suitable foraging habitat is closest to the colony [123], then during the chick-rearing period, adults take advantage of the peak in secondary production that typically follows sea-ice breakup by several weeks to months in our study area [40, 119]. Matching of breeding schedules to maximise access to resources during the most energetically demanding phase may be particularly important for snow petrels because they breed at such high latitudes that their breeding season is relatively short [124]. Further investigation of the resource-tracking hypothesis should explore the effects of seasonal variation in habitat availability on habitat selection [119].

Among central-place foraging seabirds, both foraging range and distribution can be affected by density-dependent competition [35, 36]. Within stages, we did not observe large differences in foraging range among colonies, possibly because numbers of birds breeding at or near those sites were of a similar magnitude [1000s; 7, 95]. However, where we were able to compare core foraging areas among colonies, these were either partially or fully segregated prior to incubation. This is not necessarily due to competition: Segregation could have arisen from birds commuting between their colonies to the nearest available habitat patch, which during spring and early summer is usually immediately to the north. By post-brood, birds from all three study sites had partially overlapping core foraging areas, presumably because large patches of intermediate SIC occurred only near the coast, west from the prime meridian into the Weddell Sea embayment (Fig. 1). This is consistent with studies showing that sharing of space tends to occur at similar distances to colonies and when food availability is superabundant [125]. Indeed, density-dependent competitive effects in our study system may be less than at lower latitudes because the productivity of Antarctic waters during the summer is so high [126].

The other extrinsic effects evident in our data were those imposed by wind and topography on movement. It was beyond our scope to investigate these in detail, but we attempted to account for them in analysis via several assumptions. Firstly, that once at sea, commuting snow petrels avoided crossing land until they were returning to the colony. This assumption is orthodox [46] and well supported by our data. Route choice during the relatively long commute between the colony and the coast was more complex. Typically, outward commutes over the ice sheet were more westerly than the reciprocal headings of inward commutes. Other Antarctic fulmarine petrels also behave in this way, presumably to optimise movement through the strong prevailing easterly winds of the Antarctic coastal zone [88, 127]. In short, it is hypothesised that this strategy allows wind drift on the way out but compensates for it on the way back. To estimate realised foraging ranges taking into account these movement constraints, we approximated distance on overland commutes by beelines to coast crossing locations observed via GPS-tracking and assumed that these were similar for birds tracked with via GLS. Although these assumptions are expedient, further observation and analysis of the effects of wind on travel costs and route optimisation strategies [31, 128] may result in refined foraging range estimates and projections. Despite these reservations, we are confident that distances as defined in our study are a more realistic indication of the actual commuting distances than the beeline distances used in many previous studies. For example, beeline and biological distances between the colony and the location illustrated in Fig. S2 are 734 and 1065 km, respectively, illustrating that the former can greatly underestimate potential foraging range.

### Implications for interpretation of paleo-climate and occupation histories from stomach-oil deposits

**Fig. 5:**
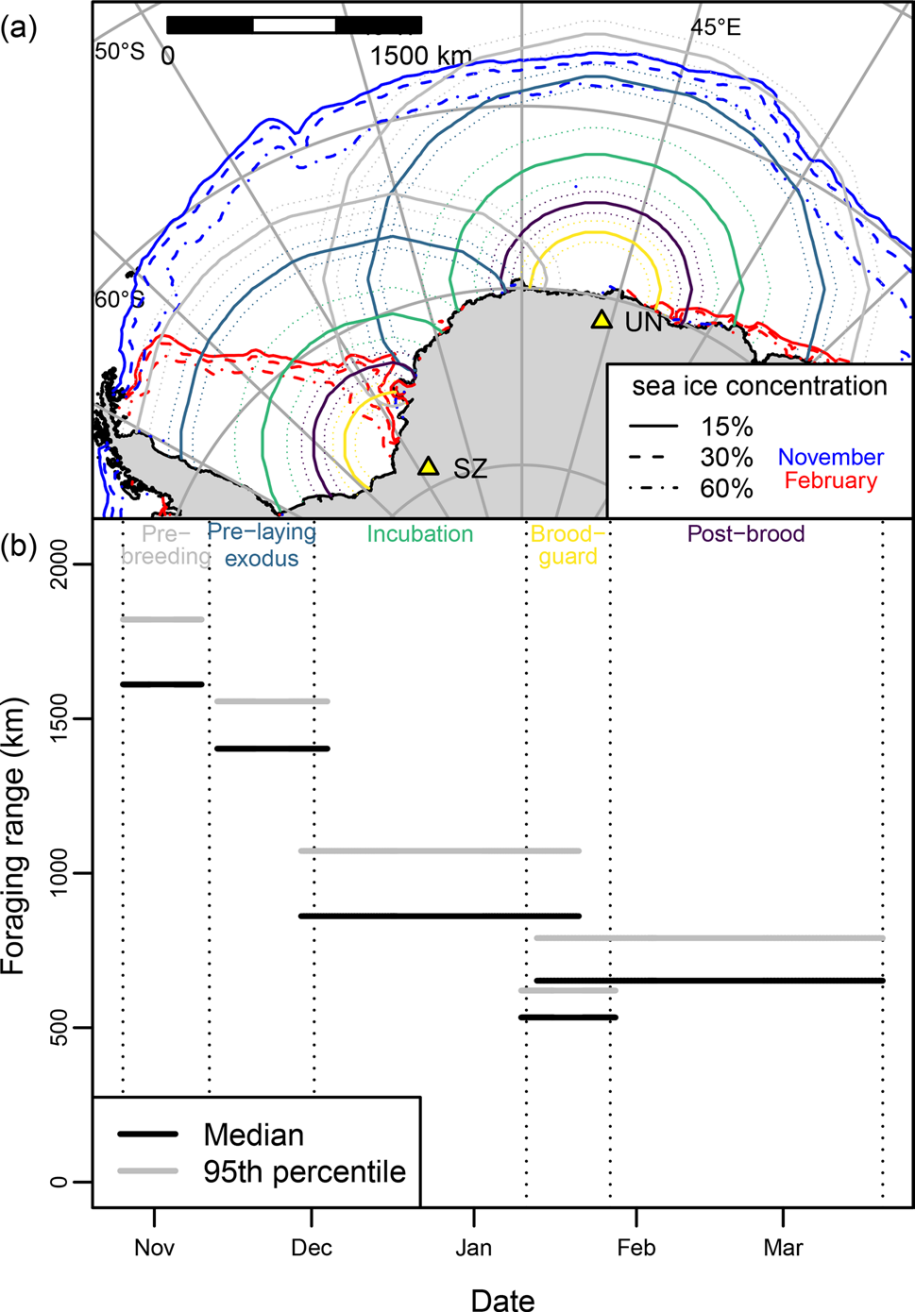
(**a**) Potential 95th percentile foraging ranges (with 95% confidence intervals) of snow petrels from Untersee Oasis (UN) and station Z.508 (SZ) during all breeding stages, plus 30 y median sea ice concentration contours. (**b**) Median and 95th percentile foraging ranges of snow petrels tracked from three colonies in Dronning Maud Land vs. breeding schedule. Confidence intervals show the 2.5 and 97.5 percentiles. Sea-ice metrics derived from national snow and ice data centre data [47]

Interpretation of palaeoclimatological and ecological proxies from stomach-oil deposits involves assumptions about where the snow petrels could have foraged [5, 6, 49]. Previously, these were based on sparse data that typically underestimated potential foraging ranges. Using our results, we have projected potential foraging ranges for all breeding sites in our study region (Supplementary Materials 2), which we hope will aid interpretation of proxies derived from stomach oil deposits. These show that areas accessible to snow petrels, and therefore over which stomach oil deposits potentially integrate sea ice and other environmental conditions, could be as large as 5 million km^2^ (Fig. 5a). However, our results also show that foraging ranges contract by a factor of two to three over the breeding cycle. Hence, it is important to consider how stomach oil deposition might vary seasonally. If adults and chicks spit oil at a similar rate throughout the breeding season, deposits would integrate paleo-environmental information over a wide range of distances and sea-ice conditions, but with a bias towards locations < 750 km from the colony (Fig. 5b). Alternatively, oil deposition could occur in one or more short pulses, the most likely being between first return to the colony and early incubation, when competition to establish (or reestablish) and defend nest sites and mates is intense [8, 116]. In this scenario, accumulated stomach oil would reflect environmental conditions prior to laying, when potential foraging ranges exceed 1500 km and sea-ice extent is near its seasonal maximum [129]. Moreover, accumulated oil could originate predominantly from males because they are more active in nest acquisition and defence and take the first incubation shift [116, 130]. This is relevant because, as discussed above, males forage closer to the colony than females during pre-breeding and the pre-laying exodus. Deposition could also vary due to predation pressure from skuas, which in Terre Adélie peaks in early post-brood [18]. On balance, we suggest deposition probably peaks early in the breeding season, during courtship and subsequent defence of nests from non-breeders, declining thereafter. Deposits therefore probably predominantly reflect sea-ice conditions during spring and early summer. These assumptions could be tested by directly measuring seasonal variation in deposition rates. The interpretation of dietary signals as indicators of sea ice conditions (e.g [5, 6],) will also need to consider that foraging effort is unlikely to be evenly distributed throughout the potential range, but is probably patchily distributed due to seasonally changing sea ice conditions, prey availability and possibly competition.

**Fig. 6:**
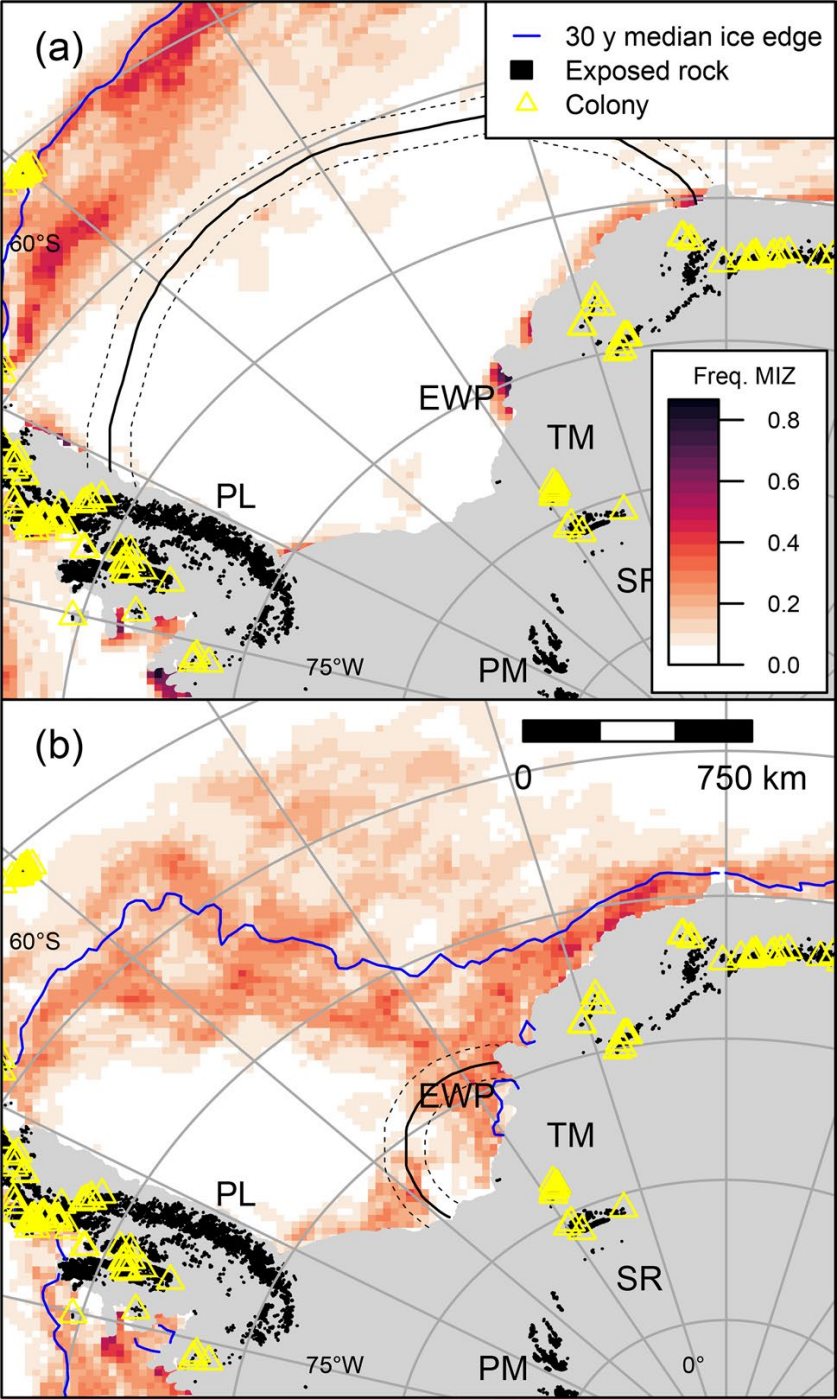
Potential foraging ranges of birds from station Z.508 (solid yellow triangle), Antarctica during (**a**) the pre-laying exodus and (**b**) brood-guard, plus the 30-y mean frequency of occurrence of the marginal ice zone (MIZ - sea-ice concentrations between 30 and 60%). Station Z.508 is the site furthest from the coast in the Weddell sea region for which there is robust evidence of breeding [7, 132]. EWP, Eastern Weddell Polynya; PL, palmer Land; PM, Pensacola Mountains; SR, Shackleton Range; TM, Theron Mountains. Sea ice metrics derived from National Snow and Ice Data Centre data [47]

Assuming that snow petrel breeding distribution is limited by both distance to the MIZ and availability of exposed rock for nesting [10], our results can provide new insights into past, present and future nest site occupation patterns [22, 131]. Today, exposed nunataks occur throughout the coastal zone of the Weddell Sea (Fig. 6), but nesting apparently does not occur in eastern Palmer Land or the Pensacola Mountains, offshore of which there is persistent, closed (i.e. high SIC), multiyear pack ice in the western Weddell Sea during summer, and coastal polynyas are few and small [129]. Snow petrels nest at low densities in the Theron Mountains and possibly the Shackleton Range. If birds from these colonies had similar foraging ranges to those we observed, the MIZ of the outer ice edge would be inaccessible to them early in breeding season (Fig. 6a). Presumably they instead forage in or at the margins of coastal polynyas [44], such as the Eastern Weddell Polynya, which persistently occurs adjacent to the Brunt Ice Shelf [133, 134]. During chick-rearing, intermediate sea ice cover occurs over wider but spatiotemporally patchier areas (Fig. 6b) so accessibility of suitable foraging habitat integrated across the entire breeding season may be limiting.

During the last glacial stage, both breeding habitat and access to suitable foraging habitat were thought to be more limited than today [22, 131]. However, snow petrels occupied some colonies in DML throughout this period [20, 21, 50], despite the summer ice edge being > 2,000 km north [26, 135, 136]. Ultimately, the maximum viable foraging range of snow petrels under these circumstances remains undetermined. However, if it were assumed (with the caveats in Sect. 4.2) that they were similar to those in our study, the ice edge would have been inaccessible from these colonies during brood-guard, supporting the hypothesis that foraging was instead sustained by polynyas [5, 6, 44]. These could have occurred not only along the coast [137] but also offshore, e.g., over the Maud Rise [138], which is just within the observed brood-guard range of existing colonies in the west of our study area (cf. Figs. 1a and 6a). Another possibility is that birds foraged in leads, which persistently occur where tidal currents and bathymetry interact, such as at the shelf break [139].

During the transitions between glacial and interglacial stages, which occurred on timescales of tens of thousands of years, there may have been differences in the periods when foraging habitat and breeding habitat became accessible. For example, retreat of the summer sea ice from its glacial maximum began ~23 ka (thousands of years before the present) in the Atlantic sector of the Southern Ocean [135], but ice-sheet mass loss and warming associated with deglaciation started later, from 20 ka [26, 140]. Under this scenario, foraging habitat may have been available but potential nest sites may still have been ice-covered, especially given that ice sheet thinning occurs later in our study region, mainly from ~10–8 ka [141]. Alternatively, foraging habitat may have become accessible sometime after breeding habitat was exposed [142]. This would have introduced latency between breeding habitat exposure and its occupation by snow petrels, which should be borne in mind when inferring ice sheet thinning from stomach oil deposits [22, 50].

Currently, the Southern Ocean is thought to be undergoing a regime shift from a positive to negative trend in summer sea-ice cover [96], exacerbated by anthropogenic climate change [143]. In the future, it is likely the retreat of ice shelves and terrestrial ice sheet margins caused by anthropogenic climate change will impact snow petrels. In particular, foraging habitat (i.e., intermediate sea-ice cover) will become less extensive and seasonally persistent in some areas, whereas breeding habitat (exposed rock) may become more extensive. Hence, snow petrels may be expected to colonise breeding habitat currently at the margins of accessibility to the MIZ and abandon areas where it becomes inaccessible. In our study area, this would result in a southwest shift in breeding distribution, perhaps with areas such as the Pensacola Mountains being colonised for the first time. However, changes in sea-ice phenology [144, 145] and the wider marine ecosystem [39, 146] and ultimately the loss of summer sea ice [25], may have unsustainable impacts on snow petrels.

## Conclusions

Previously, lack of data on the foraging ranges of breeding snow petrels led to uncertainty in the interpretation of proxies of paleoclimate derived from ancient stomach oil deposits accumulated around snow petrel nests [5, 6, 49] as well as the environmental factors limiting their current and past breeding distribution [29, 30]. Our study shows that not only is foraging range greater than hitherto assumed, but it is also very variable seasonally. Median foraging range was greatest (1400 km) at the start of the breeding cycle during the pre-laying exodus, and least (530 km) during the brood-guard period in the middle of the cycle. A strong correlation between foraging latitude and the latitude of the outer ice edge, plus the known affinity of snow petrels for intermediate sea conditions [9–11], suggests that the decrease in foraging range is due to the seasonal retreat of the pack ice. Indeed, we hypothesise that the breeding schedule of snow petrels is adapted so that brood-guard, when birds are most temporally constrained by chick rearing duties, occurs when the Marginal Ice Zone occurs closest to the colony. Based on our results, and assuming that snow petrels predominantly deposit stomach oil early in the breeding season, when nest defence is most intense, paleoclimate proxies derived from these deposits likely reflect sea ice conditions during spring, when ice cover is near its seasonal maximum. In addition, although tracking of birds from other colonies is required to confirm whether the foraging ranges we observed are widely representative, our results support the hypothesis that breeding at some sites bordering the Weddell Sea may only be feasible due to coastal polynyas or areas of recurrent leads providing foraging habitat south of the outer Marginal Ice Zone [44].

## Electronic supplementary material

Below is the link to the electronic supplementary material.

Supplementary material 1

Supplementary material 2

## Data Availability

The tracking datasets analysed during the current study are available in the BirdLife Seabird Tracking Database https://data.seabirdtracking.org/dataset ID tbc.

## Abbreviations

ASPA: Antarctic Specially Protected Area
BC: Bhattacharyya’s Coefficient
DML: Dronning Maud Land
GLM: Generalised Linear Model
GLMM: Generalised Linear Mixed-effects Model
GLS: Global Location Sensors
HROI: Home Range Overlap Index
MIZ: Marginal Ice Zone
SIC: Sea Ice Concentration
