# Supplementary material for: Variability in foraging ranges of snow petrels and implications for breeding distribution and use of stomach-oil deposits as proxies for paleoclimate
Source: Mov Ecol. 2025 Nov 20;13:83. doi: 10.1186/s40462-025-00609-7 (PMC12636188; doi:10.1186/s40462-025-00609-7)
Supplement: Supplementary file 1 — Supplementary material 1 [file 40462_2025_609_MOESM1_ESM.docx]

**Supplementary Material 1 – Supplementary Methods, Figures and Tables**

**Variability in foraging ranges of snow petrels and implications for breeding distribution and use of stomach-oil deposits as proxies for paleoclimate**

Ewan D. Wakefield^1*^, Erin L. McClymont^1^, Sébastian Descamps^2^, W. James Grecian^1^, A. Rus Hoelzel^3^, Eleanor M. Honan^1^, Anna S. Rix^1^, Henri Robert^4^, Vegard Sandøy Bråthen^5^, Richard A. Phillips^3^

*Corresponding author [ewan.wakefield@durham.ac.uk](mailto:ewan.wakefield@durham.ac.uk)

1. Department of Geography, Durham University, Lower Mountjoy, South Road, Durham, DH1 3LE, UK.

2. Norwegian Polar Institute, Fram Centre, 9296 Tromsø, Norway.

3. Department of Biosciences, Durham University, Durham, DH1 3LE, UK.

4. International Polar Foundation, Rue des vétérinaires, 42b/1, 1070 Brussels, Belgium.

5. Norwegian Institute for Nature Research, P.O. Box 5685 Torgarden, 7485 Trondheim, Norway.

6. British Antarctic Survey, Natural Environment Research Council, Cambridge, CB3 0ET, United Kingdom.

**S1. Supplementary Methods**

**S1.1. GLS data processing**

GPS tracking showed that during incubation birds travelled at least as far north as 59°S (see Results). During midsummer, sea ice recedes to the coast of Dronning Maud Land, above the Antarctic circle making light-based geolocation impractical (Fig. S1). Geolocation is again practicable in late summer, from the date when sunset/sunrise again occurs at latitudes at which snow petrels forage (approx. February 5th), to two weeks before the autumn equinox (March 16th), when geolocation is impractical due to equal daylength across latitudes.


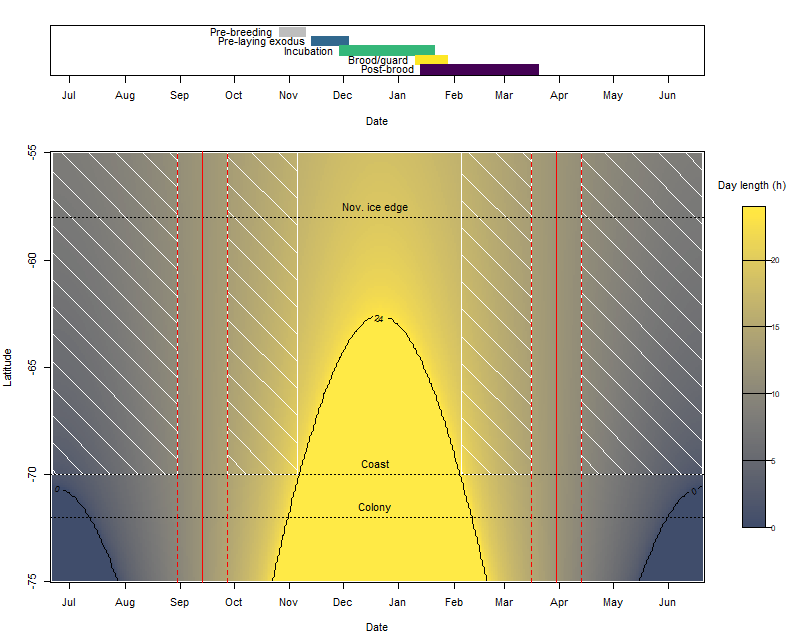


**Fig. S1.** Breeding phenology of snow petrels (top panel) and variation in day length with latitude and time of year and its effect on the tractability of light-based geolocation via the threshold method (bottom panel). Sunrise and sunset are defined here as occurring when the sun is 4.1° below the horizon, the mean values used in our analysis. Red lines show the equinoxes, +/- 14 days; yellow the zone in which 24 h daylight occurs; and the black horizontal dashed lines the approximate latitudes of the November ice edge, the coast of Dronning Maud Land and the study colonies. Areas hatched white are the spatiotemporal regions within which bird locations occurring as far south as the central Dronning Maud Land coast could theoretically be resolved.

Following the protocol developed by Bråthen et al. [[1](#_ENREF_1)] to analyse geolocation data for mid to high latitude seabirds [[2](#_ENREF_2)], we used the threshold method to estimate bird locations from the timing of local twilights recorded by the GLS loggers [[3](#_ENREF_3), [4](#_ENREF_4)]. We identified twilights by assuming that these occurred when light levels crossed a threshold of 11 lux. Following [Lisovski and Hahn](#_ENREF_72) [[5](#_ENREF_5)], we fitted polynomial regression models to twilight times, identifying and removing outliers. We assumed that twilight occurred when the sun was at a fixed angle below the horizon but that this could vary between loggers and years [[6](#_ENREF_6)]. Using methods adapted from Hanssen et al. [[7](#_ENREF_7)] and van Bemmelen et al. [[8](#_ENREF_8)], we determined the most appropriate solar angle for each logger/year by visual inspection of plots of latitude vs. time, assuming that the appropriate angle minimised discrepancies in latitudes on either side of the equinoxes. Following this calibration procedure, the mean solar angle was 4.1 ± 0.4° below the horizon. We then estimated locations following Lisovski and Hahn [[5](#_ENREF_5)] and applied a multi-stage filtering process to reduce errors [[1](#_ENREF_1)]. Firstly, we removed locations occurring within ±14 days of the equinoxes when day length varies little with latitude. Due to the solar angle being negative, apparent equinox dates are shifted towards the winter solstice [[9](#_ENREF_9)], so periods removed were centred on average on October 6 (range October 4 - 8) and March 14 (range March 10 - 18). Next, we applied the distanceFilter function in the GeoLight package [[5](#_ENREF_5)], removing locations resulting in speeds >40 km/h over 12 h, and then the sdafilter function from the argosfilter package [[10](#_ENREF_10)], removing unrealistic ‘spikes’ – characterised by steps greater than 480 and 960 km combined with turning angles of less than 15 and 35°, respectively. We then removed locations outside the known range of snow petrels (85 – 49° south) and applied a double smoothing procedure following Hanssen et al. [[7](#_ENREF_7)].

**S1.2. GPS data processing**

We split GPS tracks into foraging trips defining these as excursions beginning and ending ≤1 km from the colony, reaching >50 km from the colony and comprising at least three locations. Due to the power management algorithm used by the GPS loggers, which balances location attempts with battery voltage, and variation in the latter due to shading while birds were on the nest and charging while they were in the open, most locations were recorded at either 30- or 60-minute intervals (see Results). To avoid temporal sampling imbalance biasing our estimates of foraging range, we regularised location estimates in time. To do so, we used the aniMotum package [[11](#_ENREF_11)] to fit a correlated random walk model to each trip and predict locations at 30 minute intervals. We specified the ‘optim’ optimizer, spike removal distances of 5 and 10 km and bivariate normal GPS location errors of ± 20 m, using defaults for all other settings. We used one-step-ahead prediction residuals to check goodness of fit [[12](#_ENREF_12)].

**S1.3. Molecular sex determination**

We determined sex via standard molecular methods, using primers 2550F and 2718R previously employed for snow petrels [[13](#_ENREF_13), [14](#_ENREF_14)]. We isolated total genomic DNA from feather tips using the protocol and reagents of the E.Z.N.A. tissue DNA kit (Omega Bio-tek; Norcross, GA, USA), additionally adding DTT (Dithiothreitol) with the proteinase K digested samples overnight in a 55 ºC water bath. We eluted DNA twice using 50 µL of low EDTA, TE buffer (Thermo Scientific Chemicals; Waltham, MA, USA). We then amplified DNA using Platinum II Taq Hot-Start DNA polymerase (Invitrogen) for 50 cycles with annealing at 50 °C for 25 seconds and extension at 68 °C for 15 seconds and examined PCR products using 1.2% agarose gels. We ran all PCR amplifications and gels with a known female snow petrel and a negative PCR control (water). We designated males as birds that produced the CHD1Z band only (~650 bp) and females as those that produced the CHD1W band (~450 bp) additionally or alone. We reran samples that initially produced no or very faint bands using differing amounts of DNA until sex was unambiguously established.

**S2. Supplementary Tables**

**Table S1.** Initial and estimated parameter values in Hidden Markov Models of behavioural state of snow petrels GPS-tracked from three colonies in Dronning Maud Land.

|  | | Parameter | |  |  | State |  |
| --- | --- | --- | --- | --- | --- | --- | --- |
|  | |  |  | Resting | Foraging | Travelling |  |
| Step length (m/s) | | mean | initial range | 0.2-1.0 | 3.0-5.0 | 9.0-15.0 |  |
|  |  |  | estimate | 0.6 | 4.2 | 8.5 |  |
|  |  | sd | initial range | 0.2-1.0 | 3.0-5.0 | 9.0-15.0 |  |
|  |  |  | estimate | 0.6 | 2.3 | 3.2 |  |
| Angle (degrees) | | mean | initial | 0.0 | 0.0 | 0.0 |  |
|  |  |  | estimate | 0.0 | 0.0 | 0.0 |  |
|  |  | concentration | initial range | 0.5-0.8 | 0.5-5.0 | 15.0-25.0 |  |
|  |  |  | estimate | 0.7 | 1.4 | 10.5 |  |

**Table S2.** Mean masses (g) ± sd (range) of snow petrels sampled at the three study colonies in Dronning Maud Land.

| Colony | Females | Males |
| --- | --- | --- |
| Jutulsessen | 232 ± 24 (205 -275; n = 15) | 242 ± 27 (210 -315; n = 16) |
| Svarthamaren | 258 ± 32 (220 -340; n = 15) | 287 ± 40 (230 -370; n = 25) |
| Utsteinen | 257 ± 31 (205 -325; n = 17) | 264 ± 19 (230 -300; n = 14) |

**Table S3.** Linear model of the mass of snow petrels weighed in colonies in Dronning Maud Land vs. sex and colony (models containing interactions between sex and colony (ΔAIC = 1.3), just sex (ΔAIC = 4.6) or just colony (ΔAIC = 19.7) were less parsimonious).

| Covariate | Mass (g) | SE | *t* | *p* |
| --- | --- | --- | --- | --- |
| Jutulsessen, female (intercept) | 228.9 | 6.4 | 35.57 | <0.001 |
| Svarthamaren | 37.5 | 7.5 | 5.03 | <0.001 |
| Utsteinen | 24.3 | 7.9 | 3.08 | <0.001 |
| Male | 16.0 | 6.2 | 2.56 | 0.010 |

**Table S4.** Linear models of median foraging range $d_{50}$ of snow petrels tracked via geolocation from three colonies in Dronning Maud Land during pre-breeding (the ten days prior to colony arrival) and the pre-laying exodus vs. breeding season (stages modelled separately).

| Stage | Breeding season | $d_{50}$(km) | SE | *t* | *p* |
| --- | --- | --- | --- | --- | --- |
| Pre-breeding | 2020/21 (intercept) | 1754 | 104 | 16.92 | <0.001 |
|  | 2021/22 | 177 | 147 | 1.20 | 0.235 |
|  | 2022/23 | -103 | 120 | -0.86 | 0.395 |
|  | 2023/24 | -515 | 115 | -4.48 | <0.001 |
| Pre-laying exodus | 2020/21 (intercept) | 1670 | 85 | 19.62 | <0.001 |
|  | 2021/22 | -354 | 120 | -2.94 | 0.005 |
|  | 2022/23 | -242 | 94 | -2.56 | 0.013 |
|  | 2023/24 | -411 | 94 | -4.35 | <0.001 |

**S3. Supplemenatary Figures**


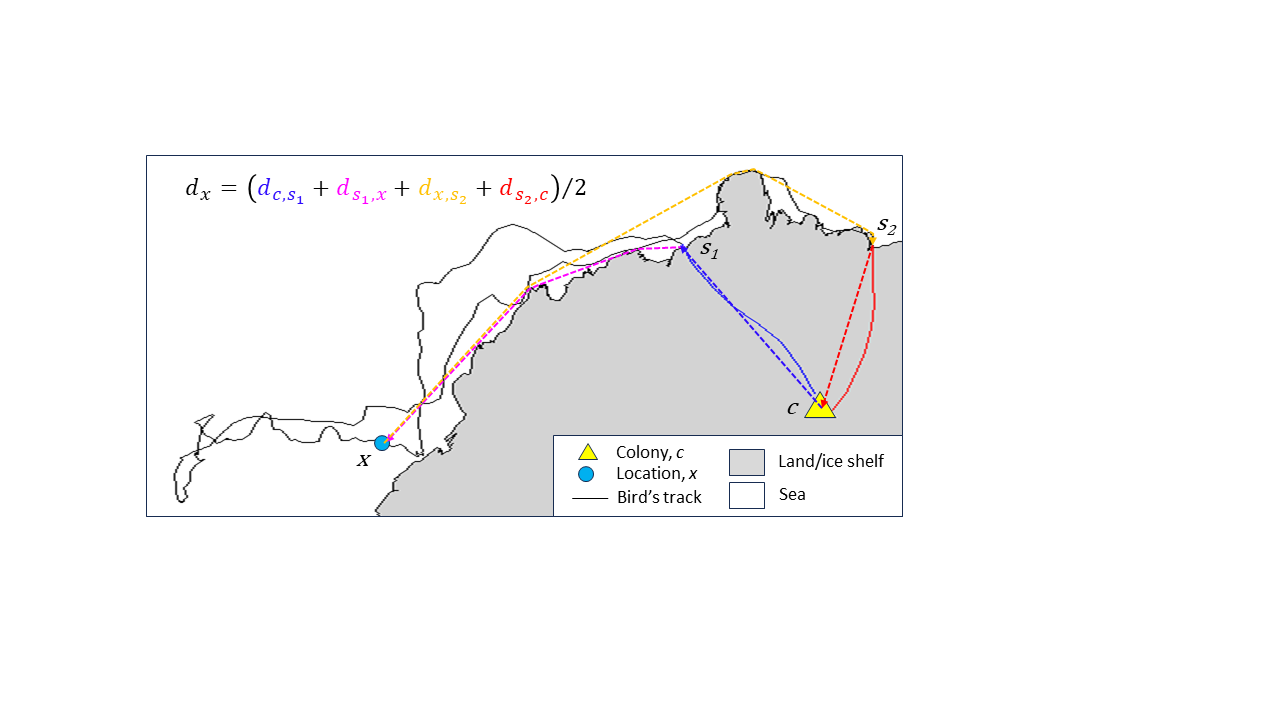


**Fig. S2.** Example of track of a foraging trip made by a snow petrel tracked via GPS from Svarthamaren, Dronning Maud Land during post-brood, illustrating how we defined biological distance, $d$ between the colony, *c* and an example location, *x* and passing thought the outward and inward coast crossing locations, $s_{1}$ and$s_{2}$.


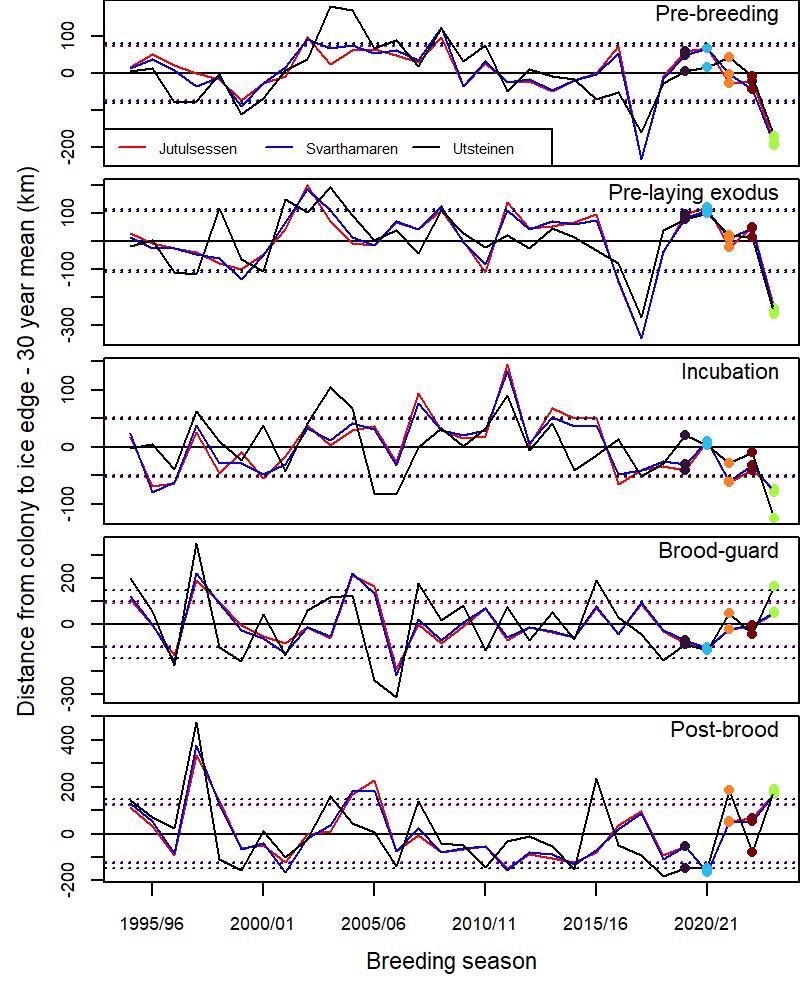


**Fig. S3.** Mean biological distance to the ice edge from the three study colonies during breeding stages relative to the 30-year climatic mean. Dashed lines show the standard deviation of the climatic mean for each breeding stage and colony. Points highlight study seasons, with colours corresponding to Figs. 1-3.


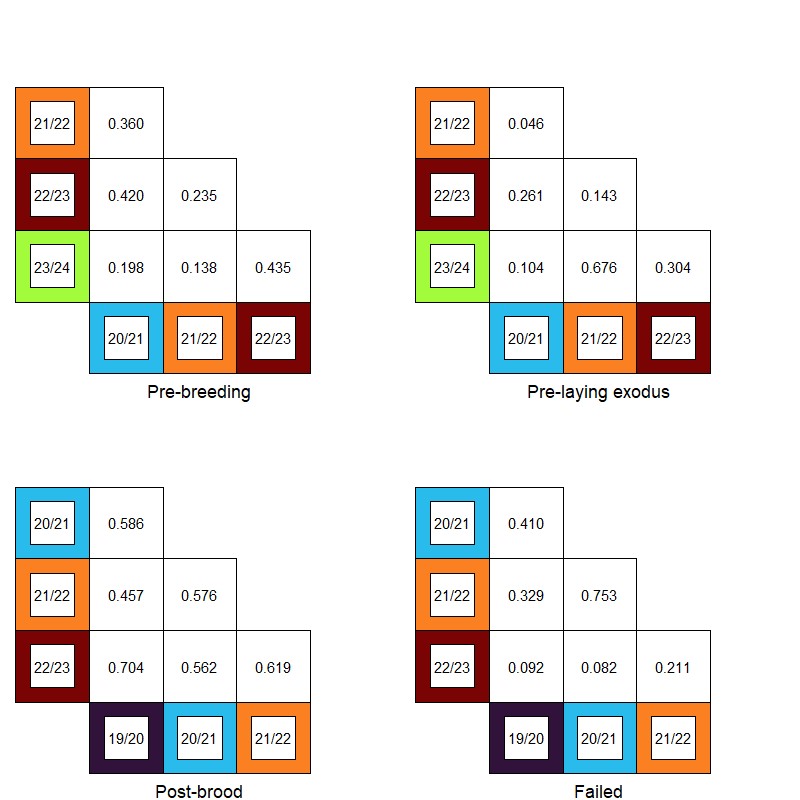


**Fig. S4.** Home Range Overlap Index between core foraging areas of birds GLS-tracked from Svarthamaren, Dronning Maud Land during five breeding seasons (colours correspond to Figs. 1 and 2).


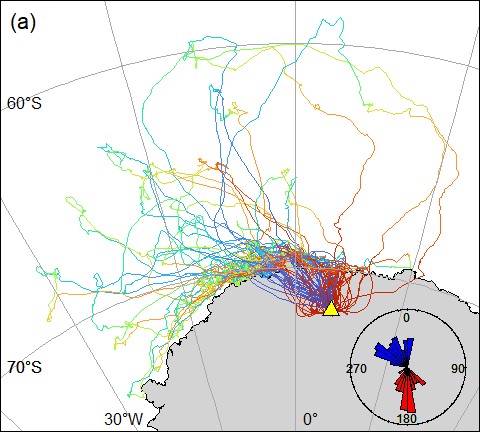


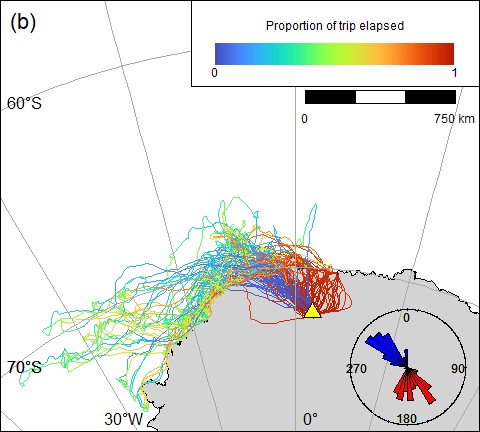


**Fig. S5.** Foraging trips made by snow petrels GPS-tracked from Svarthamaren (incubation, brood-guard and post-brood, 2022/23) and Jutulsessen (post-brood, 2024), Dronning Maud Land coloured by proportion of the trip elapsed. Insets show the distributions of mean bird headings on outward (blue) and inward (red) overland commuting legs. Yellow triangles show study colonies.


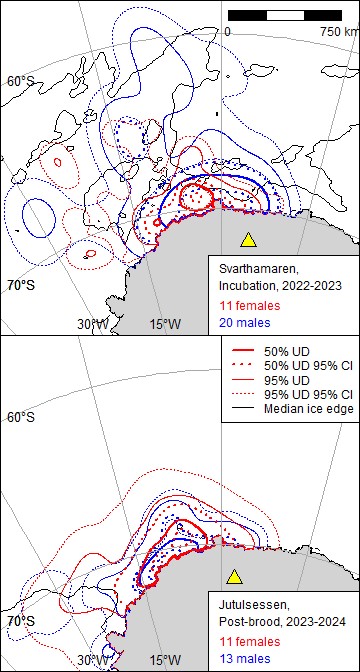


**Fig. S6.** Utilisation distributions (UDs) of female and male snow petrels GPS-tracked from Svathamaren during incubation and Jutulsessen, Dronning Maud Land during post-brood chick rearing. Yellow triangles indicate study colonies.

**S4. Supplemenatary References**

1. Bråthen V, Moe B, Amélineau F, Ekker M, Helgason H, Johansen M, et al.: An automated procedure (v2.0) to obtain positions from light-level geolocators in large- scale tracking of seabirds A method description for the SEATRACK project. In. Trondheim, Norway: NINA Report 1893; 2021: 52.

2. Fauchald P, Tarroux A, Amélineau F, Bråthen VS, Descamps S, Ekker M, et al. Year-round distribution of Northeast Atlantic seabird populations: applications for population management and marine spatial planning. Marine Ecology Progress Series. 2021;676:255-76.

3. Hill RD, Braun MJ. Geolocation by Light Level. In: Sibert JR, Nielsen JL, editors. Electronic Tagging and Tracking in Marine Fisheries: Proceedings of the Symposium on Tagging and Tracking Marine Fish with Electronic Devices, February 7–11, 2000, East-West Center, University of Hawaii. Dordrecht: Springer Netherlands; 2001. p. 315-30.

4. Ekstrom PA. An advance in geolocation by light. Memoirs of National Institute of Polar Research Special Issue. 2004;58:210-26.

5. Lisovski S, Hahn S. GeoLight-processing and analysing light-based geolocator data in R. Methods in Ecology and Evolution. 2012;3(6):1055-9; doi: 10.1111/j.2041-210X.2012.00248.x.

6. Lisovski S, Hewson CM, Klaassen RHG, Korner-Nievergelt F, Kristensen MW, Hahn S. Geolocation by light: accuracy and precision affected by environmental factors. Methods in Ecology and Evolution. 2012;3(3):603-12; doi: 10.1111/j.2041-210X.2012.00185.x.

7. Hanssen SA, Gabrielsen GW, Bustnes JO, Bråthen VS, Skottene E, Fenstad AA, et al. Migration strategies of common eiders from Svalbard: implications for bilateral conservation management. Polar Biology. 2016;39(11):2179-88; doi: 10.1007/s00300-016-1908-z.

8. van Bemmelen RSA, Kolbeinsson Y, Ramos R, Gilg O, Alves JA, Smith M, et al. A Migratory Divide Among Red-Necked Phalaropes in the Western Palearctic Reveals Contrasting Migration and Wintering Movement Strategies. Frontiers in Ecology and Evolution. 2019;7; doi: 10.3389/fevo.2019.00086.

9. Frederiksen M, Moe B, Daunt F, Phillips RA, Barrett RT, Bogdanova MI, et al. Multicolony tracking reveals the winter distribution of a pelagic seabird on an ocean basin scale. Diversity and Distributions. 2012;18(6):530–42; doi: 10.1111/j.1472-4642.2011.00864.x.

10. Freitas C, Lydersen C, Fedak MA, Kovacs KM. A simple new algorithm to filter marine mammal Argos locations. Marine Mammal Science. 2008;24(2):315-25; doi: <https://doi.org/10.1111/j.1748-7692.2007.00180.x>.

11. Jonsen ID, Grecian WJ, Phillips L, Carroll G, McMahon C, Harcourt RG, et al. aniMotum, an R package for animal movement data: Rapid quality control, behavioural estimation and simulation. Methods in Ecology and Evolution. 2023;14(3):806-16; doi: <https://doi.org/10.1111/2041-210X.14060>.

12. Thygesen UH, Albertsen CM, Berg CW, Kristensen K, Nielsen A. Validation of ecological state space models using the Laplace approximation. Environmental and Ecological Statistics. 2017;24(2):317-39; doi: 10.1007/s10651-017-0372-4.

13. Barbraud C, Delord K, Kato A, Bustamante P, Cherel Y. Sexual segregation in a highly pagophilic and sexually dimorphic marine predator. Peer Community Journal. 2021;1; doi: 10.24072/pcjournal.75.

14. Fridolfsson A-K, Ellegren H. A Simple and Universal Method for Molecular Sexing of Non-Ratite Birds. Journal of Avian Biology. 1999;30(1):116-21.

15. van Etten J: gdistance: distances and routes on geographical grids. R package version 1.6.4. <https://AgrDataSci.github.io/gdistance/>. 2023.
