# Supplementary material for: Variability in foraging ranges of snow petrels and implications for breeding distribution and use of stomach-oil deposits as proxies for paleoclimate
Source: Mov Ecol. 2025 Nov 20;13:83. doi: 10.1186/s40462-025-00609-7 (PMC12636188; doi:10.1186/s40462-025-00609-7)
Supplement: Supplementary file 2 — Supplementary material 2 [file 40462_2025_609_MOESM2_ESM.zip › Read Me.docx]

**Supplementary Material 2 – Snow petrel foraging ranges projected for colonies on the Antarctic continent between 30ºW and 25ºE**

**Variability in foraging ranges of snow petrels and implications for breeding distribution and use of stomach-oil deposits as proxies for paleoclimate**

Ewan D. Wakefield^1*^, Erin L. McClymont^1^, Sébastien Descamps^2^, W. James Grecian^1^, A. Rus Hoelzel^3^, Eleanor M. Honan^1^, Anna S. Rix^3^, Henri Robert^4^, Vegard Sandøy Bråthen^5^, Richard A. Phillips^6^

*Corresponding author [ewan.wakefield@durham.ac.uk](mailto:ewan.wakefield@durham.ac.uk)

1. Department of Geography, Durham University, Lower Mountjoy, South Road, Durham, DH1 3LE, UK.

2. Norwegian Polar Institute, Fram Centre, 9296 Tromsø, Norway.

3. Department of Biosciences, Durham University, Durham, DH1 3LE, UK.

4. International Polar Foundation, Rue des vétérinaires, 42b/1, 1070 Brussels, Belgium.

5. Norwegian Institute for Nature Research, P.O. Box 5685 Torgarden, 7485 Trondheim, Norway.

6. British Antarctic Survey, Natural Environment Research Council, Cambridge, CB3 0ET, United Kingdom.

**Data description**

The file meta.csv contains the IDs, names and locations of all known snow petrel colonies on the Antarctic continent between 30° W and 25° E. These data were extracted from an Antarctic-wide colony database collated by Francis et al. [1, 2]

The directory **/shape** files contains one shape file for each colony comprising colony biological distance contours corresponding to the mean median, 95^th^ percentage and maximum foraging ranges for each breeding stage and their 95% confidence intervals. The fields are as follows:

| Field | Definition | Levels |
| --- | --- | --- |
| percentile | The foraging range summary statistic | 50 = 50^th^ percentile (median)  95 = 95^th^ percentile  100 = 100^th^ percentile (maximum) |
| type | contour type | mu = mean of the percentile  lc = lower 95^th^ confidence interval of the mean  uc = upper 95^th^ confidence interval of the mean |
| stage | Breeding stage | BG = brood-guard  PB = post-brood  INC = incubation  PLE = pre-laying exodus  PPL = pre-breeding |

The directory **/plots** files contains one plot for each colony with three panels illustrating the projected mean median (top panel), 95^th^ percentile (middle panel) and maximum (bottom panel) foraging range.

**References**

1. Francis J, Wakefield E, Jamieson S, McClymont EL, Southwell C, Emmerson L, et al. Global breeding distribution of the snow petrel (1843-2020) (Version 1.0) [Data set]. NERC EDS UK Polar Data Centre. . 2024. <https://doi.org/10.5285/3155805f-6d8b-4a27-8dbf-91f2c10a4ba7>.

2. Francis J, Wakefield E, Jamieson SSR, Phillips RA, Hodgson DA, Southwell C, et al. A circumpolar review of the breeding distribution and habitat use of the snow petrel (Pagodroma nivea), the world’s most southerly breeding vertebrate. Polar Biology. 2024;48(1):9; doi: <https://doi.org/10.1007/s00300-024-03336-8>.
