# Supplementary figures and images for: Variability in foraging ranges of snow petrels and implications for breeding distribution and use of stomach-oil deposits as proxies for paleoclimate
Source: Mov Ecol. 2025 Nov 20;13:83. doi: 10.1186/s40462-025-00609-7 (PMC12636188; doi:10.1186/s40462-025-00609-7)

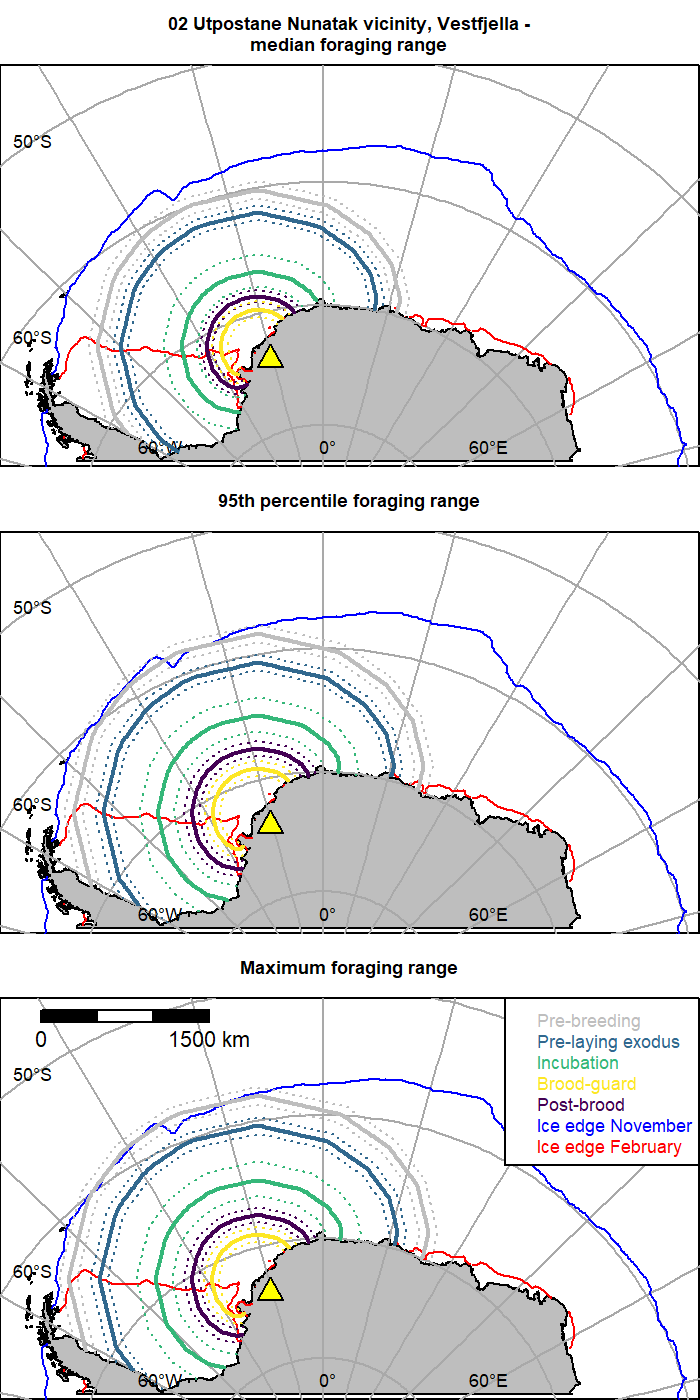

Supplement: Supplementary file 2 — Supplementary material 2 [file 40462_2025_609_MOESM2_ESM.zip › plots/bd_plot_02.png]

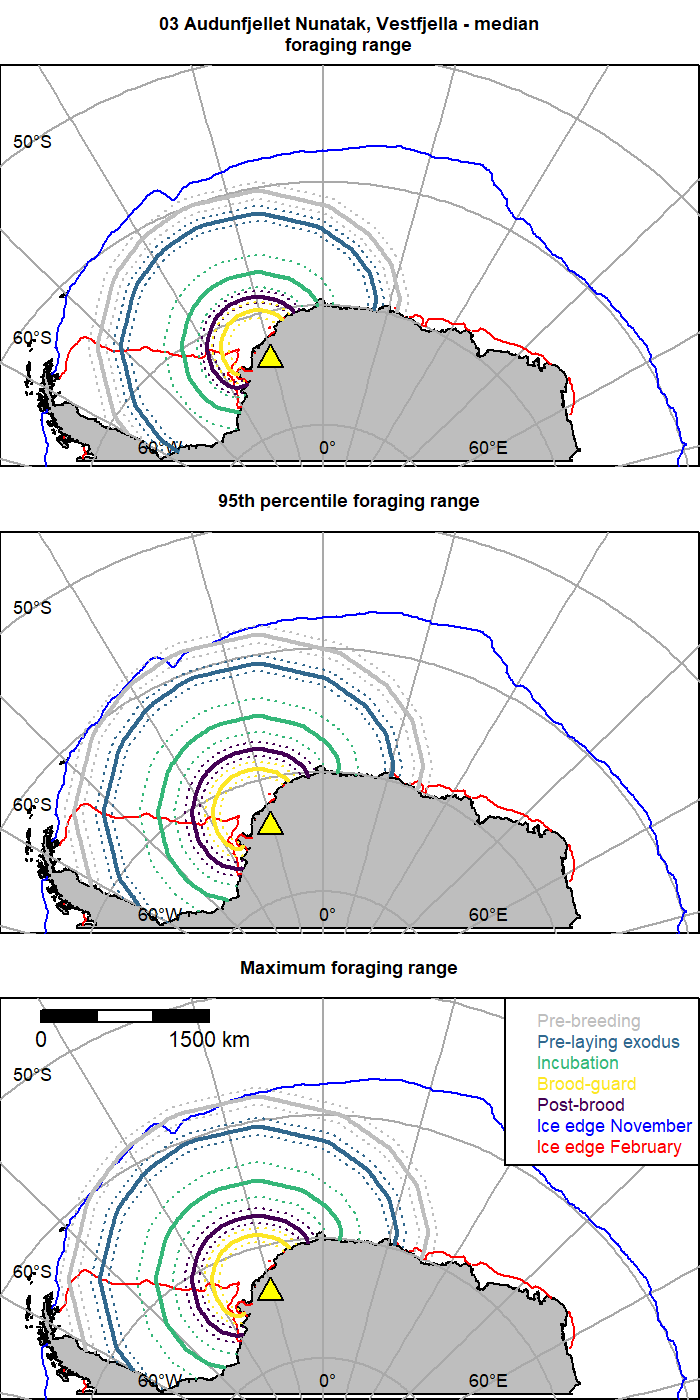

Supplement: Supplementary file 2 — Supplementary material 2 [file 40462_2025_609_MOESM2_ESM.zip › plots/bd_plot_03.png]

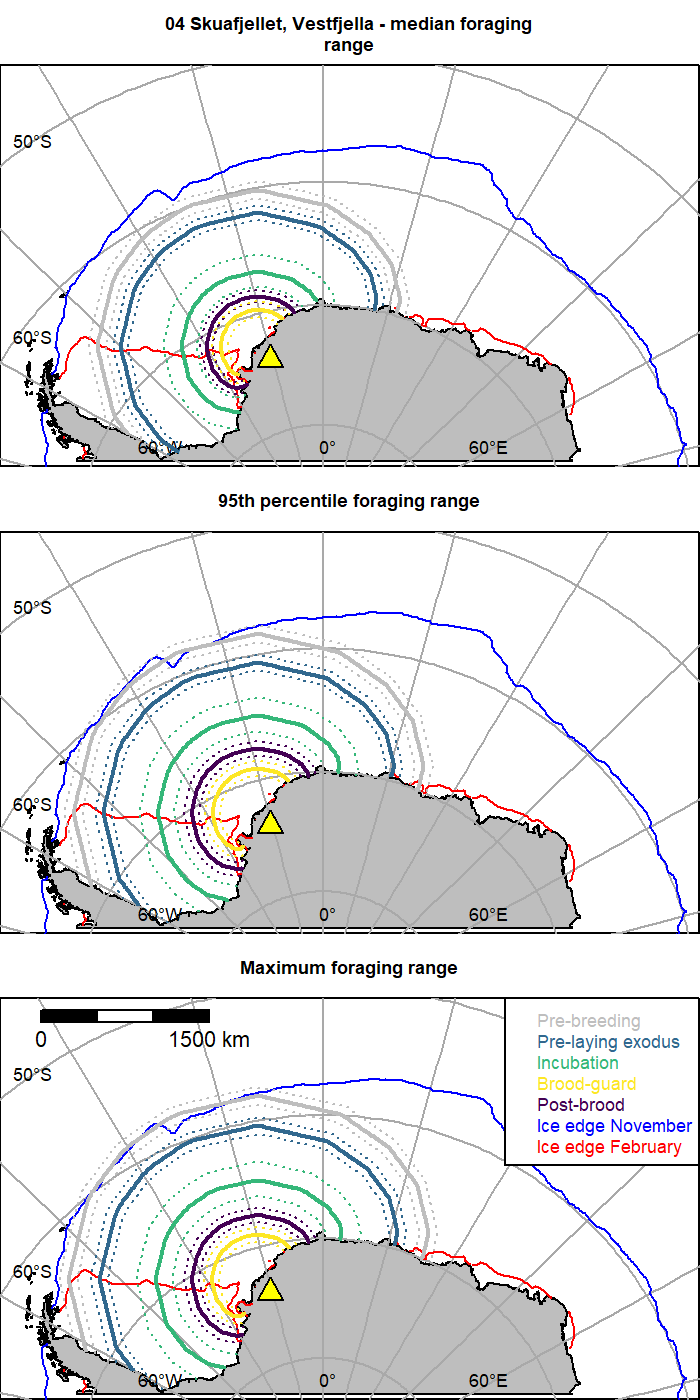

Supplement: Supplementary file 2 — Supplementary material 2 [file 40462_2025_609_MOESM2_ESM.zip › plots/bd_plot_04.png]

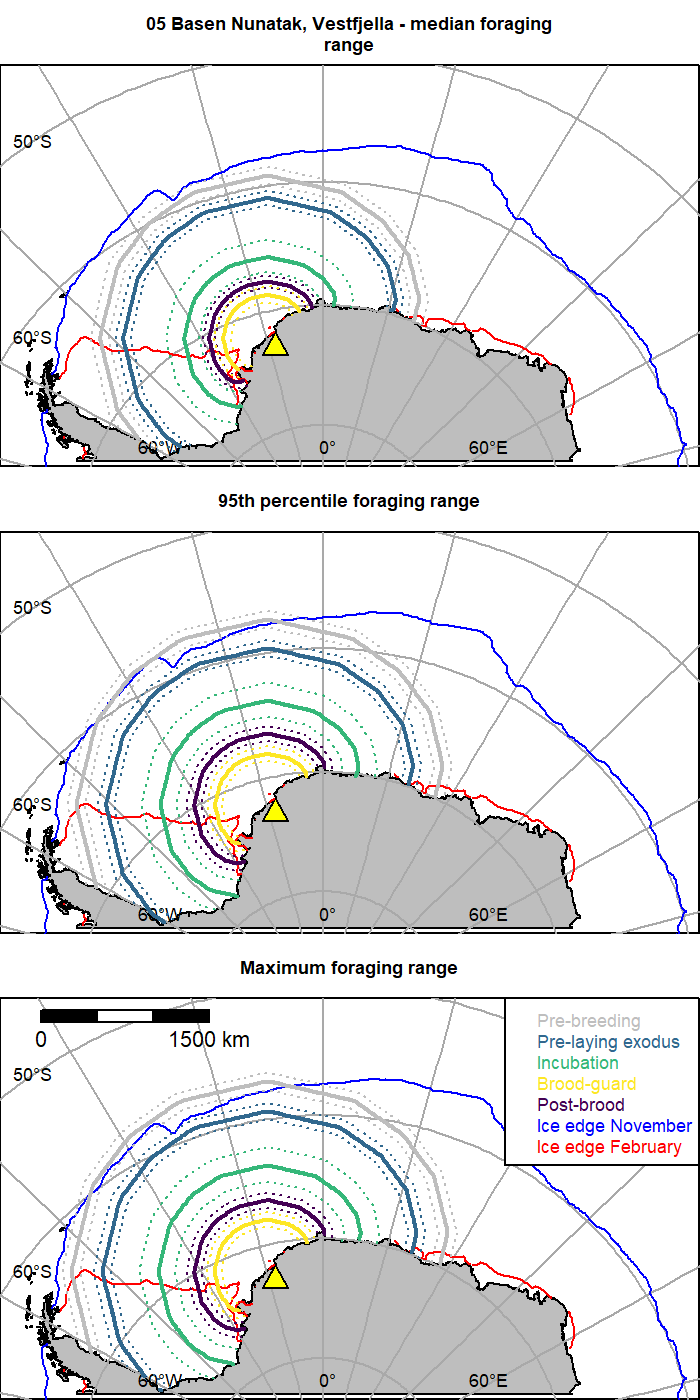

Supplement: Supplementary file 2 — Supplementary material 2 [file 40462_2025_609_MOESM2_ESM.zip › plots/bd_plot_05.png]

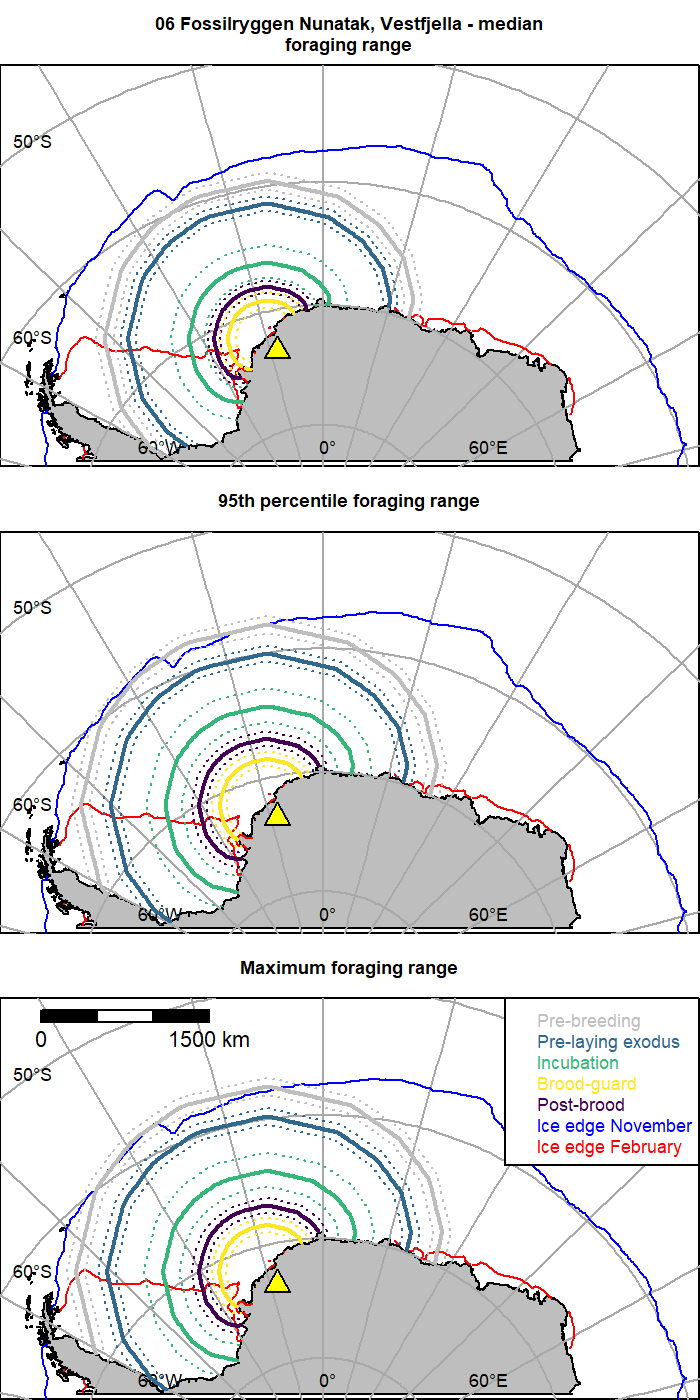

Supplement: Supplementary file 2 — Supplementary material 2 [file 40462_2025_609_MOESM2_ESM.zip › plots/bd_plot_06.png]

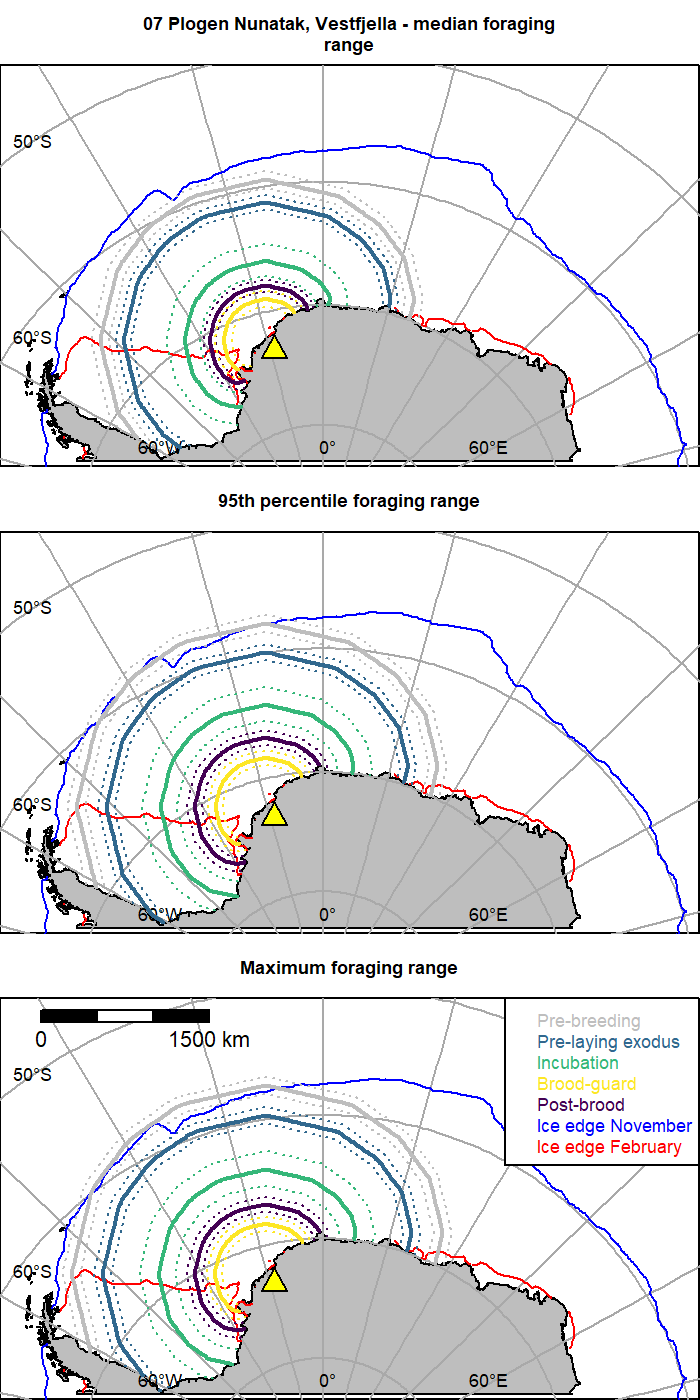

Supplement: Supplementary file 2 — Supplementary material 2 [file 40462_2025_609_MOESM2_ESM.zip › plots/bd_plot_07.png]

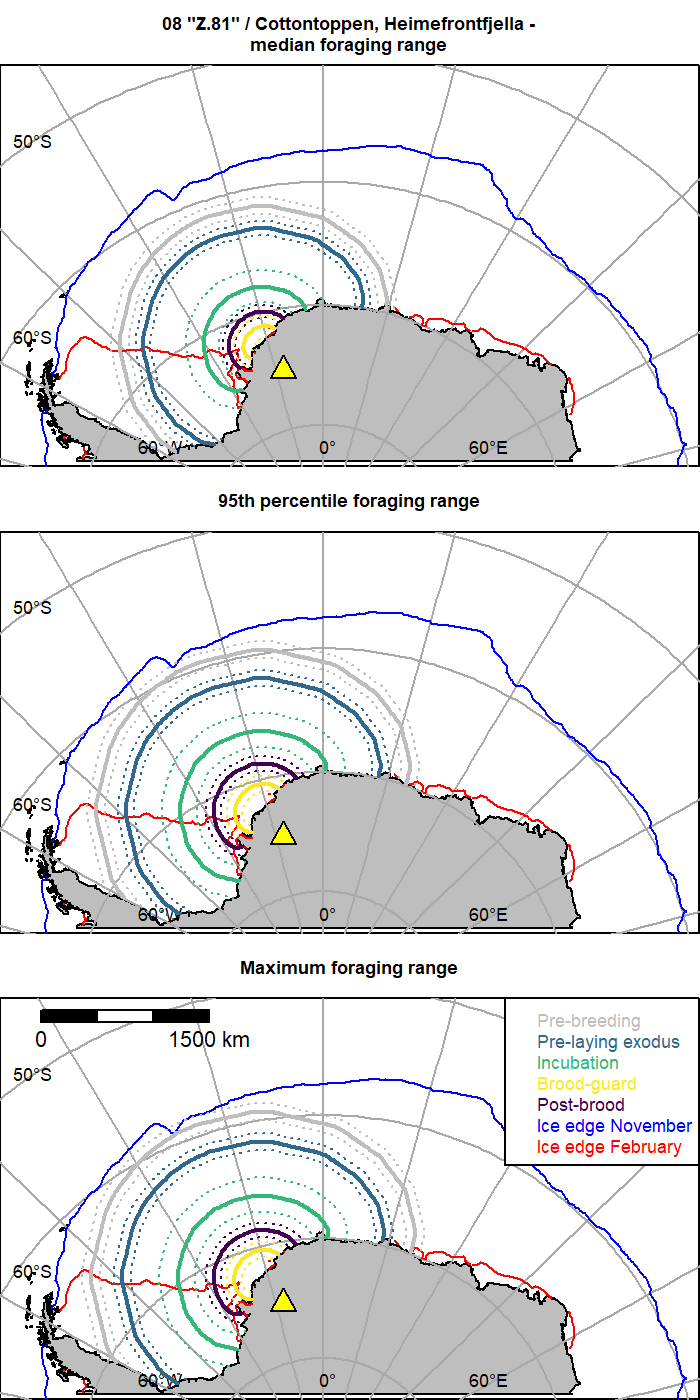

Supplement: Supplementary file 2 — Supplementary material 2 [file 40462_2025_609_MOESM2_ESM.zip › plots/bd_plot_08.png]

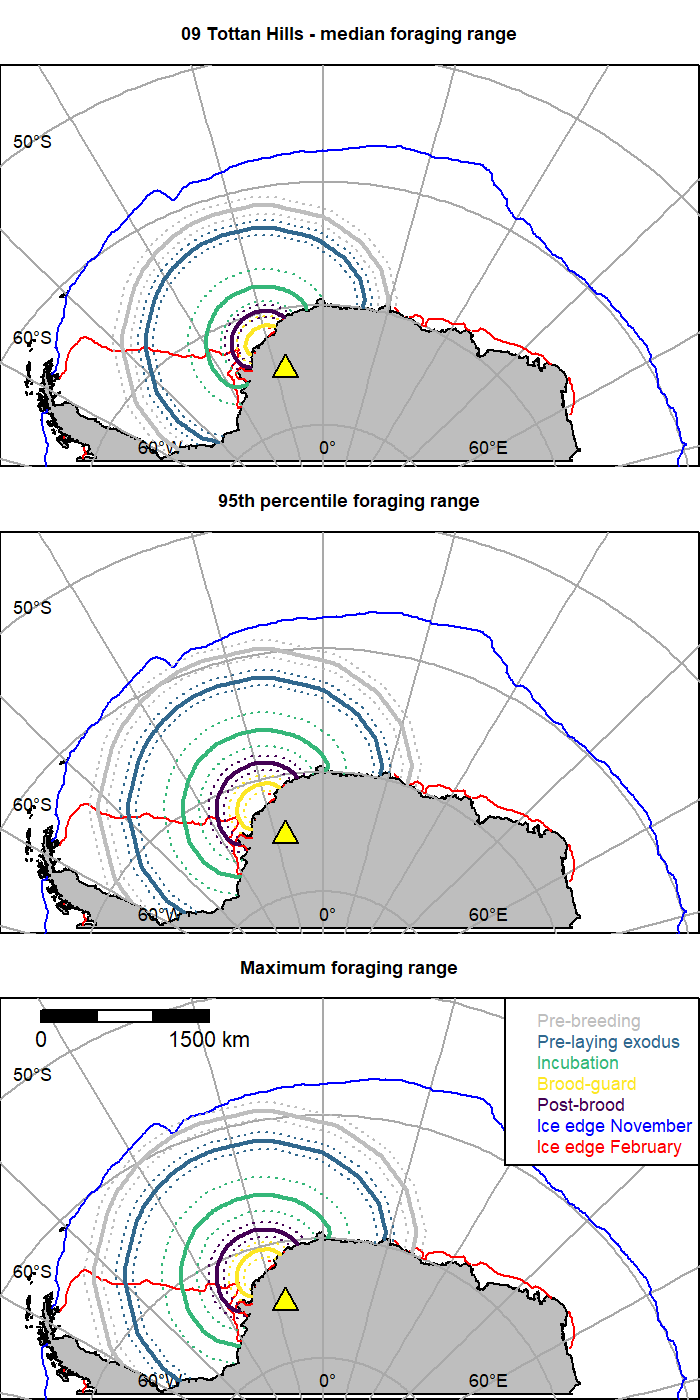

Supplement: Supplementary file 2 — Supplementary material 2 [file 40462_2025_609_MOESM2_ESM.zip › plots/bd_plot_09.png]

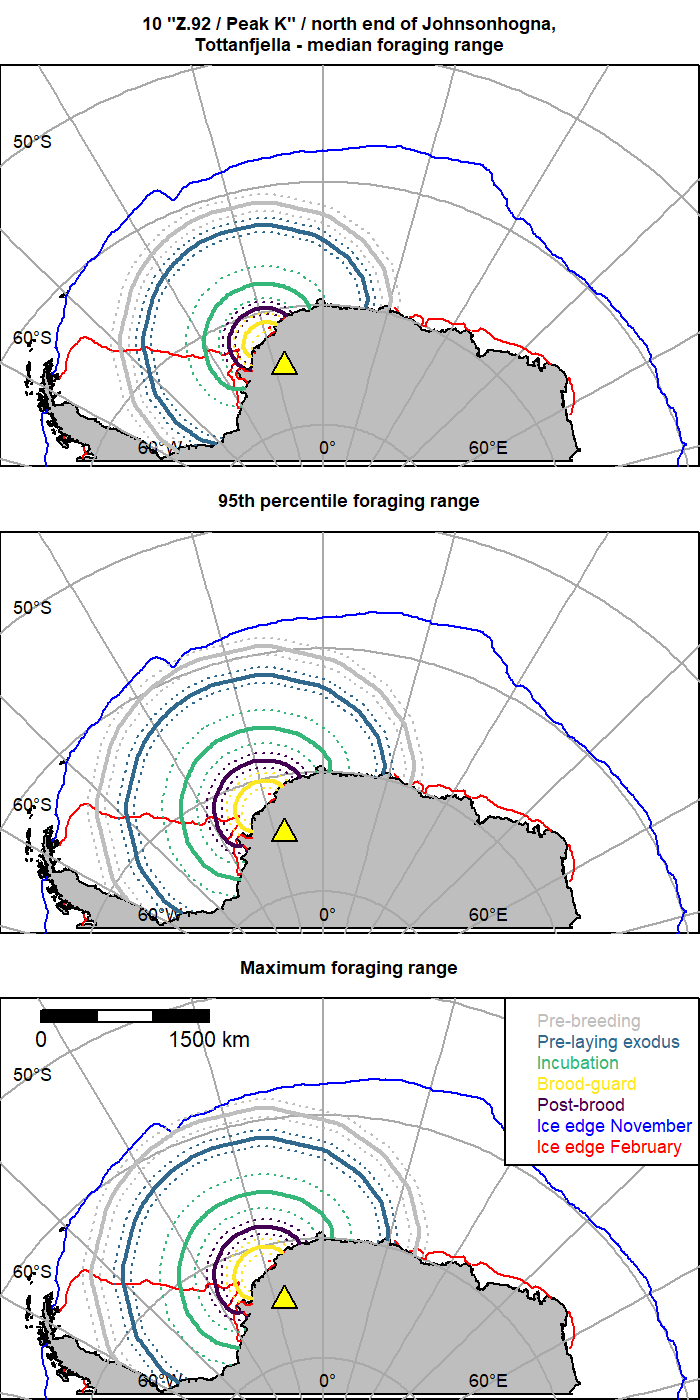

Supplement: Supplementary file 2 — Supplementary material 2 [file 40462_2025_609_MOESM2_ESM.zip › plots/bd_plot_10.png]

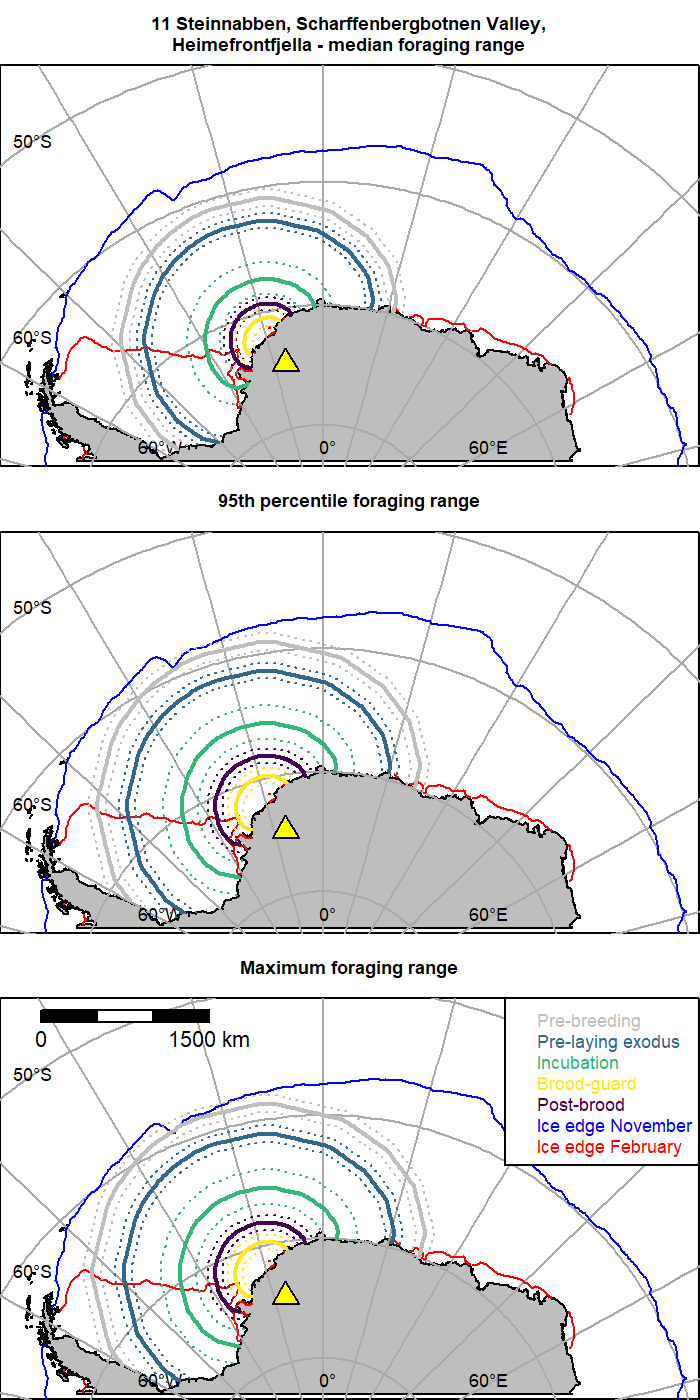

Supplement: Supplementary file 2 — Supplementary material 2 [file 40462_2025_609_MOESM2_ESM.zip › plots/bd_plot_11.png]

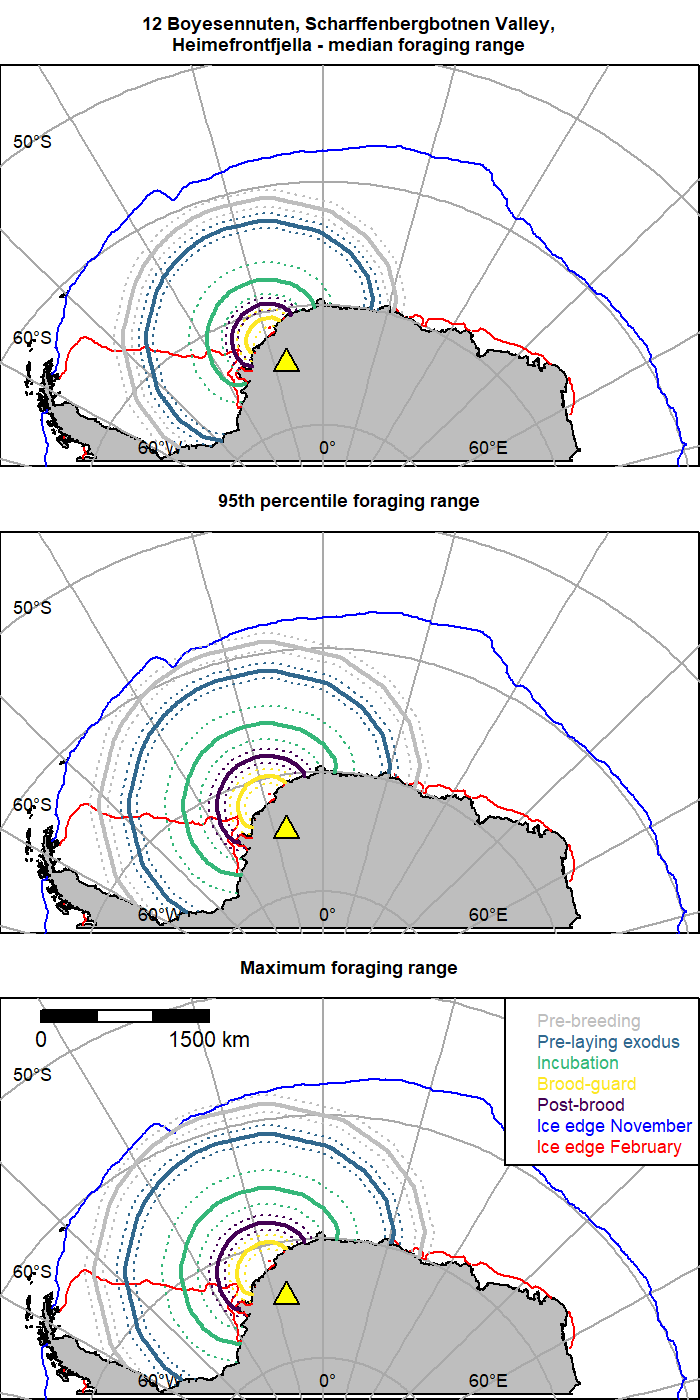

Supplement: Supplementary file 2 — Supplementary material 2 [file 40462_2025_609_MOESM2_ESM.zip › plots/bd_plot_12.png]

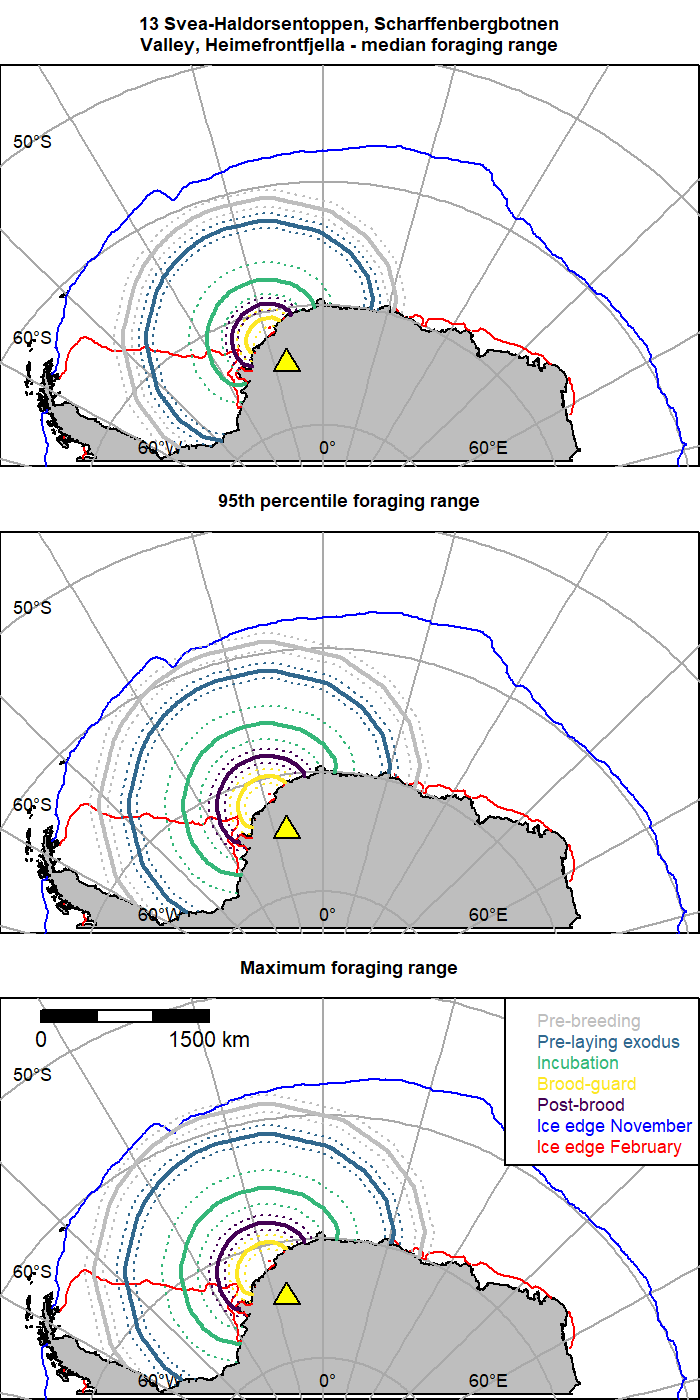

Supplement: Supplementary file 2 — Supplementary material 2 [file 40462_2025_609_MOESM2_ESM.zip › plots/bd_plot_13.png]

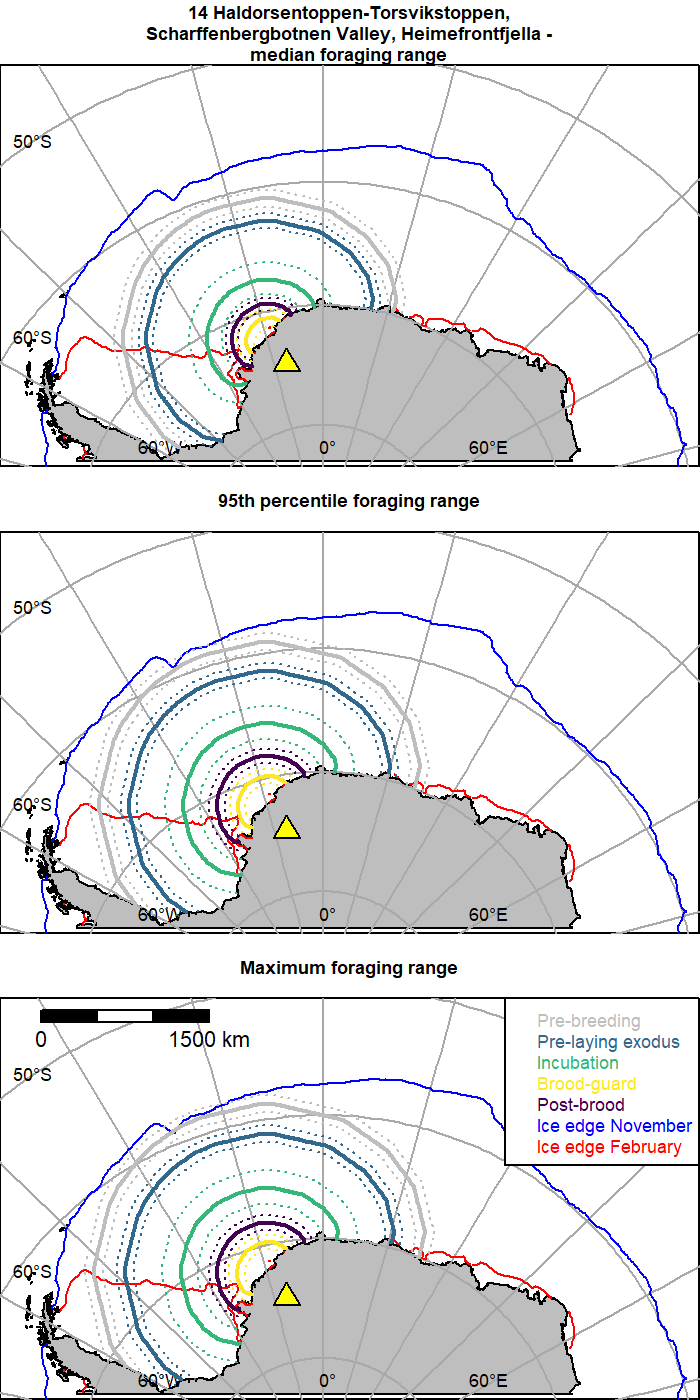

Supplement: Supplementary file 2 — Supplementary material 2 [file 40462_2025_609_MOESM2_ESM.zip › plots/bd_plot_14.png]

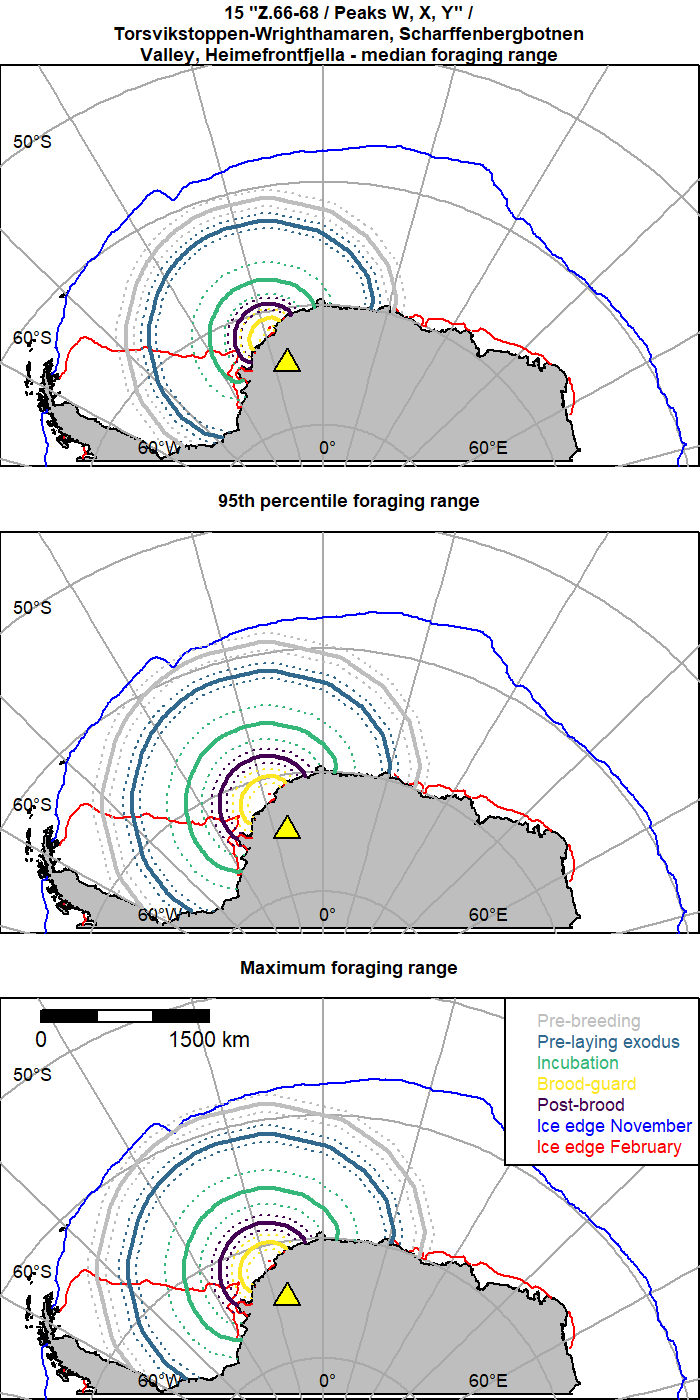

Supplement: Supplementary file 2 — Supplementary material 2 [file 40462_2025_609_MOESM2_ESM.zip › plots/bd_plot_15.png]

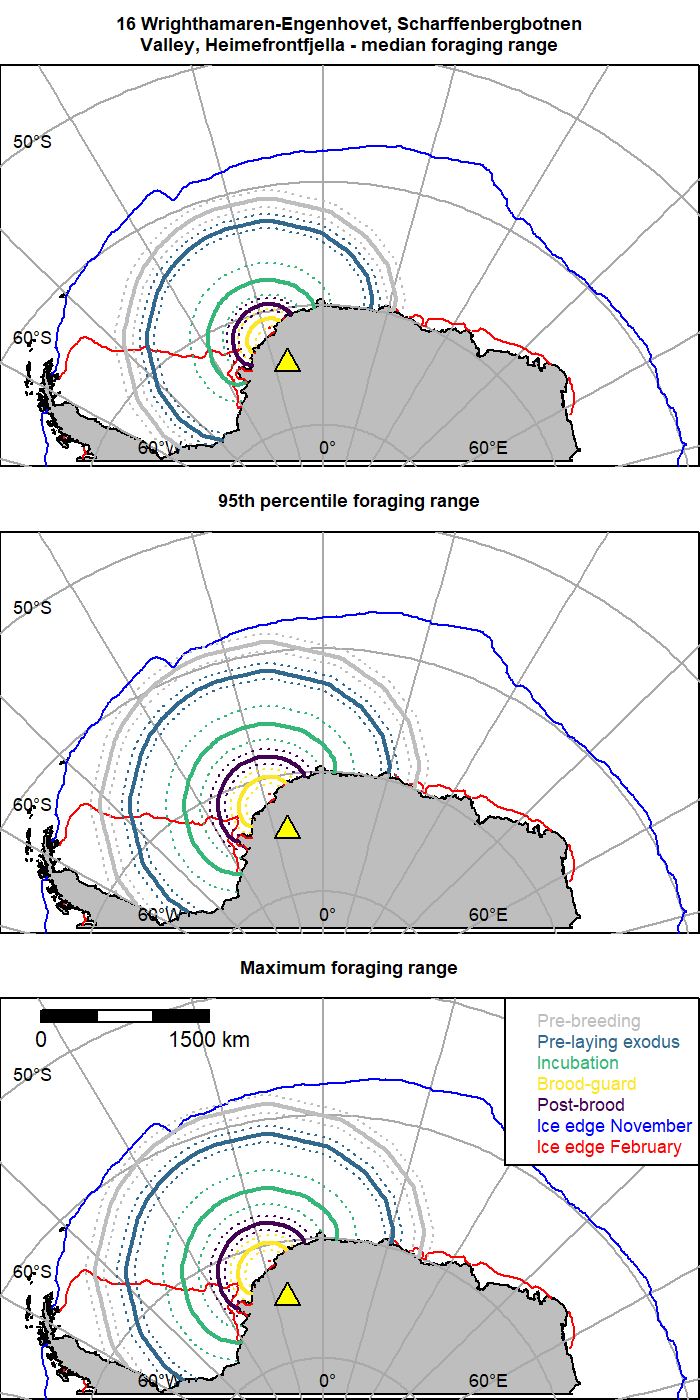

Supplement: Supplementary file 2 — Supplementary material 2 [file 40462_2025_609_MOESM2_ESM.zip › plots/bd_plot_16.png]

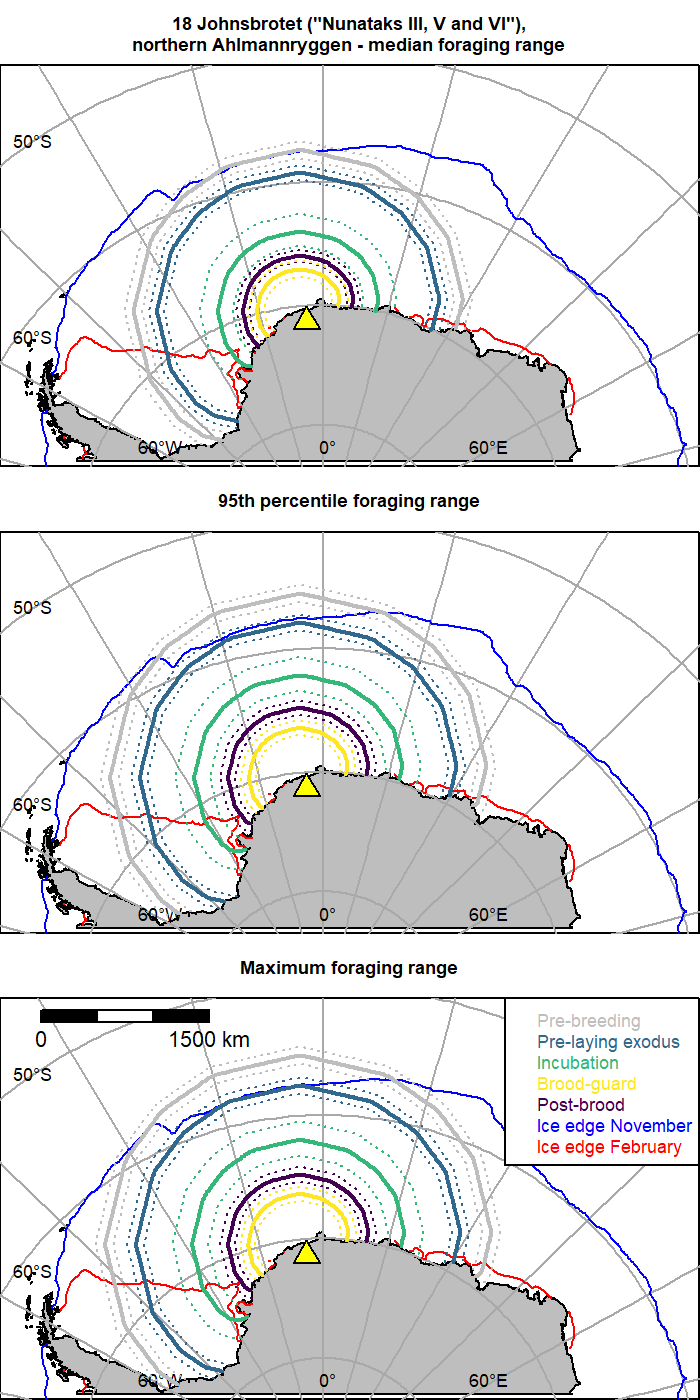

Supplement: Supplementary file 2 — Supplementary material 2 [file 40462_2025_609_MOESM2_ESM.zip › plots/bd_plot_18.png]

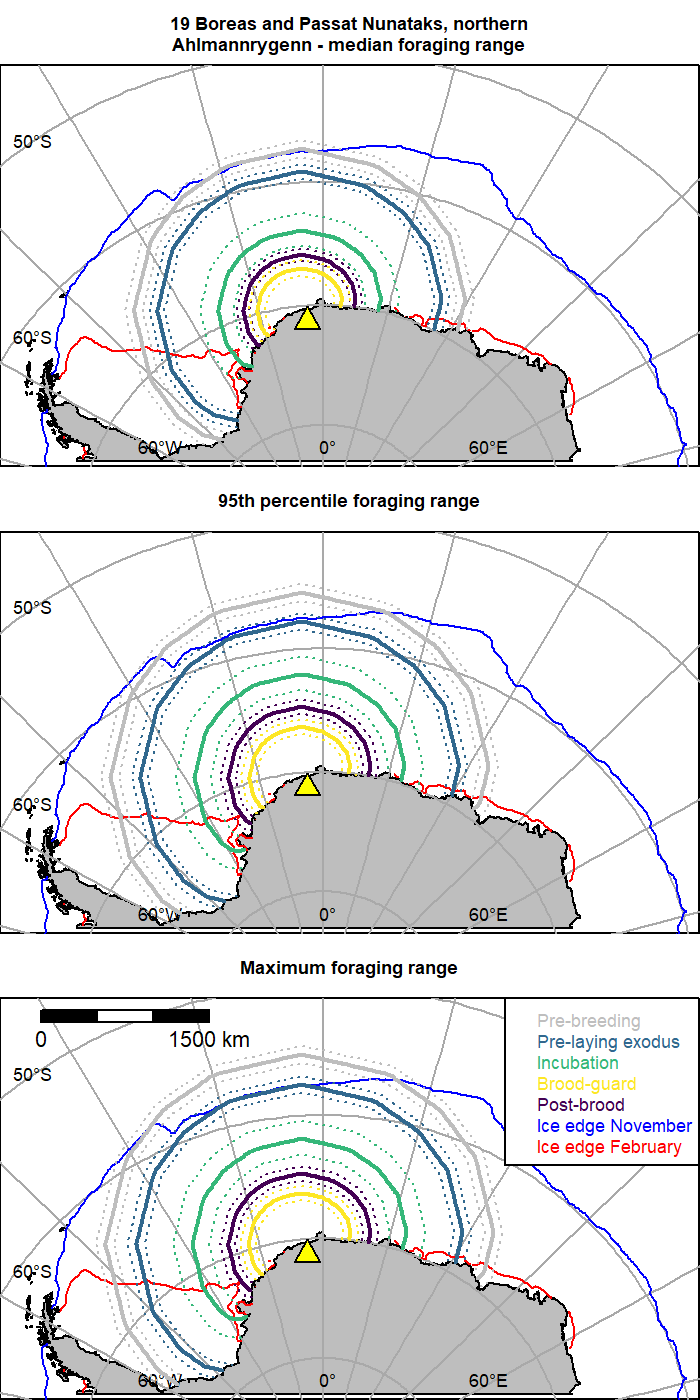

Supplement: Supplementary file 2 — Supplementary material 2 [file 40462_2025_609_MOESM2_ESM.zip › plots/bd_plot_19.png]

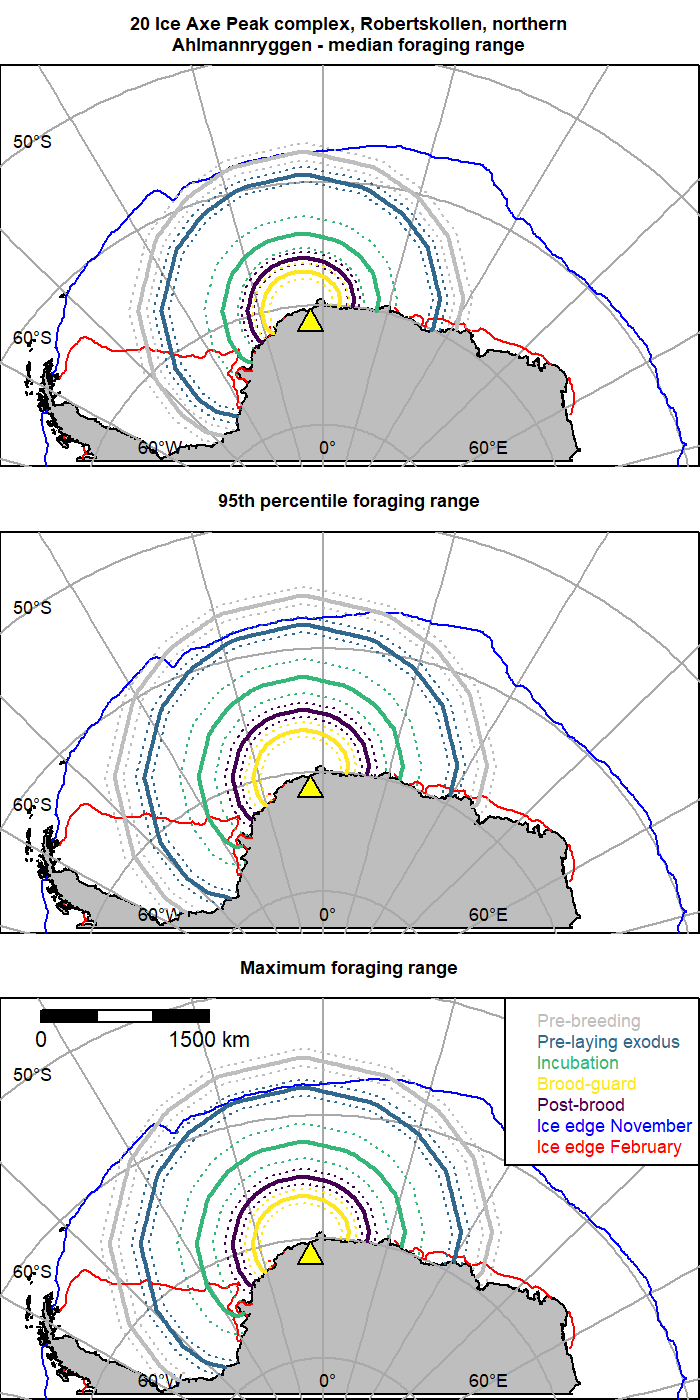

Supplement: Supplementary file 2 — Supplementary material 2 [file 40462_2025_609_MOESM2_ESM.zip › plots/bd_plot_20.png]

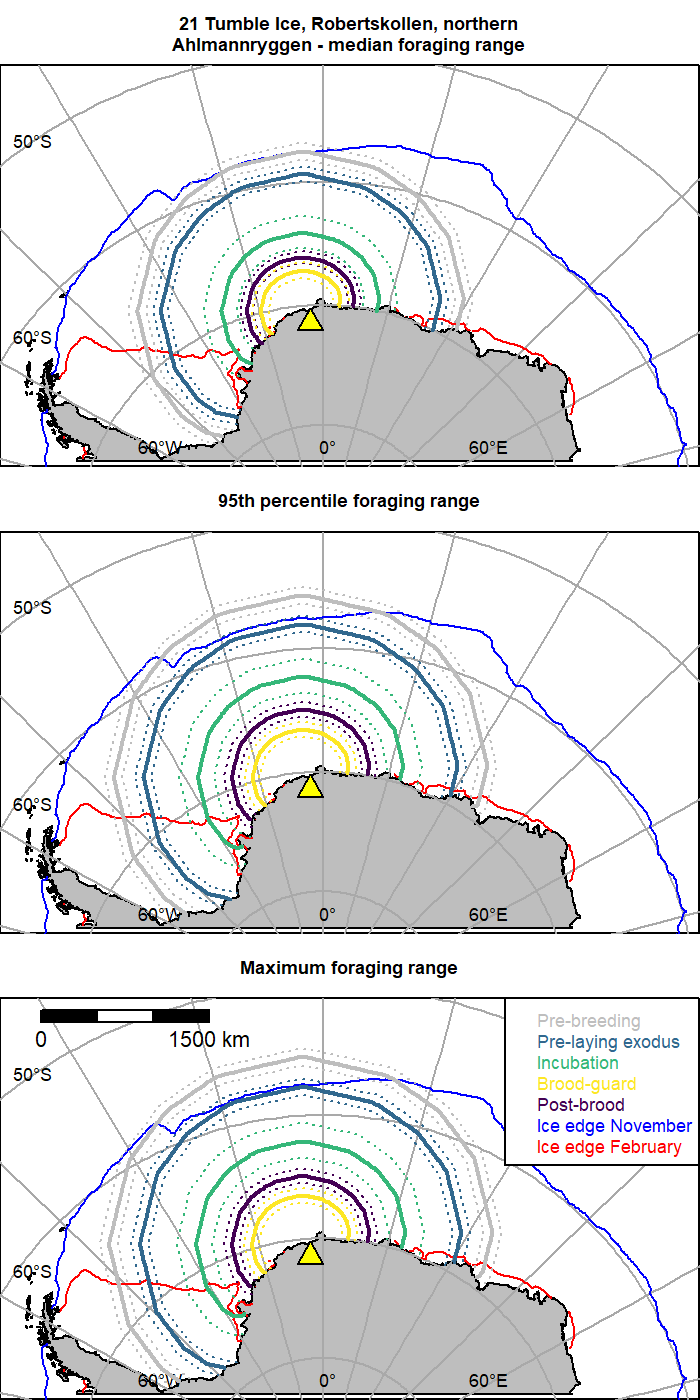

Supplement: Supplementary file 2 — Supplementary material 2 [file 40462_2025_609_MOESM2_ESM.zip › plots/bd_plot_21.png]

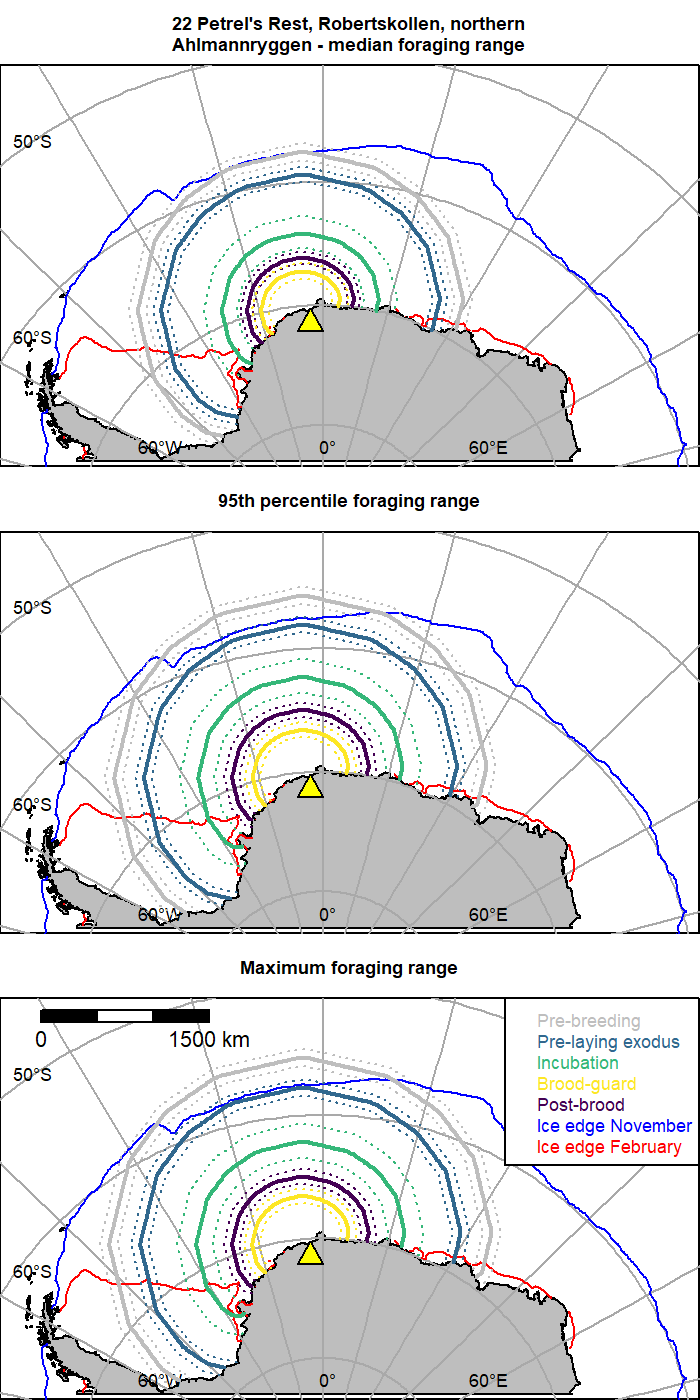

Supplement: Supplementary file 2 — Supplementary material 2 [file 40462_2025_609_MOESM2_ESM.zip › plots/bd_plot_22.png]

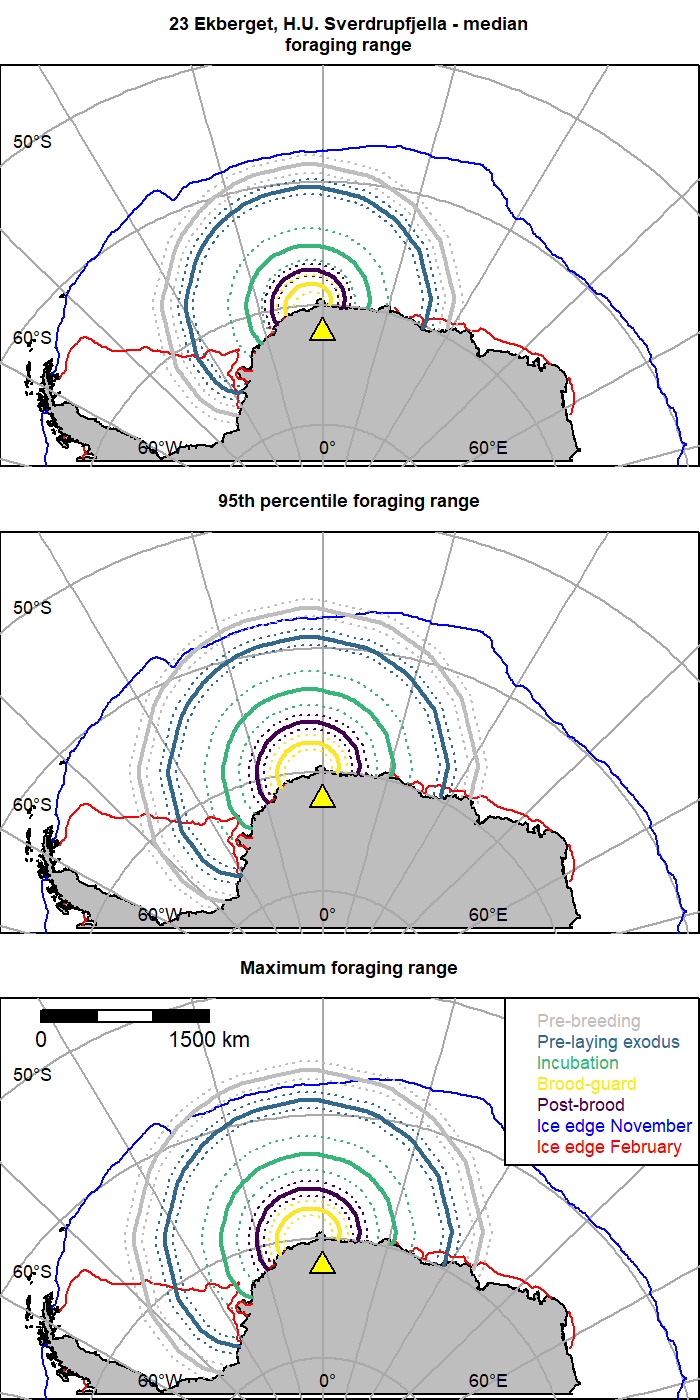

Supplement: Supplementary file 2 — Supplementary material 2 [file 40462_2025_609_MOESM2_ESM.zip › plots/bd_plot_23.png]

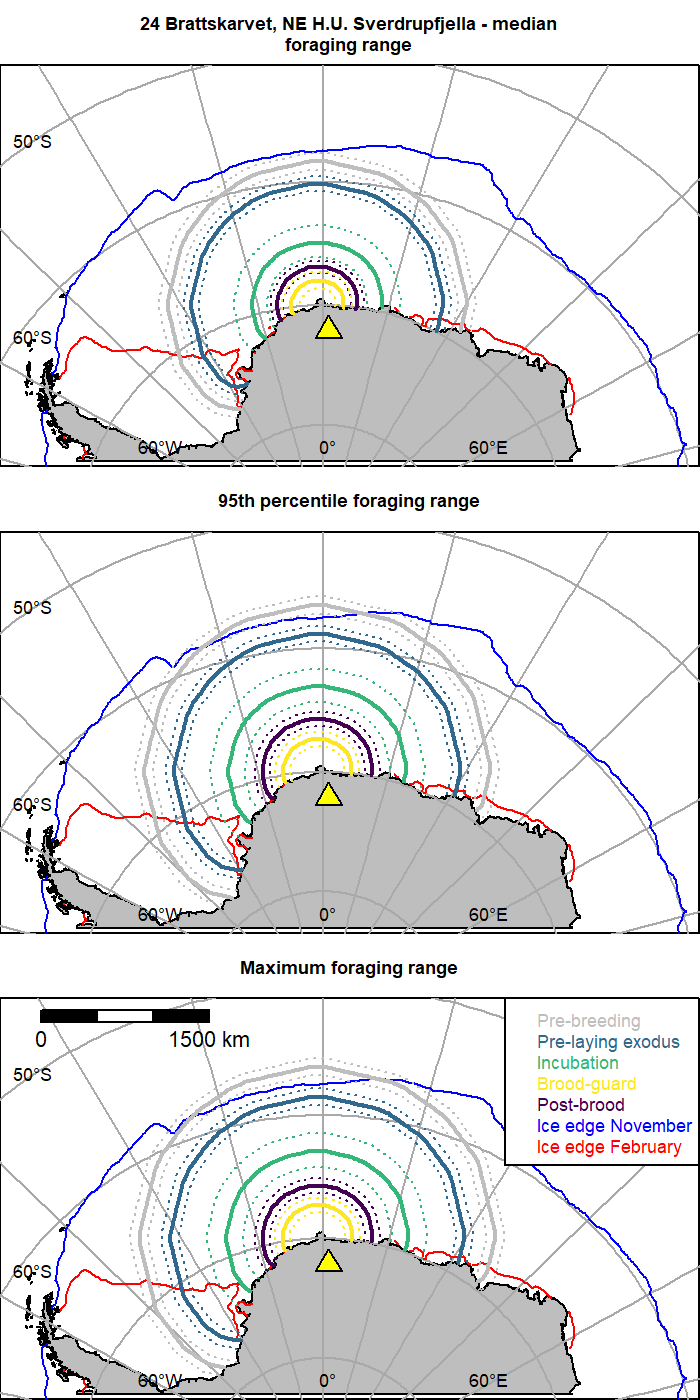

Supplement: Supplementary file 2 — Supplementary material 2 [file 40462_2025_609_MOESM2_ESM.zip › plots/bd_plot_24.png]

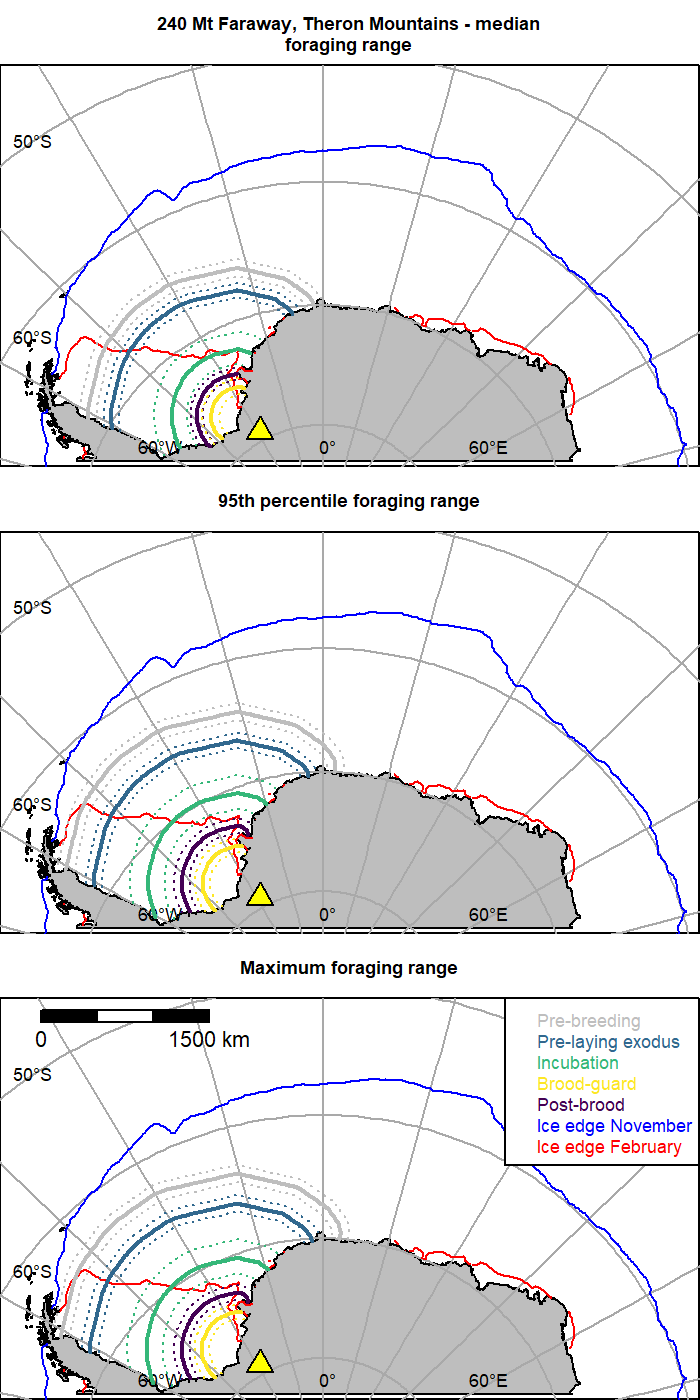

Supplement: Supplementary file 2 — Supplementary material 2 [file 40462_2025_609_MOESM2_ESM.zip › plots/bd_plot_240.png]

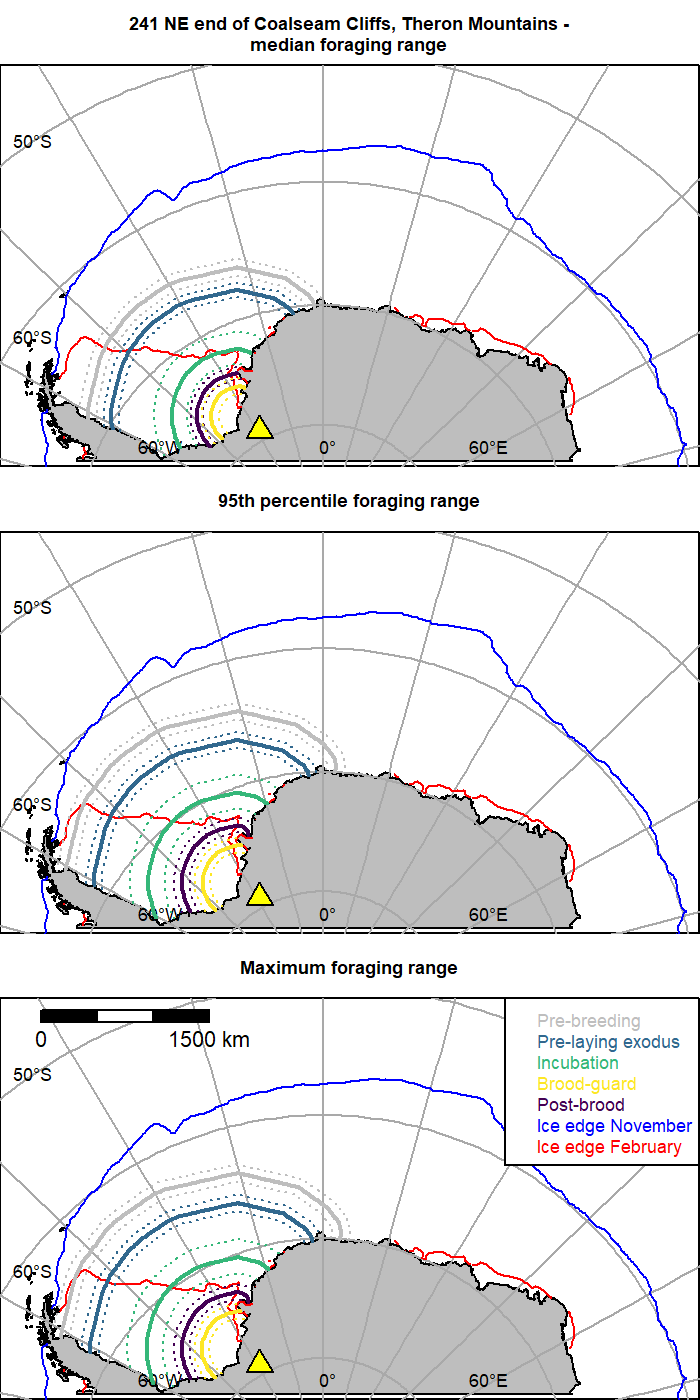

Supplement: Supplementary file 2 — Supplementary material 2 [file 40462_2025_609_MOESM2_ESM.zip › plots/bd_plot_241.png]

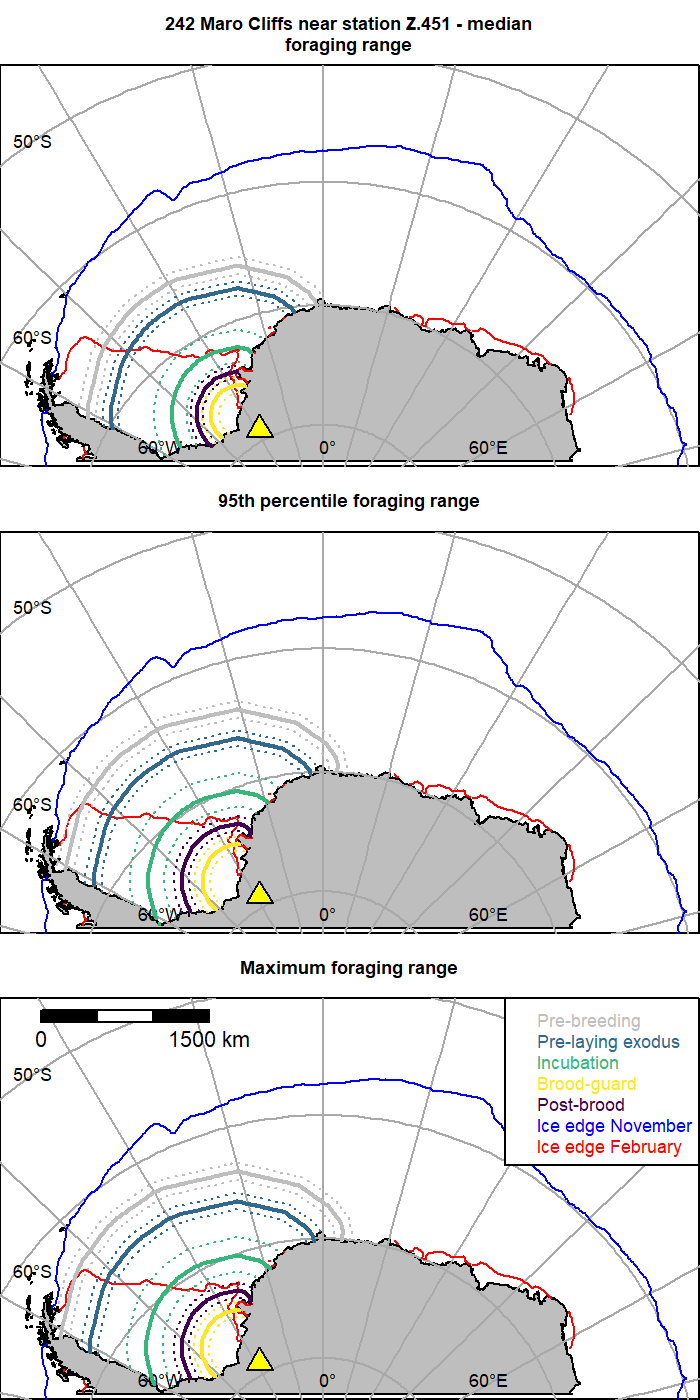

Supplement: Supplementary file 2 — Supplementary material 2 [file 40462_2025_609_MOESM2_ESM.zip › plots/bd_plot_242.png]

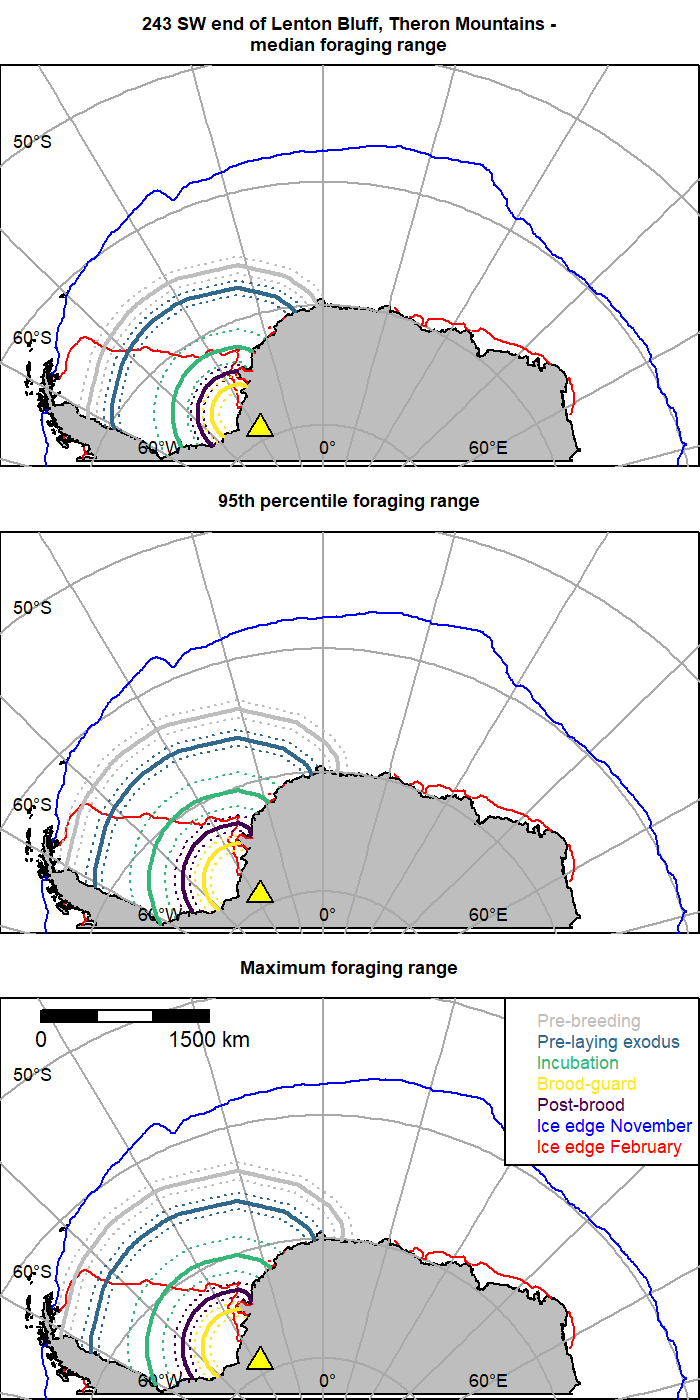

Supplement: Supplementary file 2 — Supplementary material 2 [file 40462_2025_609_MOESM2_ESM.zip › plots/bd_plot_243.png]

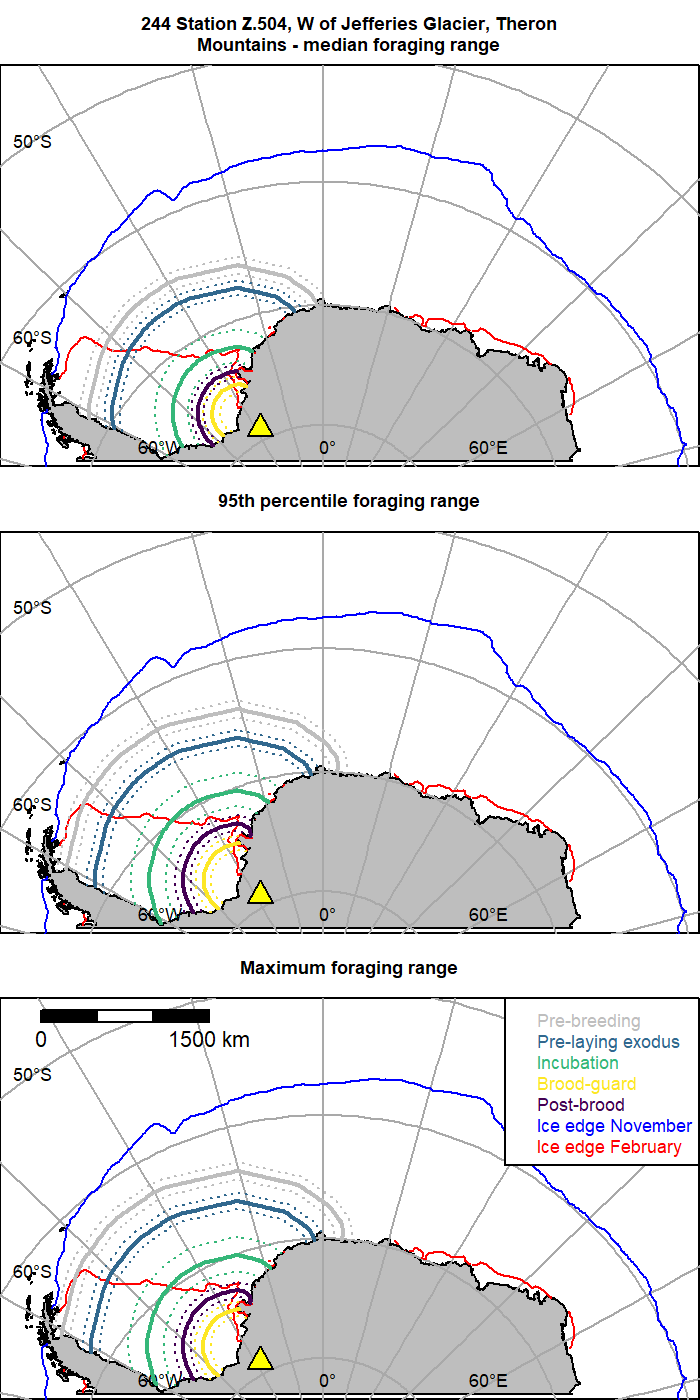

Supplement: Supplementary file 2 — Supplementary material 2 [file 40462_2025_609_MOESM2_ESM.zip › plots/bd_plot_244.png]

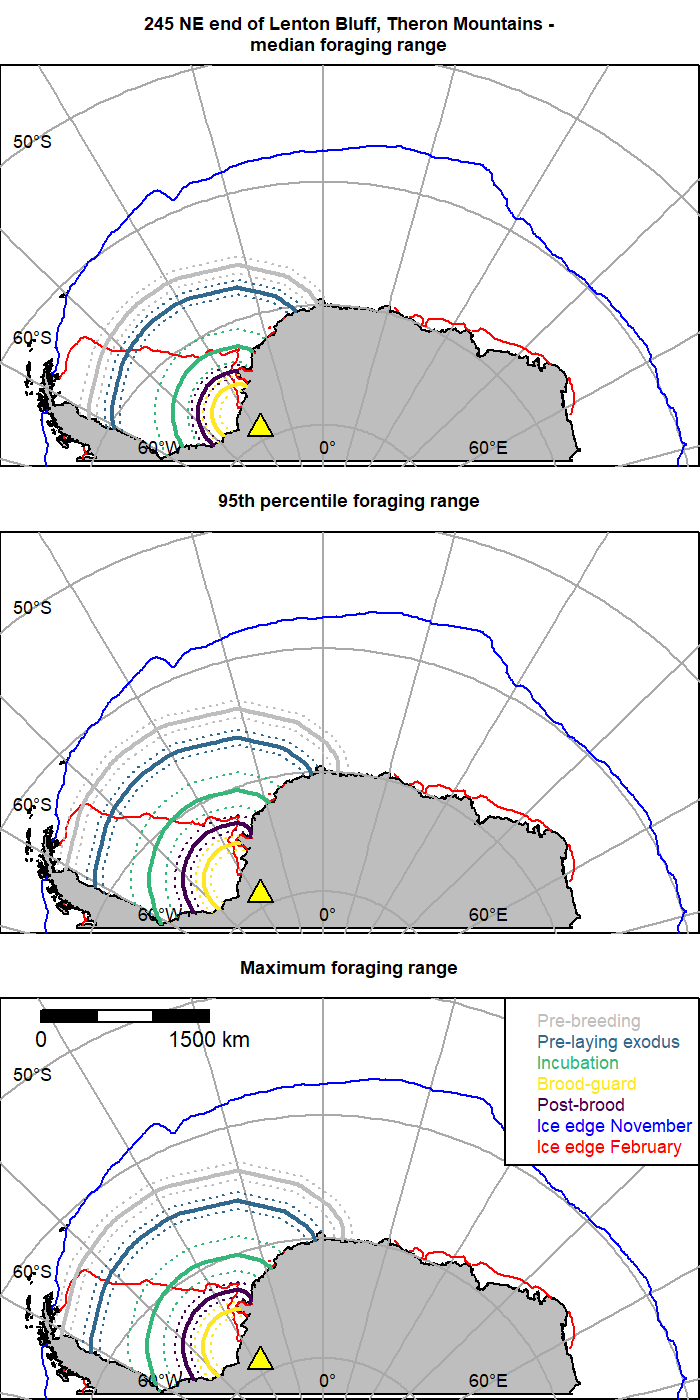

Supplement: Supplementary file 2 — Supplementary material 2 [file 40462_2025_609_MOESM2_ESM.zip › plots/bd_plot_245.png]

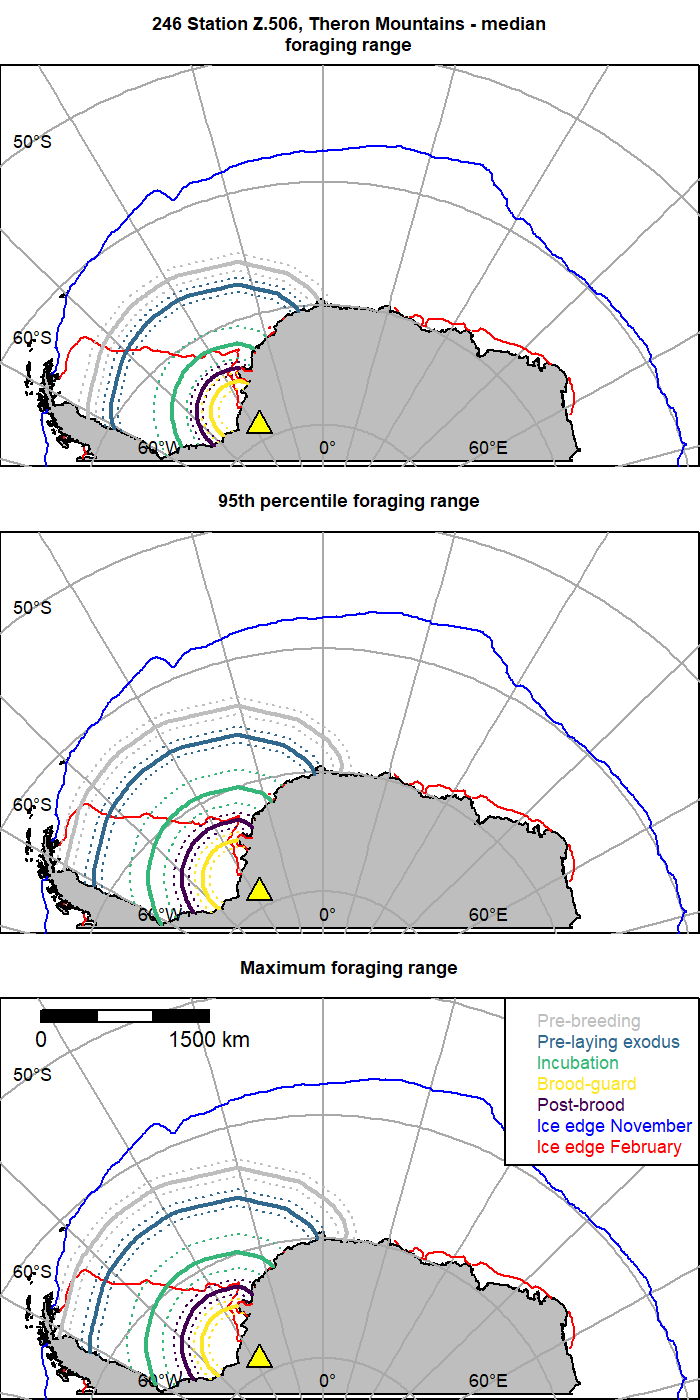

Supplement: Supplementary file 2 — Supplementary material 2 [file 40462_2025_609_MOESM2_ESM.zip › plots/bd_plot_246.png]

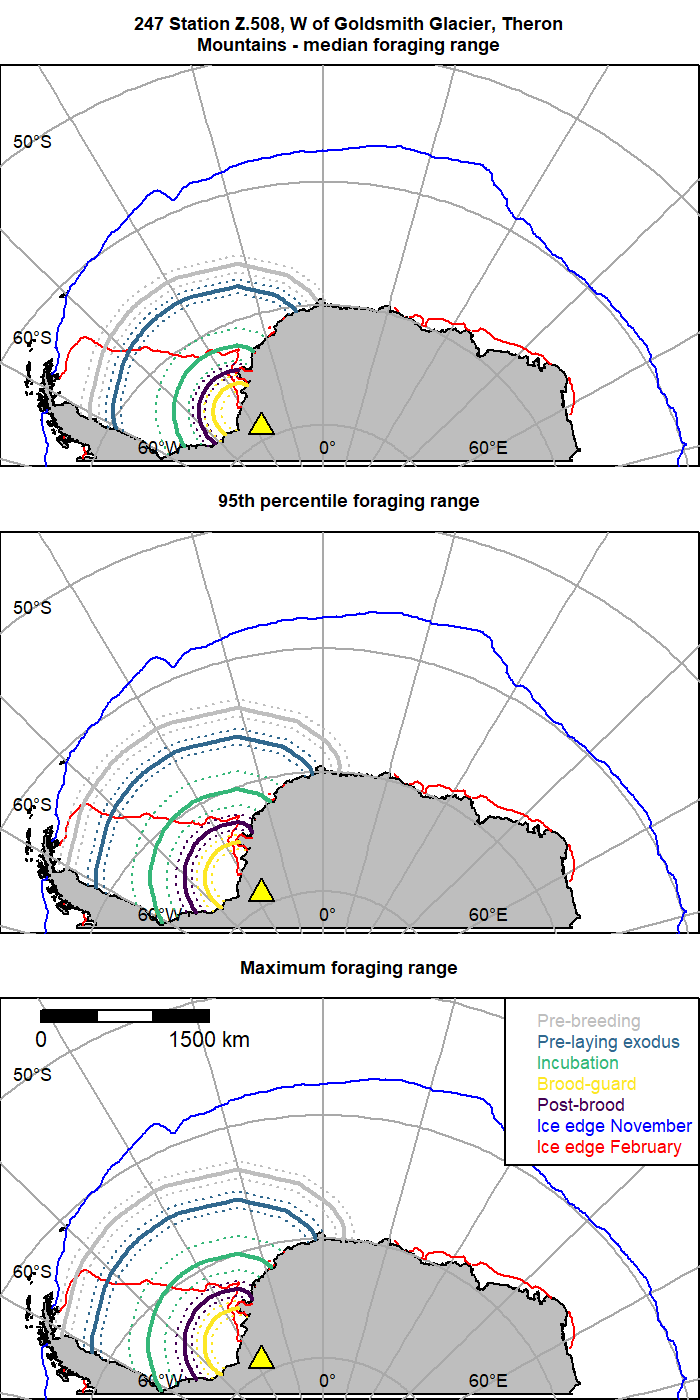

Supplement: Supplementary file 2 — Supplementary material 2 [file 40462_2025_609_MOESM2_ESM.zip › plots/bd_plot_247.png]

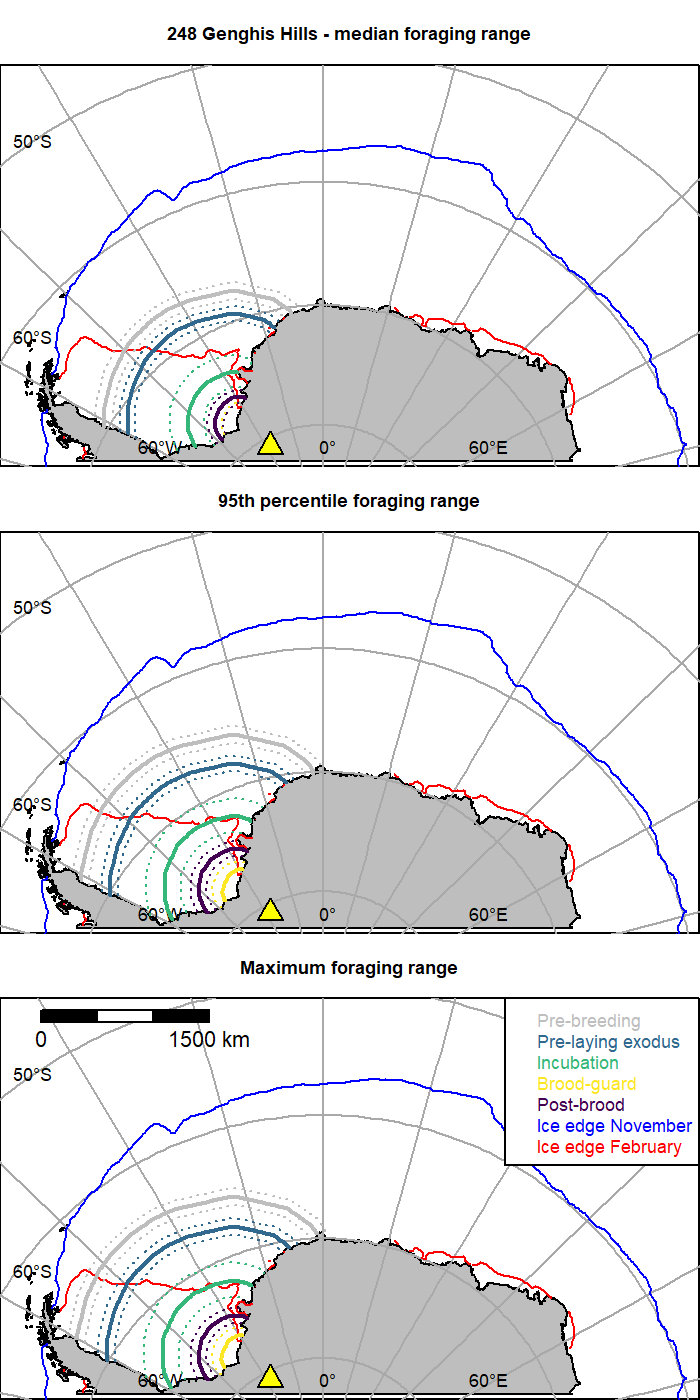

Supplement: Supplementary file 2 — Supplementary material 2 [file 40462_2025_609_MOESM2_ESM.zip › plots/bd_plot_248.png]

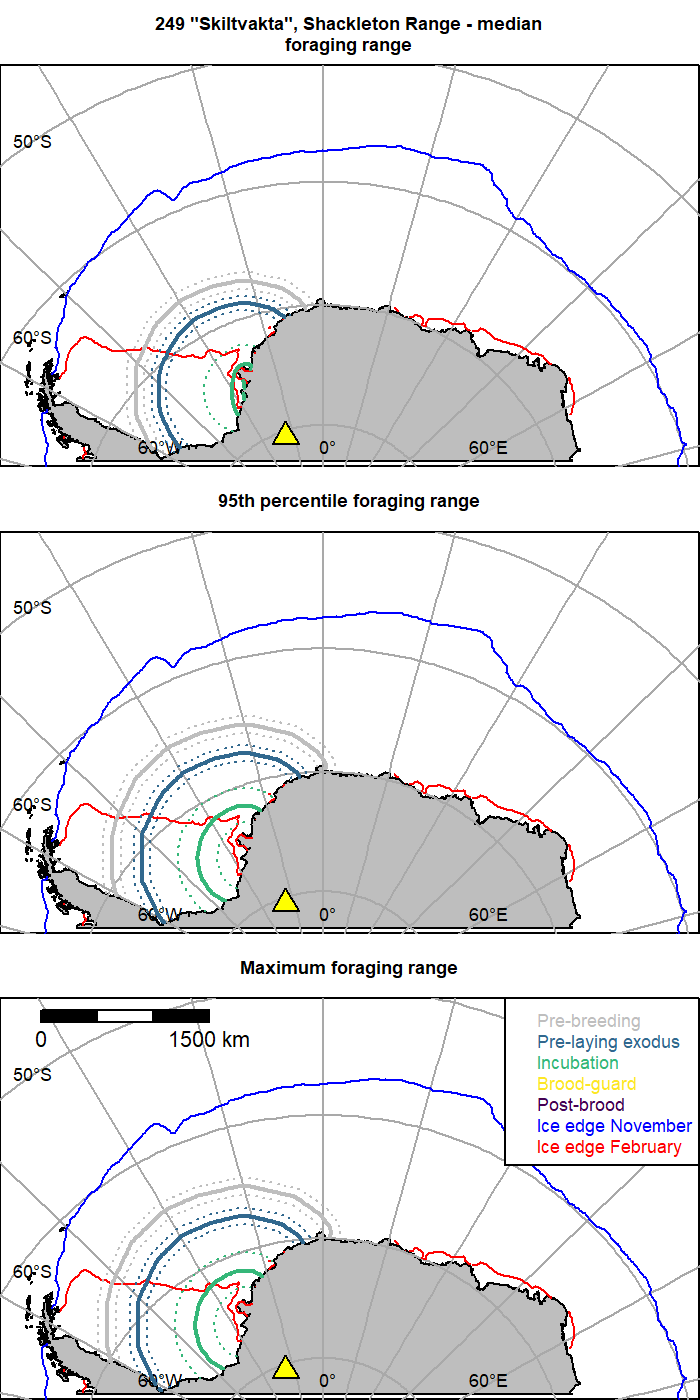

Supplement: Supplementary file 2 — Supplementary material 2 [file 40462_2025_609_MOESM2_ESM.zip › plots/bd_plot_249.png]

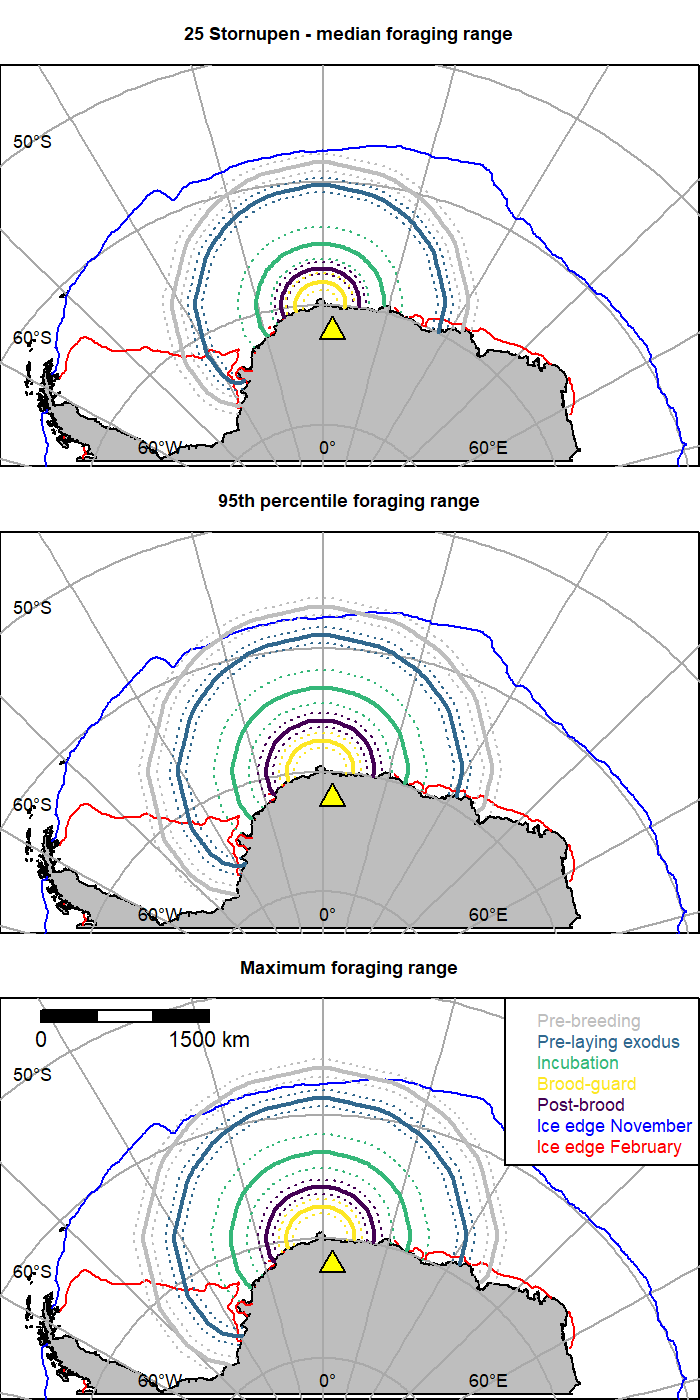

Supplement: Supplementary file 2 — Supplementary material 2 [file 40462_2025_609_MOESM2_ESM.zip › plots/bd_plot_25.png]

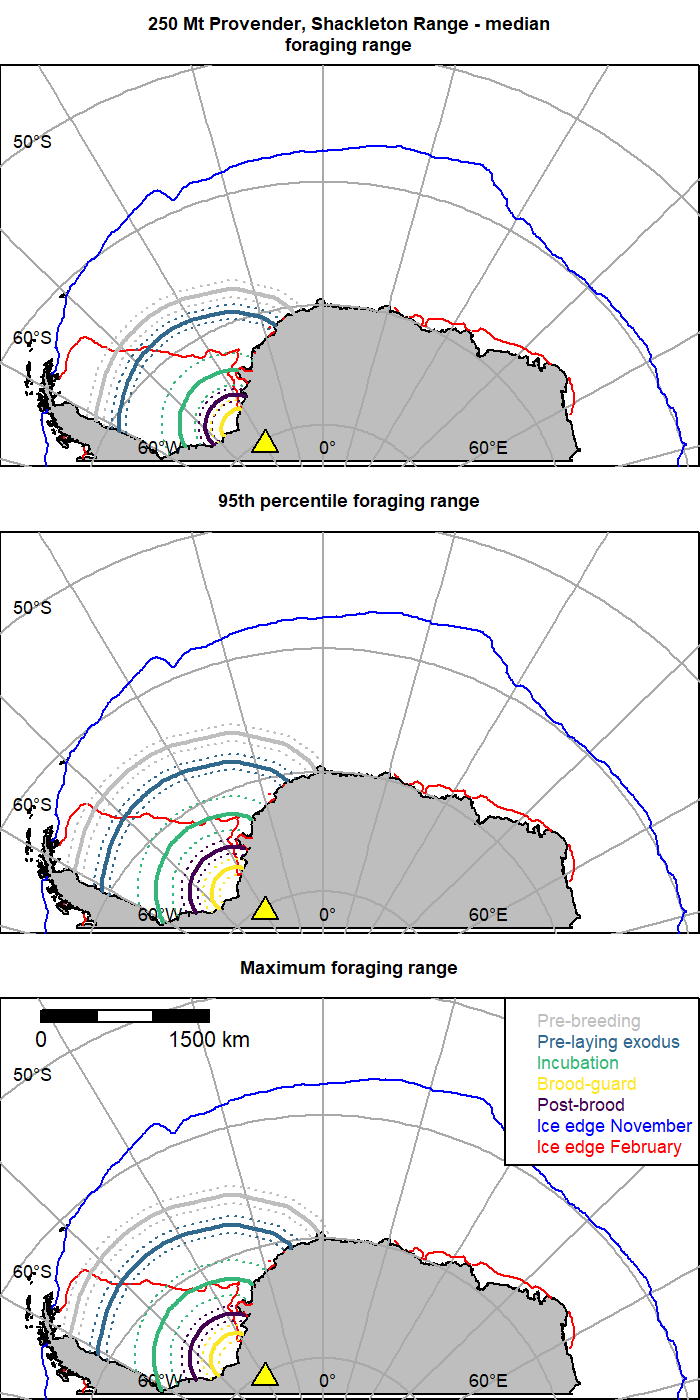

Supplement: Supplementary file 2 — Supplementary material 2 [file 40462_2025_609_MOESM2_ESM.zip › plots/bd_plot_250.png]

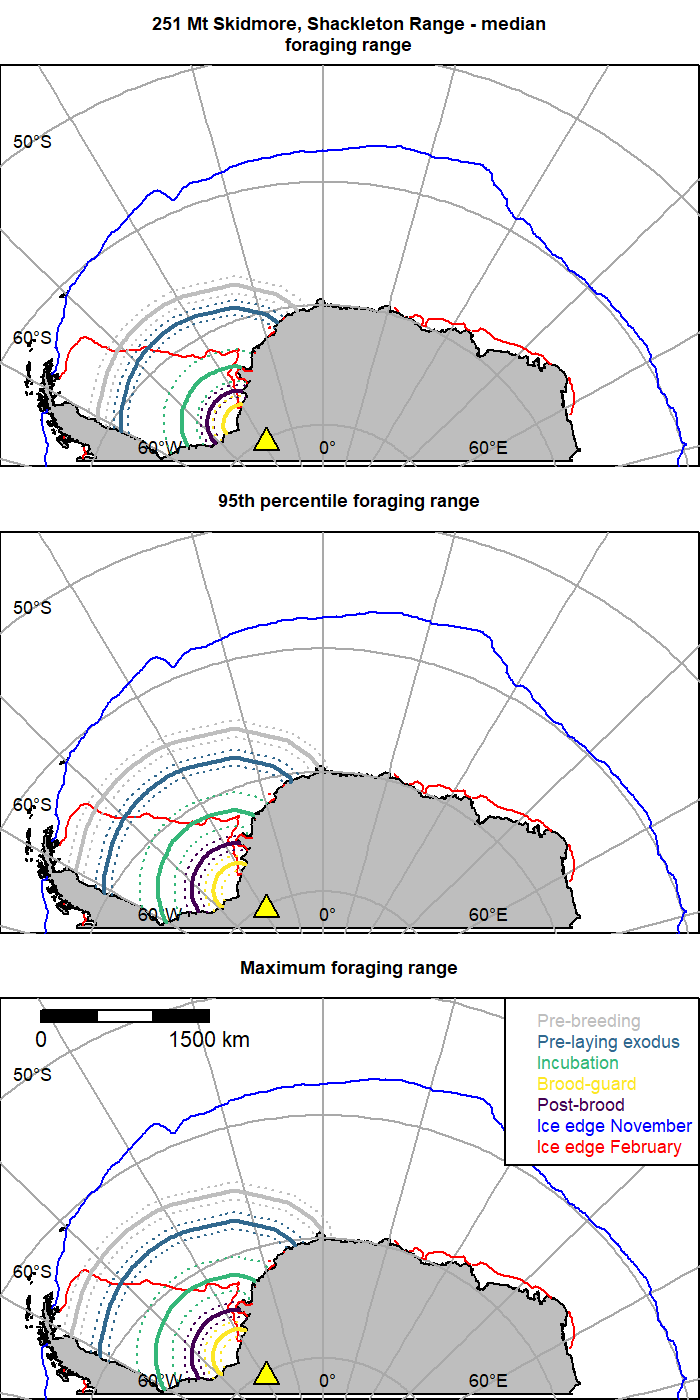

Supplement: Supplementary file 2 — Supplementary material 2 [file 40462_2025_609_MOESM2_ESM.zip › plots/bd_plot_251.png]

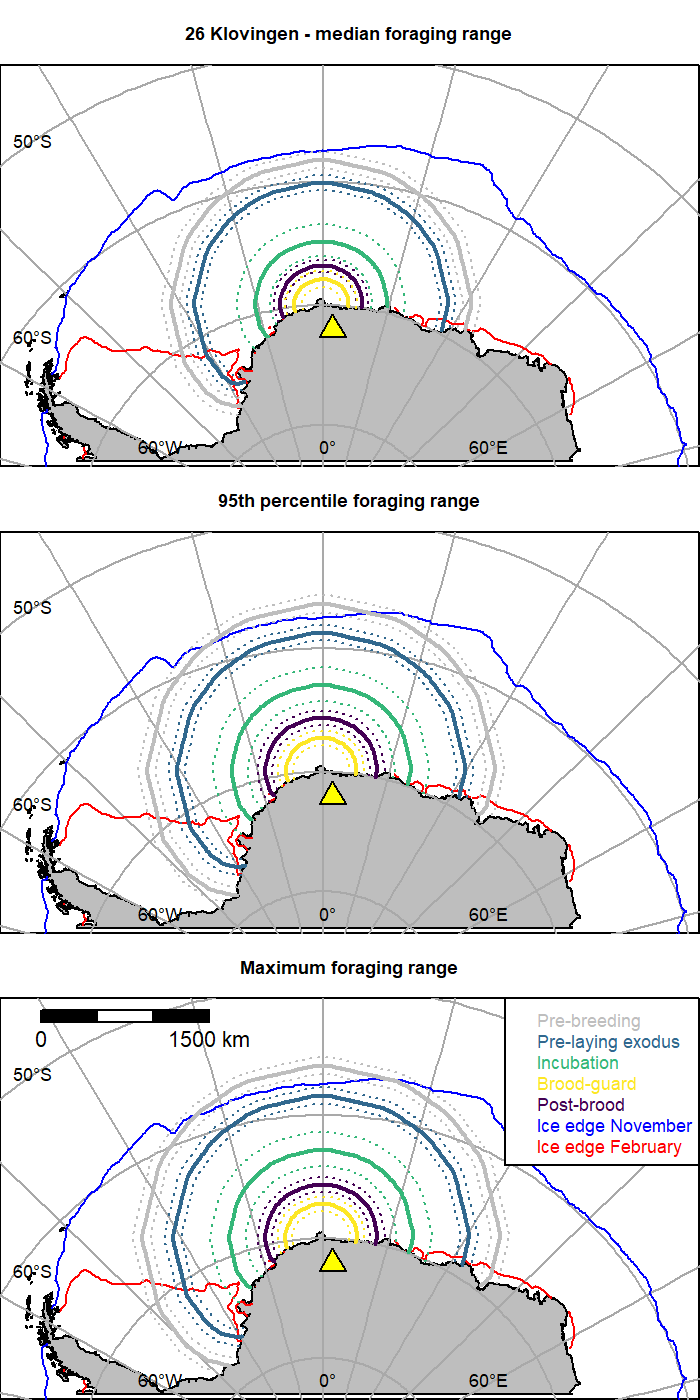

Supplement: Supplementary file 2 — Supplementary material 2 [file 40462_2025_609_MOESM2_ESM.zip › plots/bd_plot_26.png]

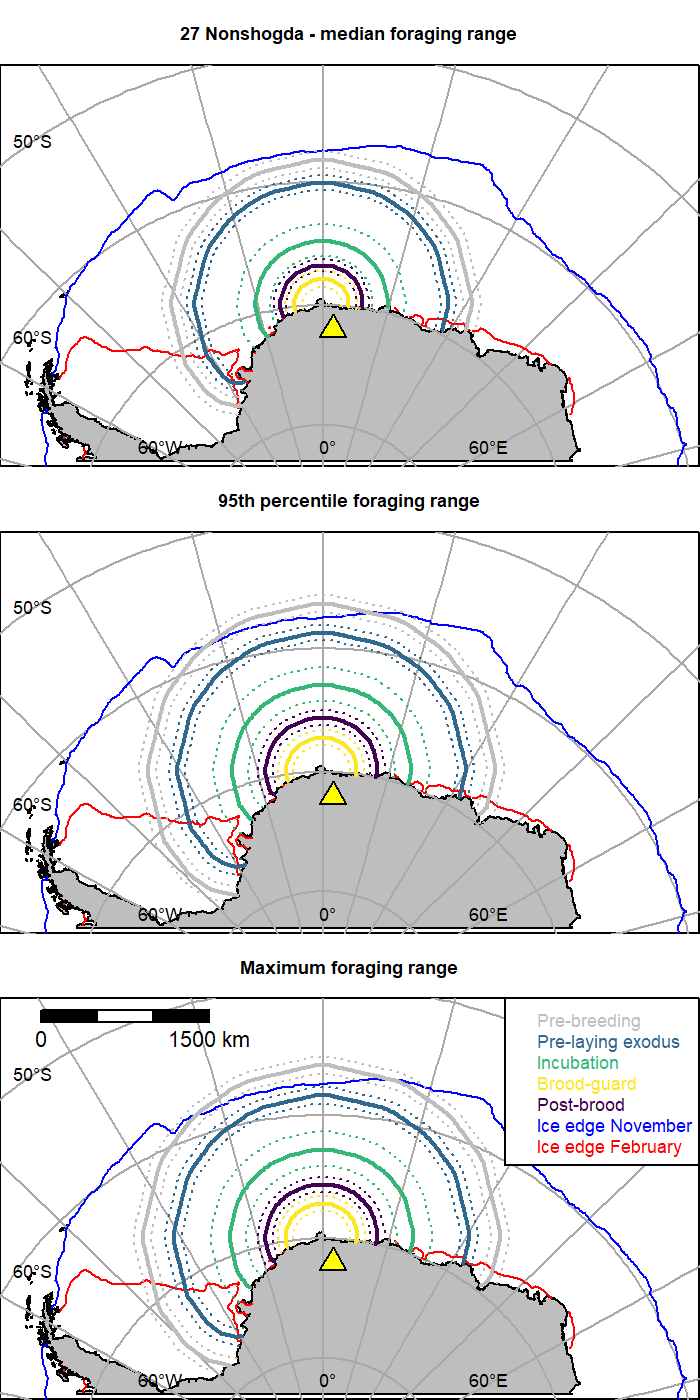

Supplement: Supplementary file 2 — Supplementary material 2 [file 40462_2025_609_MOESM2_ESM.zip › plots/bd_plot_27.png]

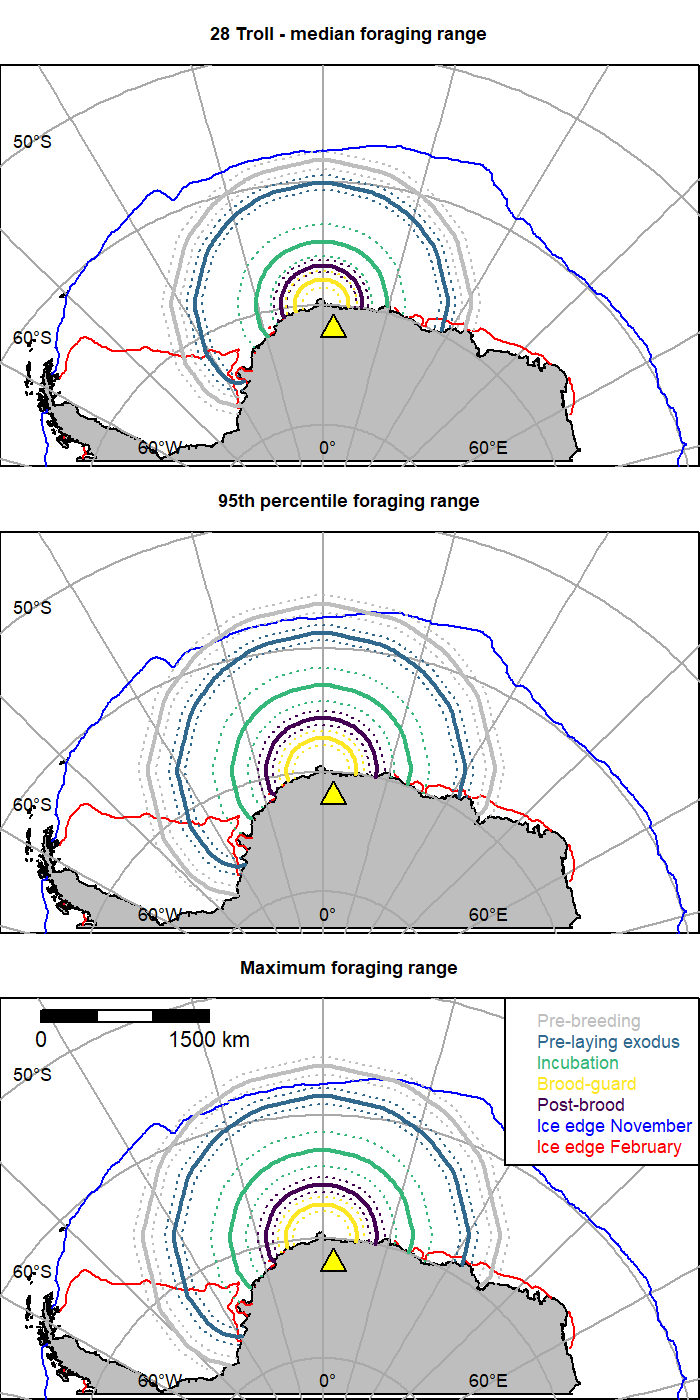

Supplement: Supplementary file 2 — Supplementary material 2 [file 40462_2025_609_MOESM2_ESM.zip › plots/bd_plot_28.png]

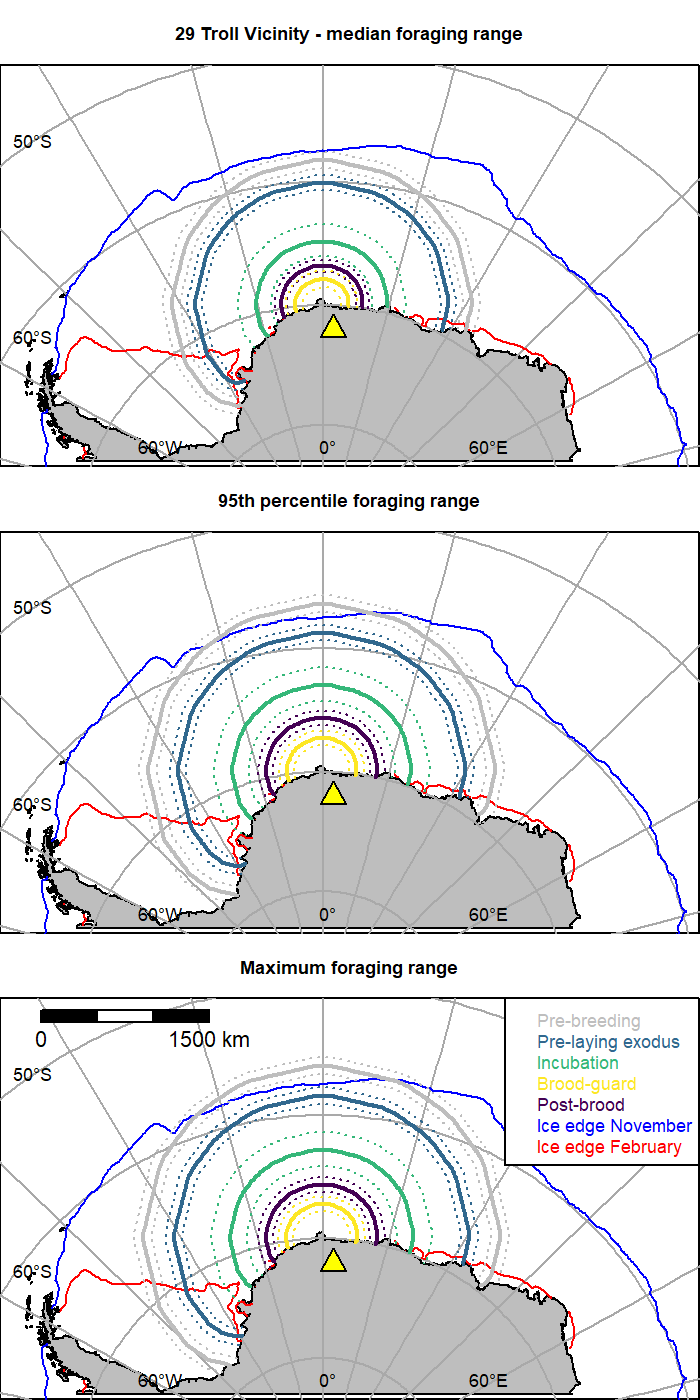

Supplement: Supplementary file 2 — Supplementary material 2 [file 40462_2025_609_MOESM2_ESM.zip › plots/bd_plot_29.png]

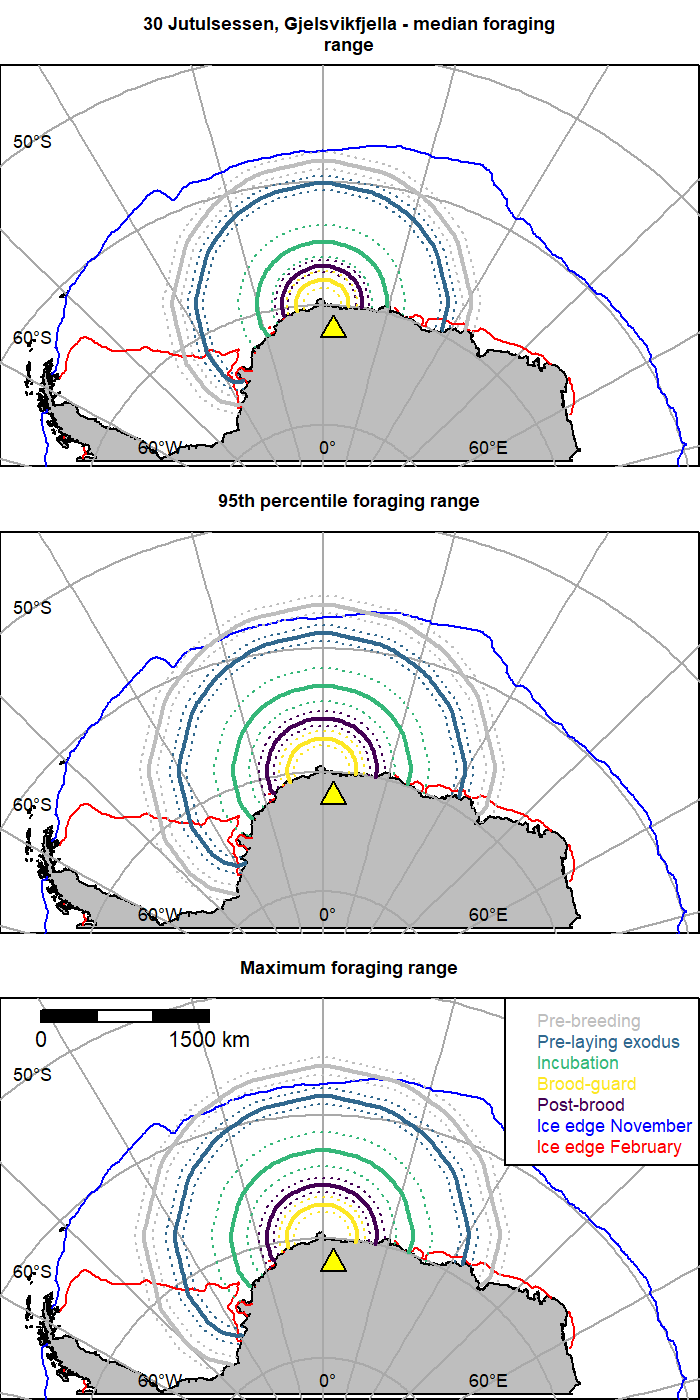

Supplement: Supplementary file 2 — Supplementary material 2 [file 40462_2025_609_MOESM2_ESM.zip › plots/bd_plot_30.png]

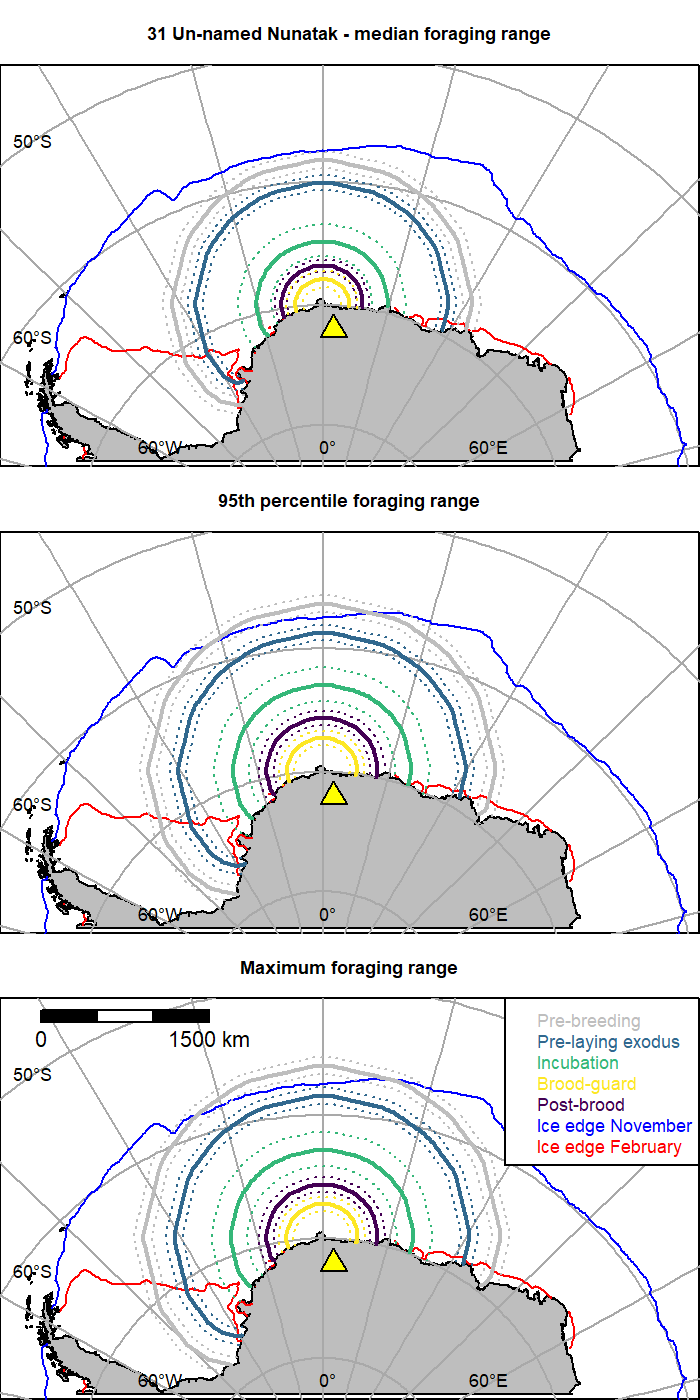

Supplement: Supplementary file 2 — Supplementary material 2 [file 40462_2025_609_MOESM2_ESM.zip › plots/bd_plot_31.png]

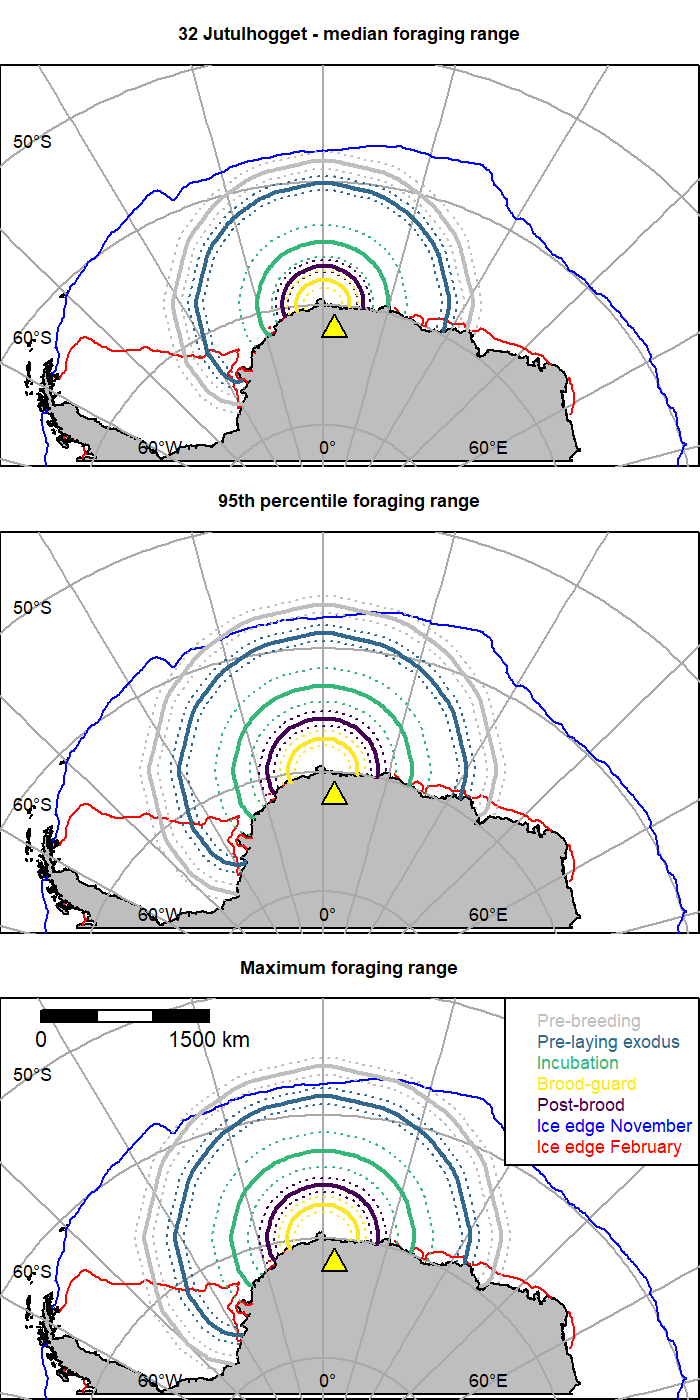

Supplement: Supplementary file 2 — Supplementary material 2 [file 40462_2025_609_MOESM2_ESM.zip › plots/bd_plot_32.png]

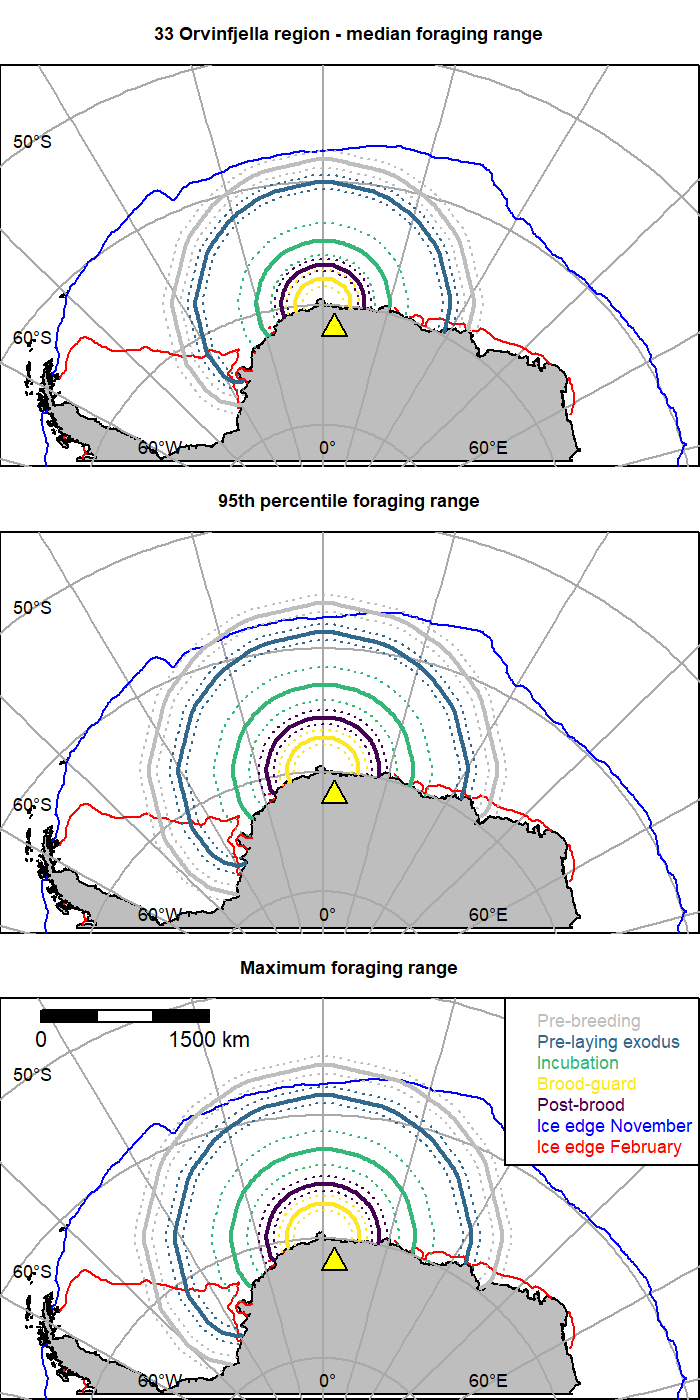

Supplement: Supplementary file 2 — Supplementary material 2 [file 40462_2025_609_MOESM2_ESM.zip › plots/bd_plot_33.png]

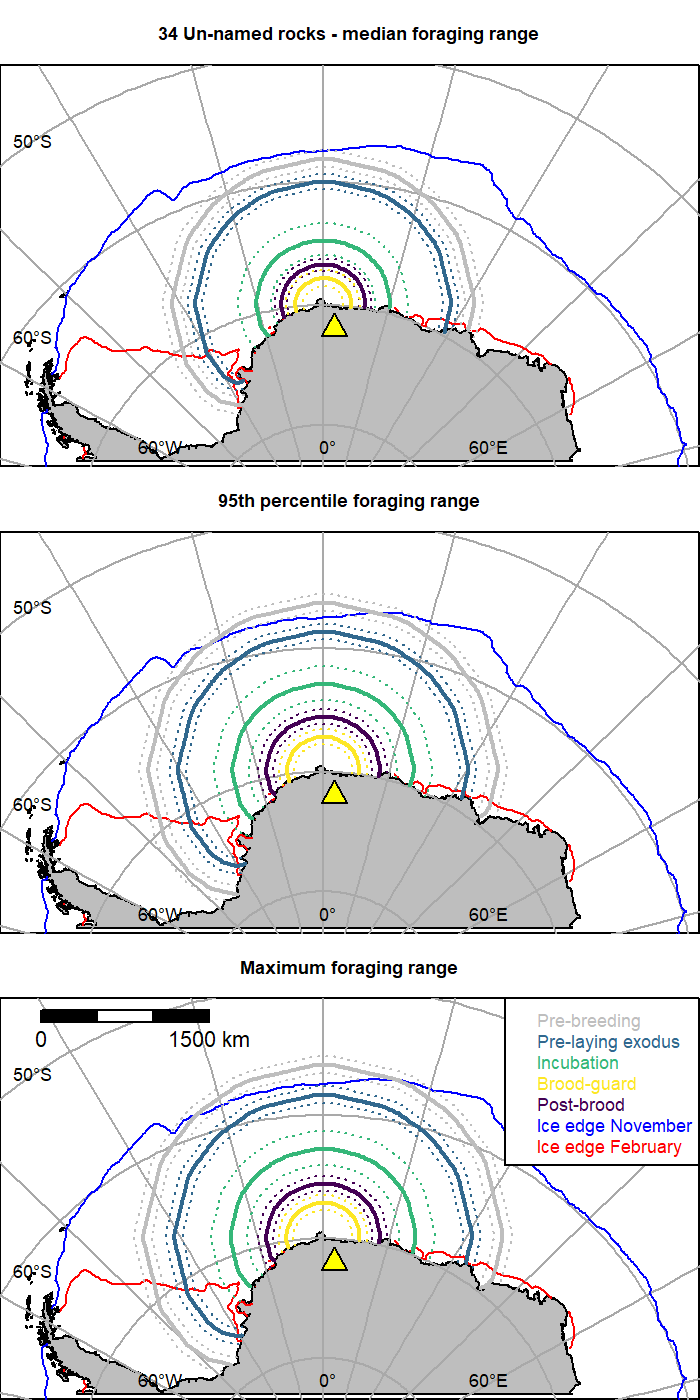

Supplement: Supplementary file 2 — Supplementary material 2 [file 40462_2025_609_MOESM2_ESM.zip › plots/bd_plot_34.png]

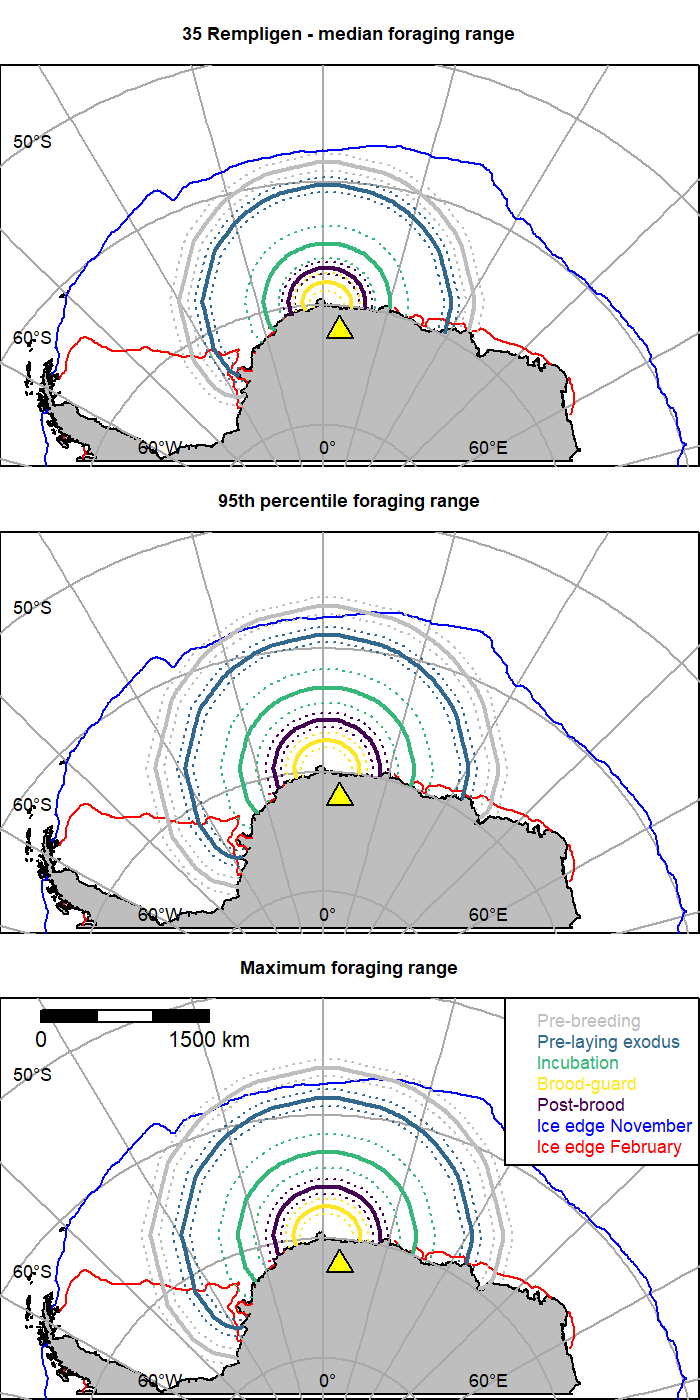

Supplement: Supplementary file 2 — Supplementary material 2 [file 40462_2025_609_MOESM2_ESM.zip › plots/bd_plot_35.png]

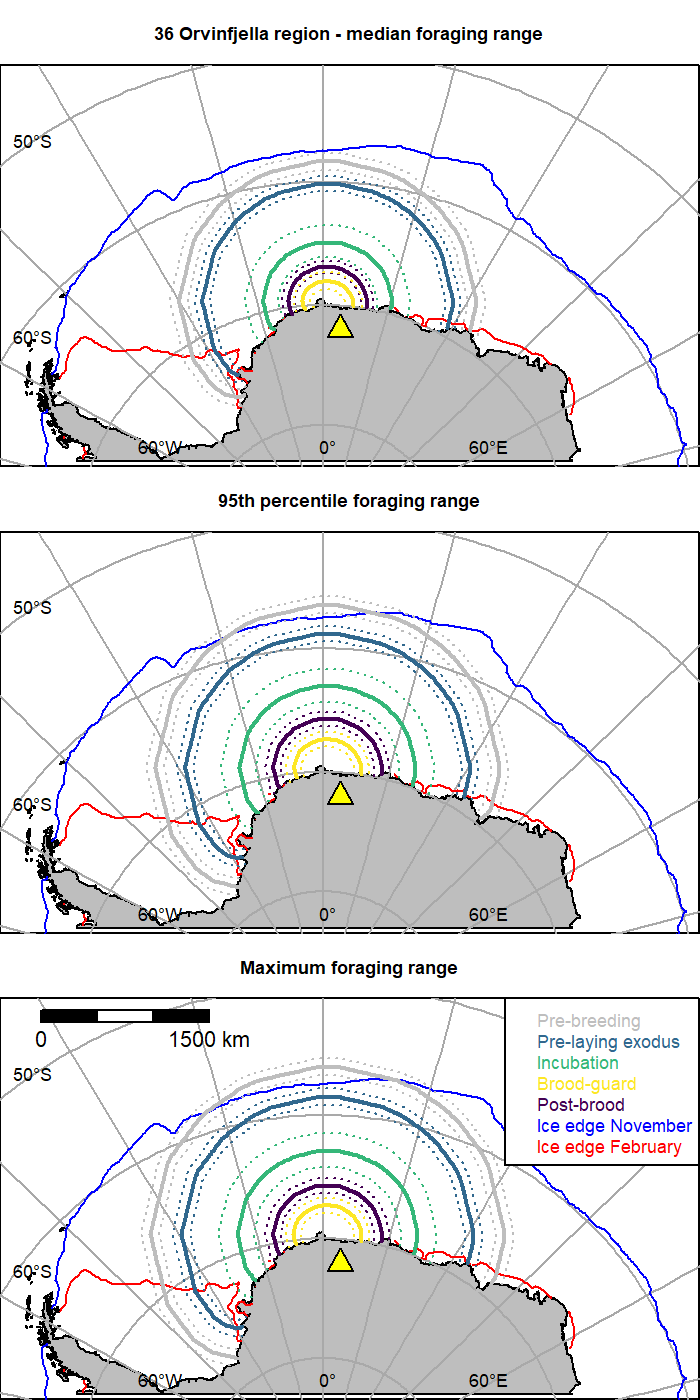

Supplement: Supplementary file 2 — Supplementary material 2 [file 40462_2025_609_MOESM2_ESM.zip › plots/bd_plot_36.png]

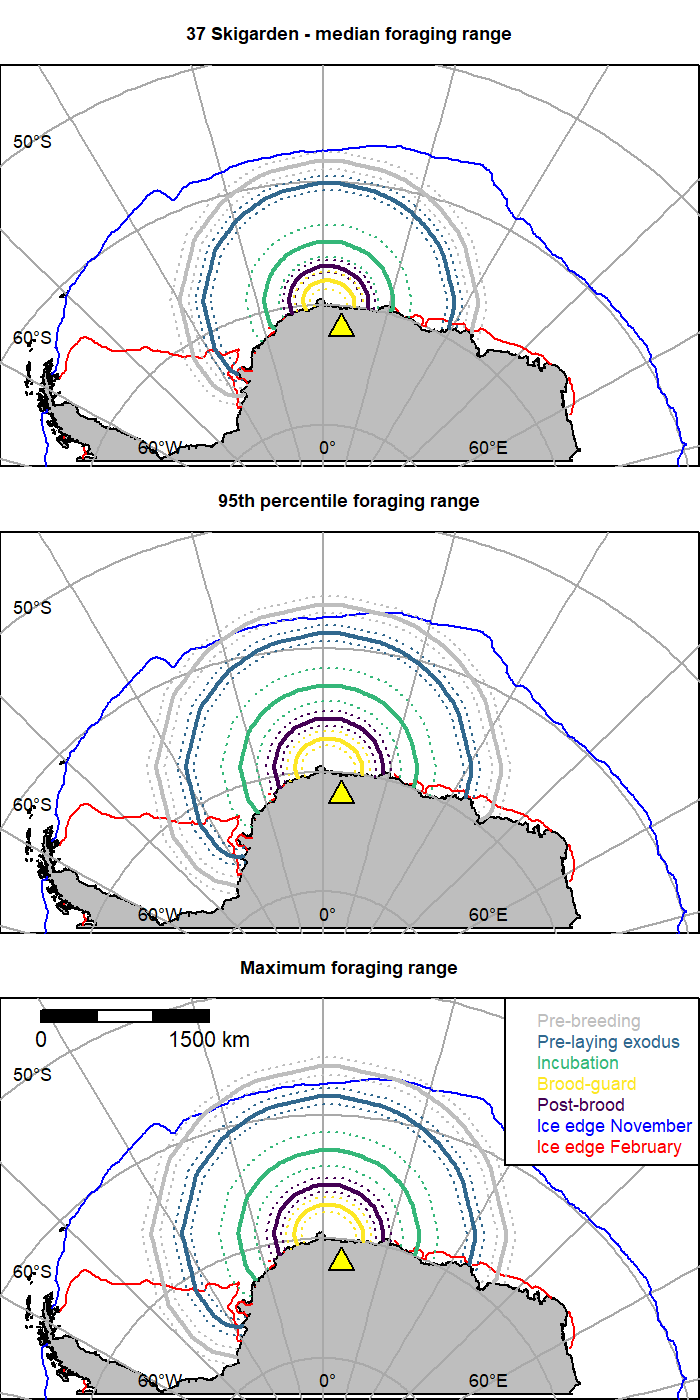

Supplement: Supplementary file 2 — Supplementary material 2 [file 40462_2025_609_MOESM2_ESM.zip › plots/bd_plot_37.png]

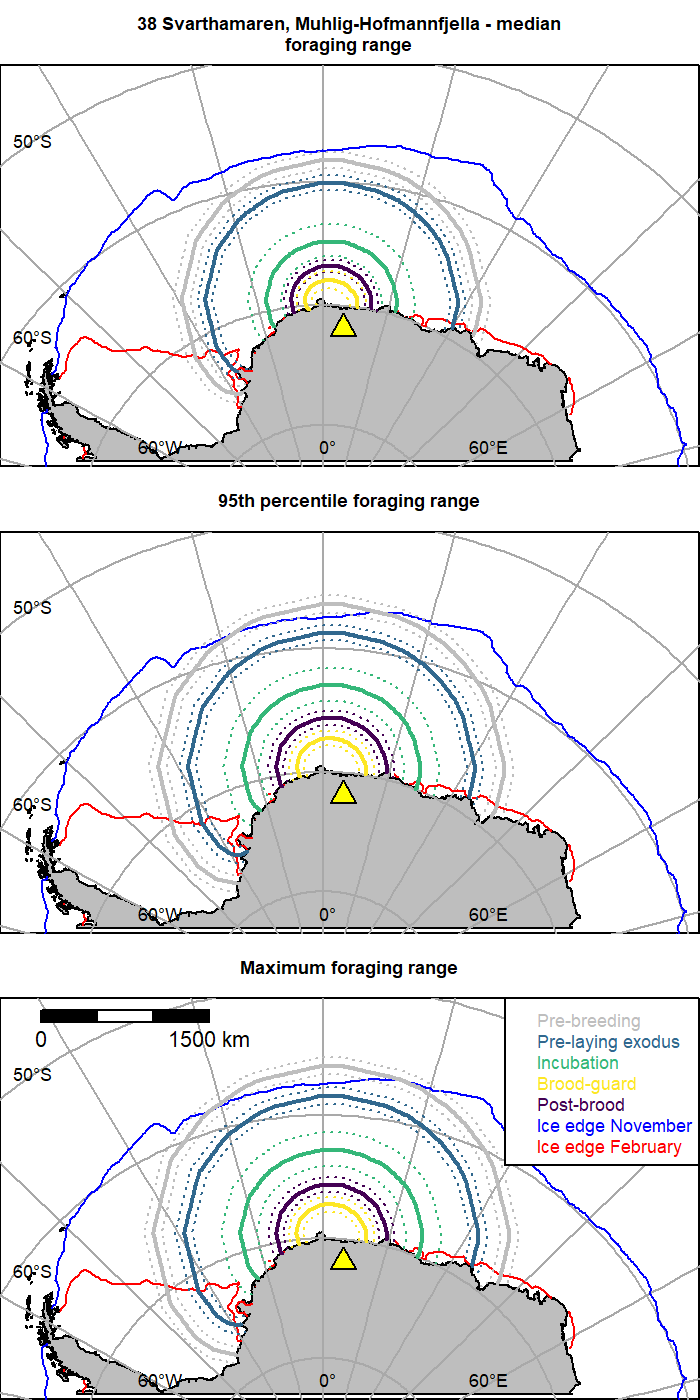

Supplement: Supplementary file 2 — Supplementary material 2 [file 40462_2025_609_MOESM2_ESM.zip › plots/bd_plot_38.png]

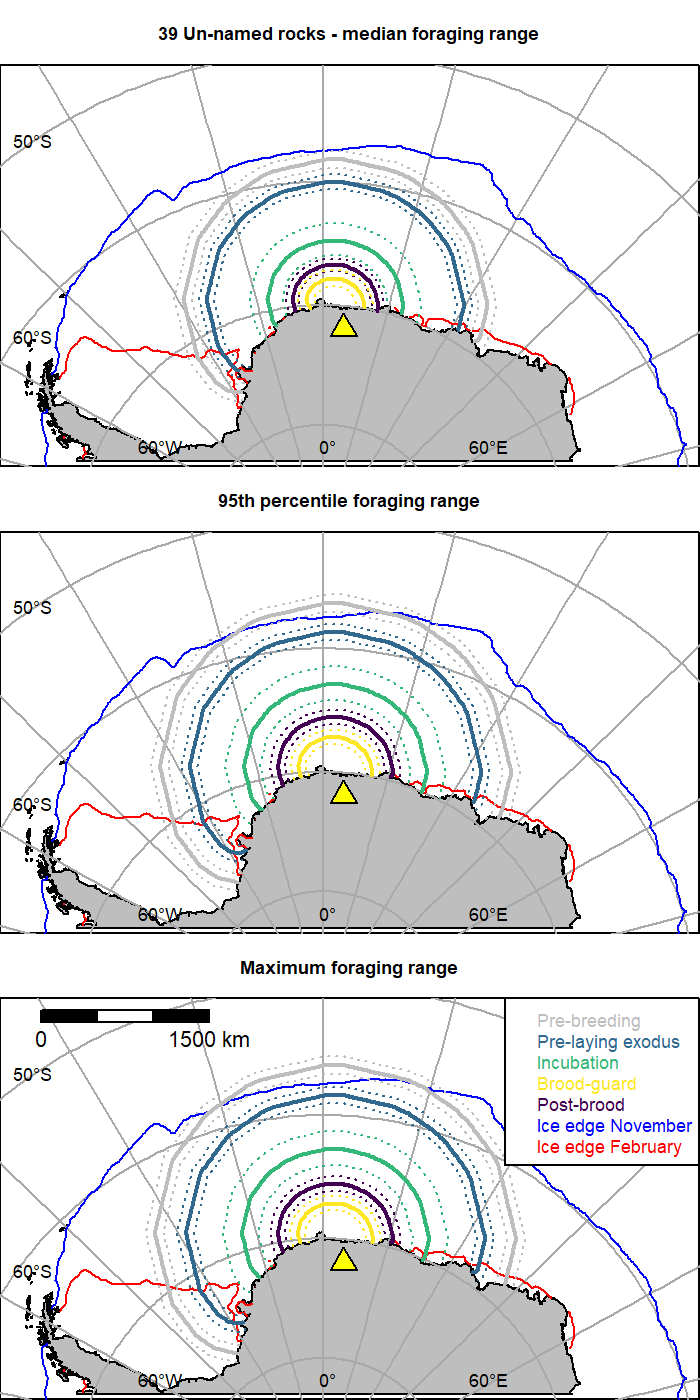

Supplement: Supplementary file 2 — Supplementary material 2 [file 40462_2025_609_MOESM2_ESM.zip › plots/bd_plot_39.png]

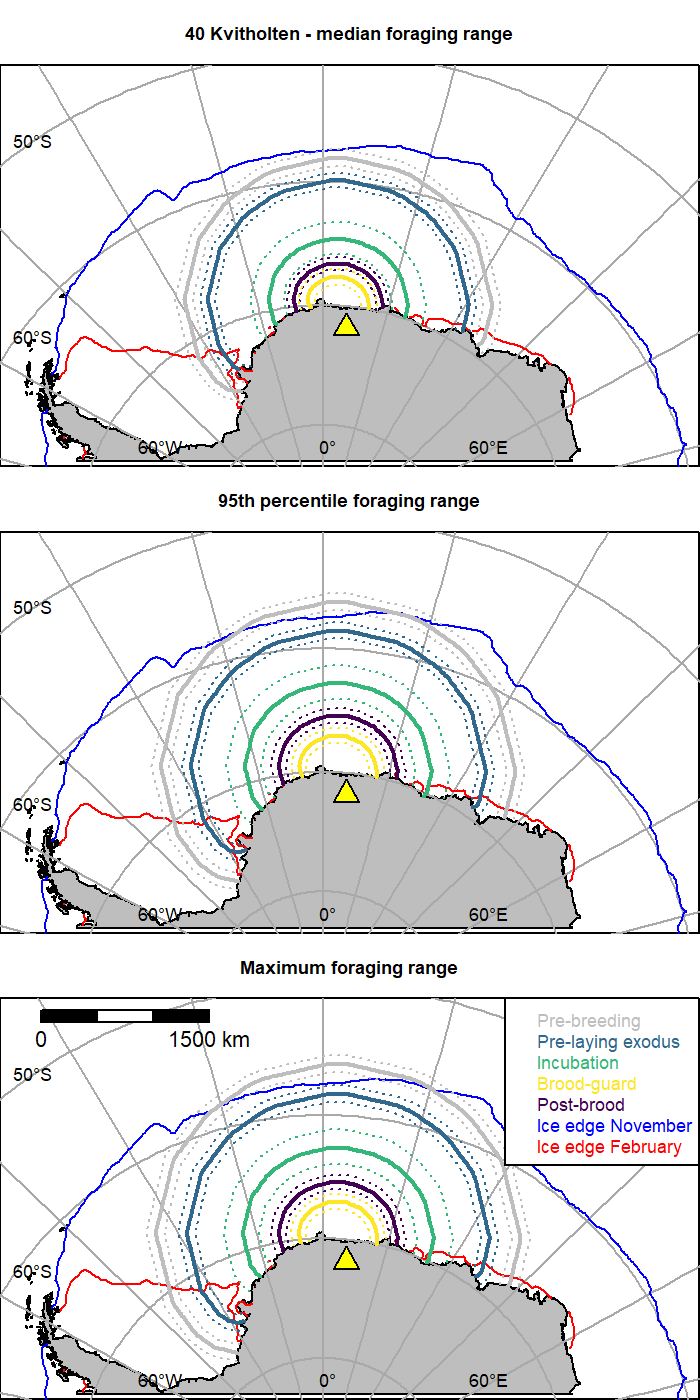

Supplement: Supplementary file 2 — Supplementary material 2 [file 40462_2025_609_MOESM2_ESM.zip › plots/bd_plot_40.png]

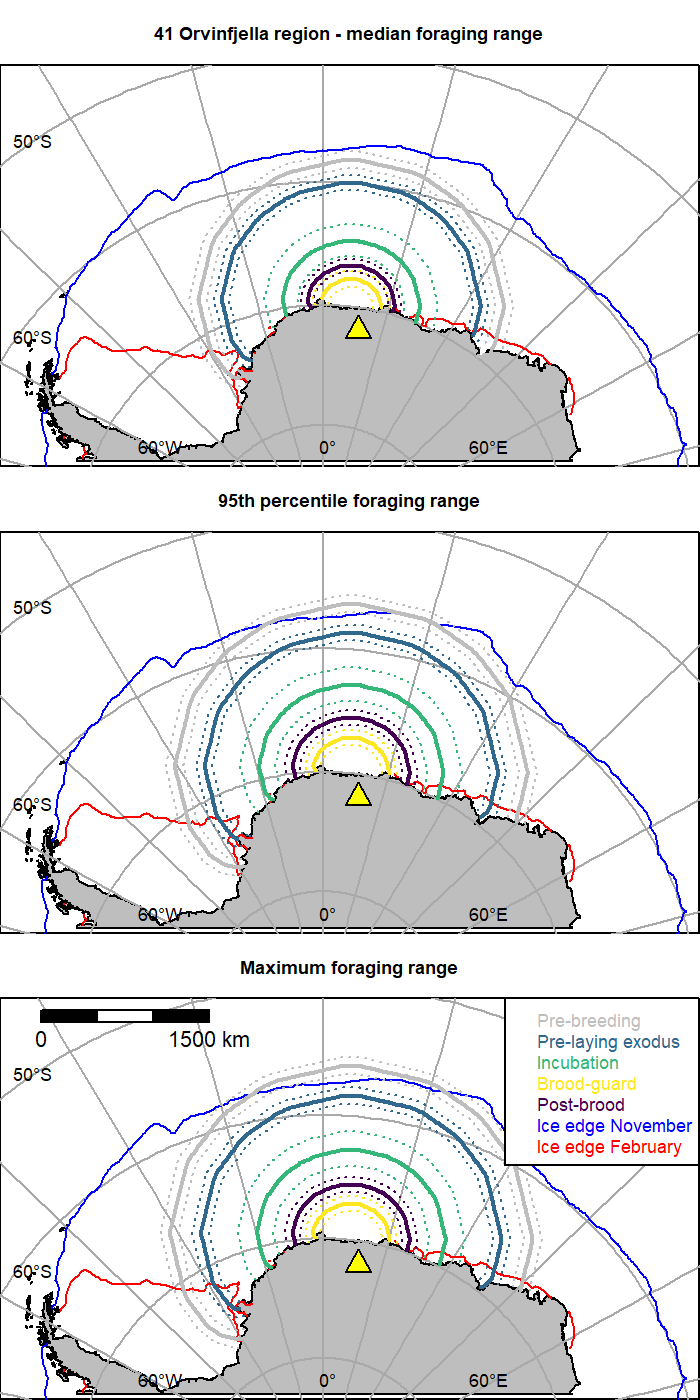

Supplement: Supplementary file 2 — Supplementary material 2 [file 40462_2025_609_MOESM2_ESM.zip › plots/bd_plot_41.png]

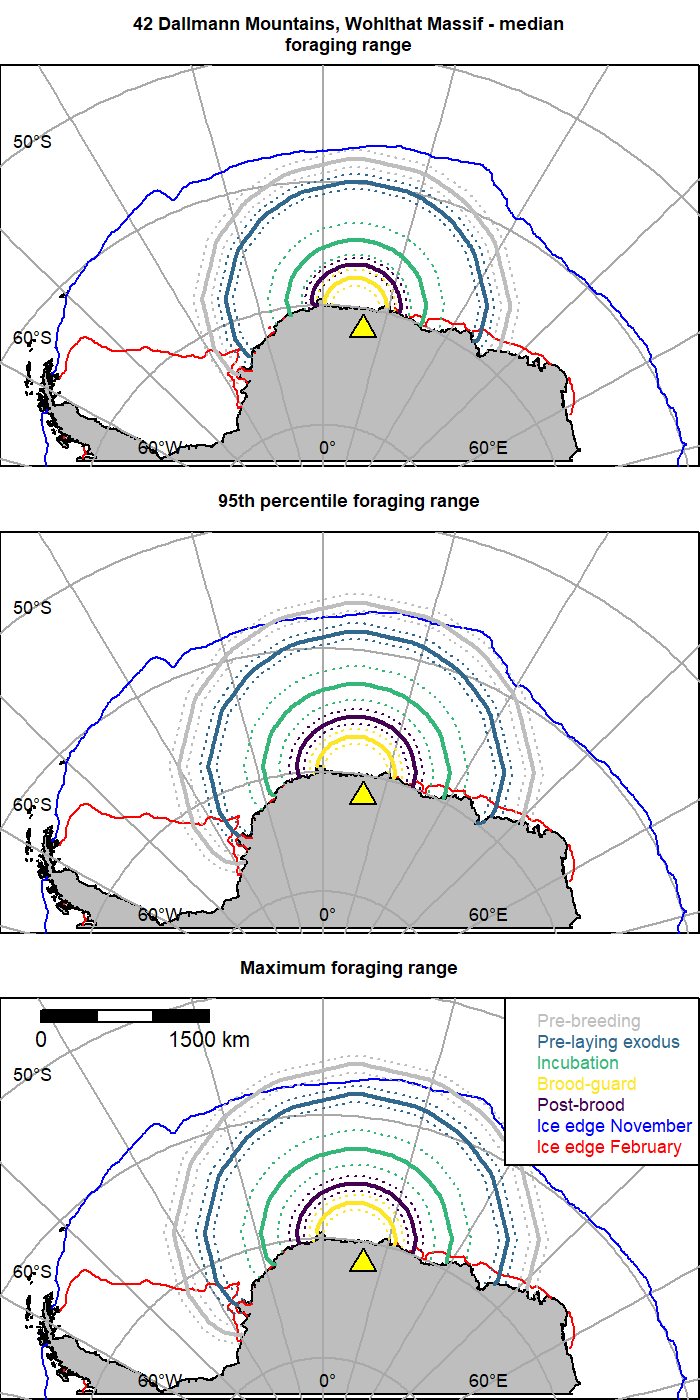

Supplement: Supplementary file 2 — Supplementary material 2 [file 40462_2025_609_MOESM2_ESM.zip › plots/bd_plot_42.png]

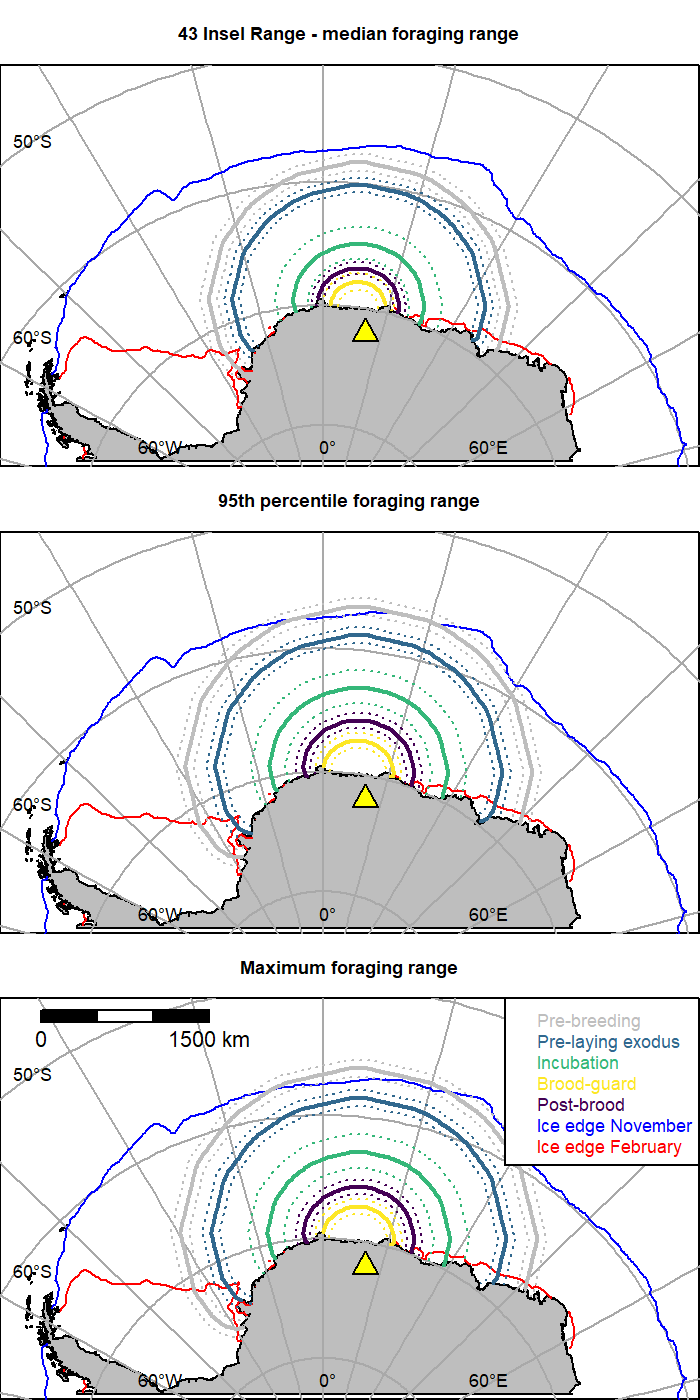

Supplement: Supplementary file 2 — Supplementary material 2 [file 40462_2025_609_MOESM2_ESM.zip › plots/bd_plot_43.png]

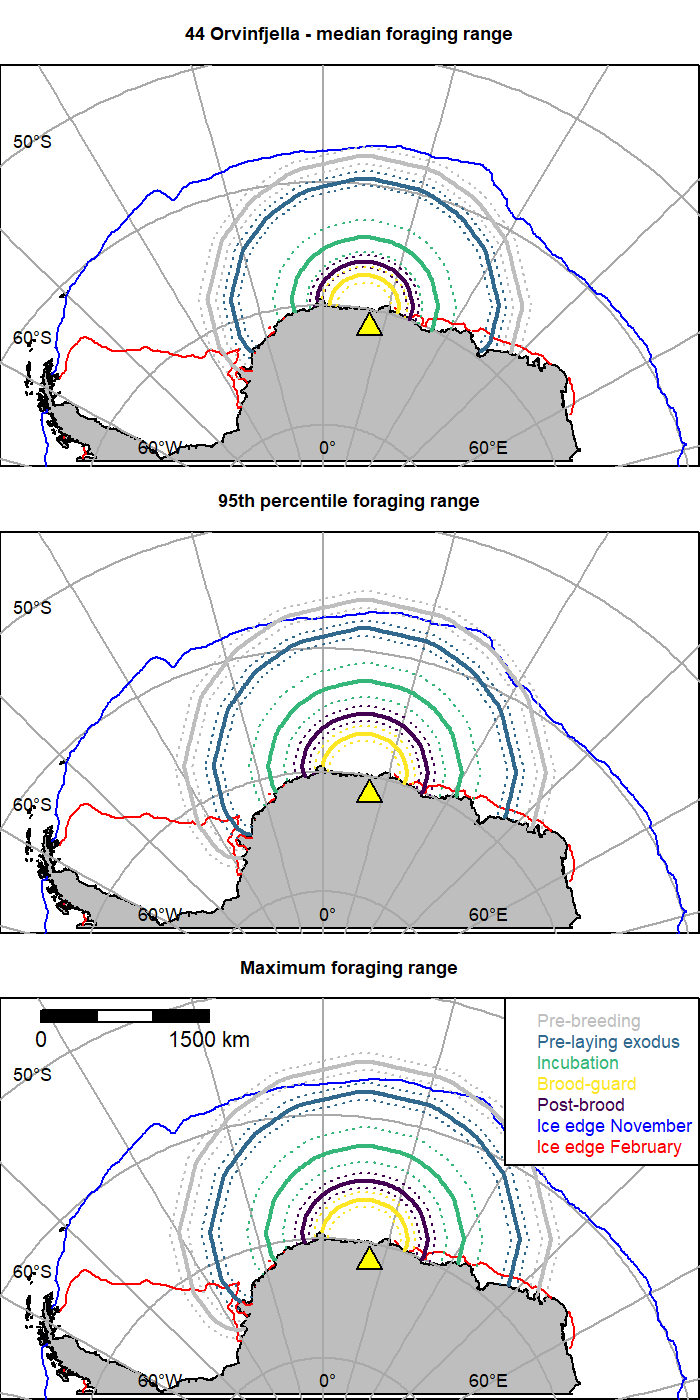

Supplement: Supplementary file 2 — Supplementary material 2 [file 40462_2025_609_MOESM2_ESM.zip › plots/bd_plot_44.png]

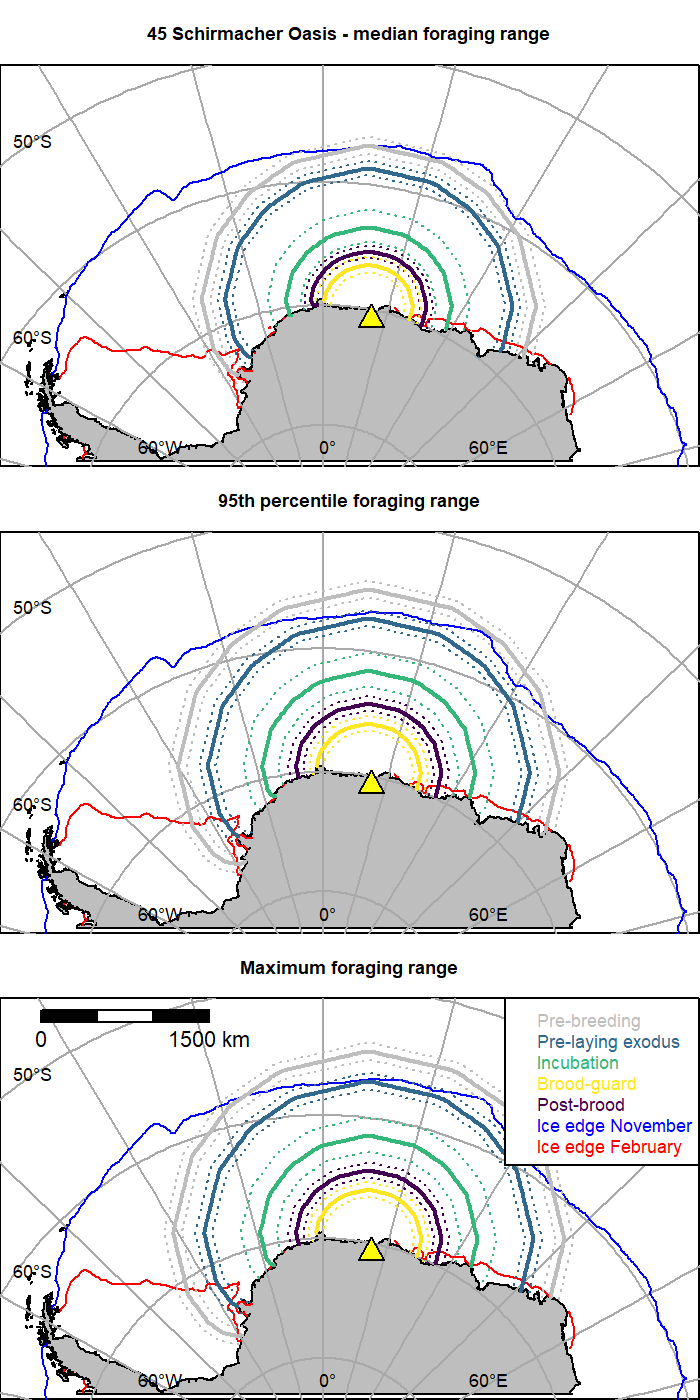

Supplement: Supplementary file 2 — Supplementary material 2 [file 40462_2025_609_MOESM2_ESM.zip › plots/bd_plot_45.png]

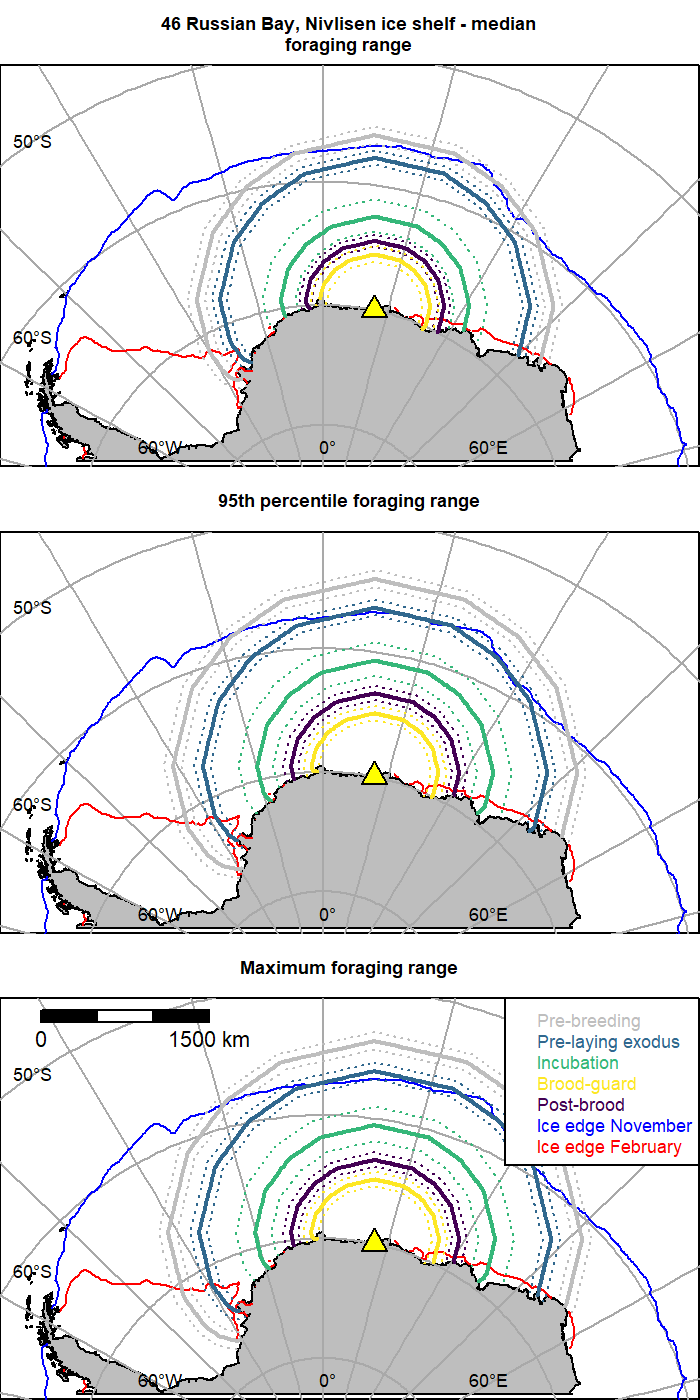

Supplement: Supplementary file 2 — Supplementary material 2 [file 40462_2025_609_MOESM2_ESM.zip › plots/bd_plot_46.png]

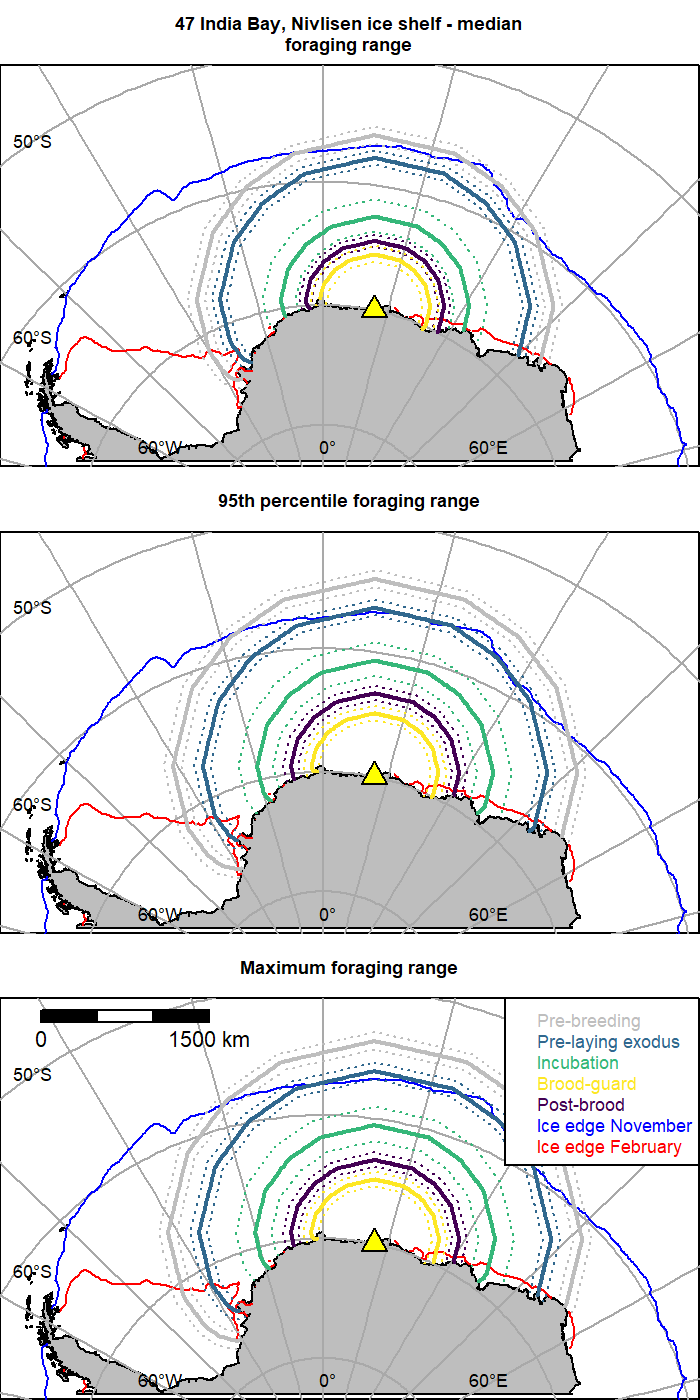

Supplement: Supplementary file 2 — Supplementary material 2 [file 40462_2025_609_MOESM2_ESM.zip › plots/bd_plot_47.png]

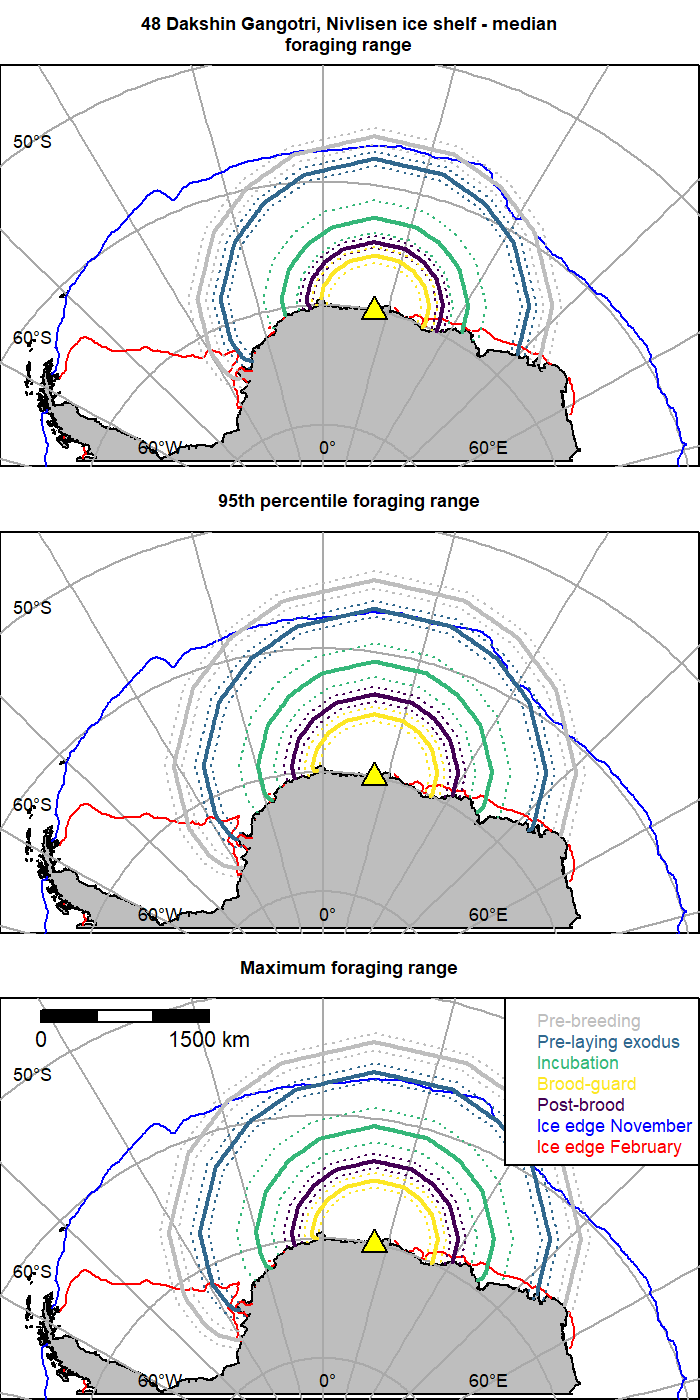

Supplement: Supplementary file 2 — Supplementary material 2 [file 40462_2025_609_MOESM2_ESM.zip › plots/bd_plot_48.png]

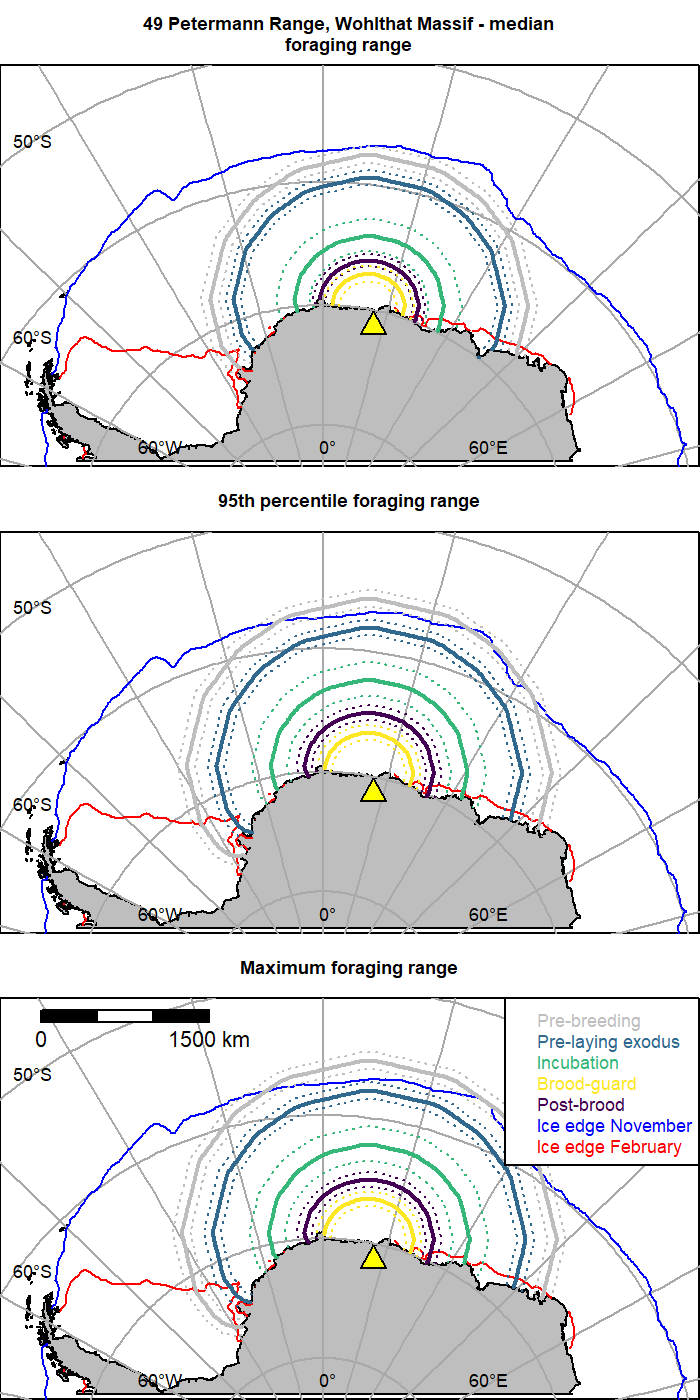

Supplement: Supplementary file 2 — Supplementary material 2 [file 40462_2025_609_MOESM2_ESM.zip › plots/bd_plot_49.png]

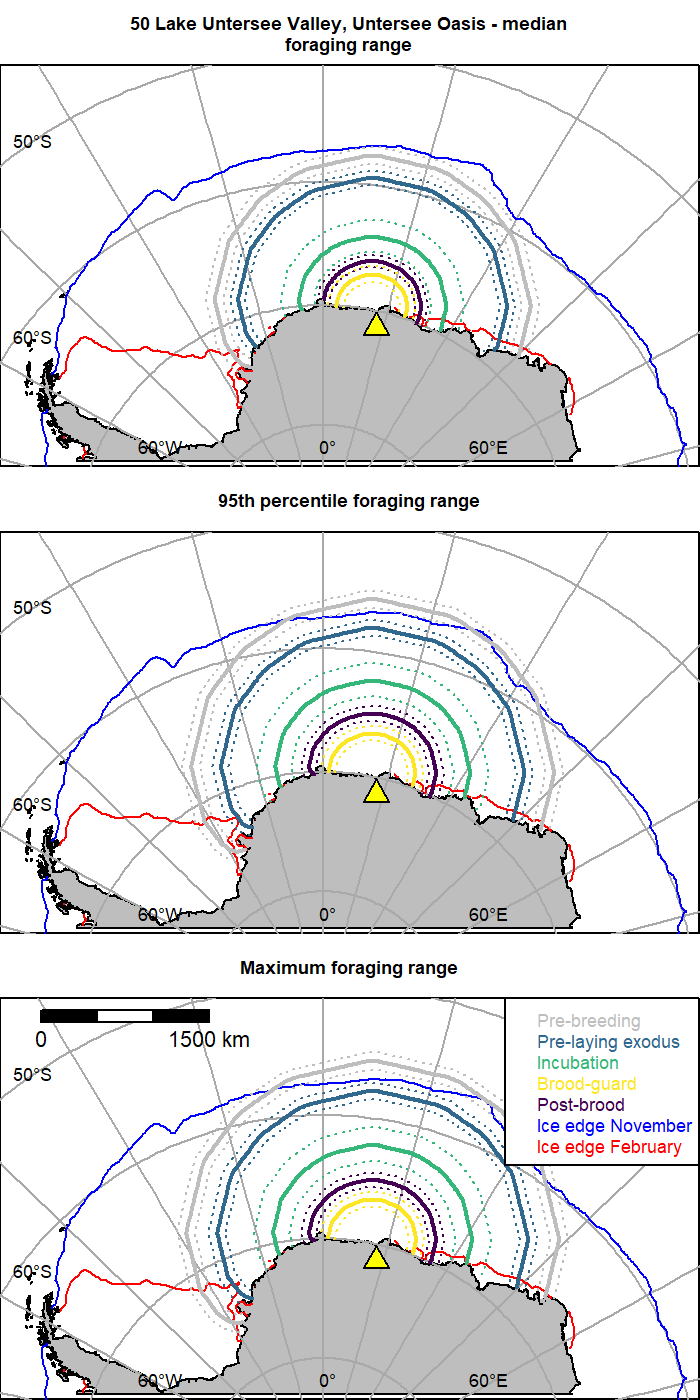

Supplement: Supplementary file 2 — Supplementary material 2 [file 40462_2025_609_MOESM2_ESM.zip › plots/bd_plot_50.png]

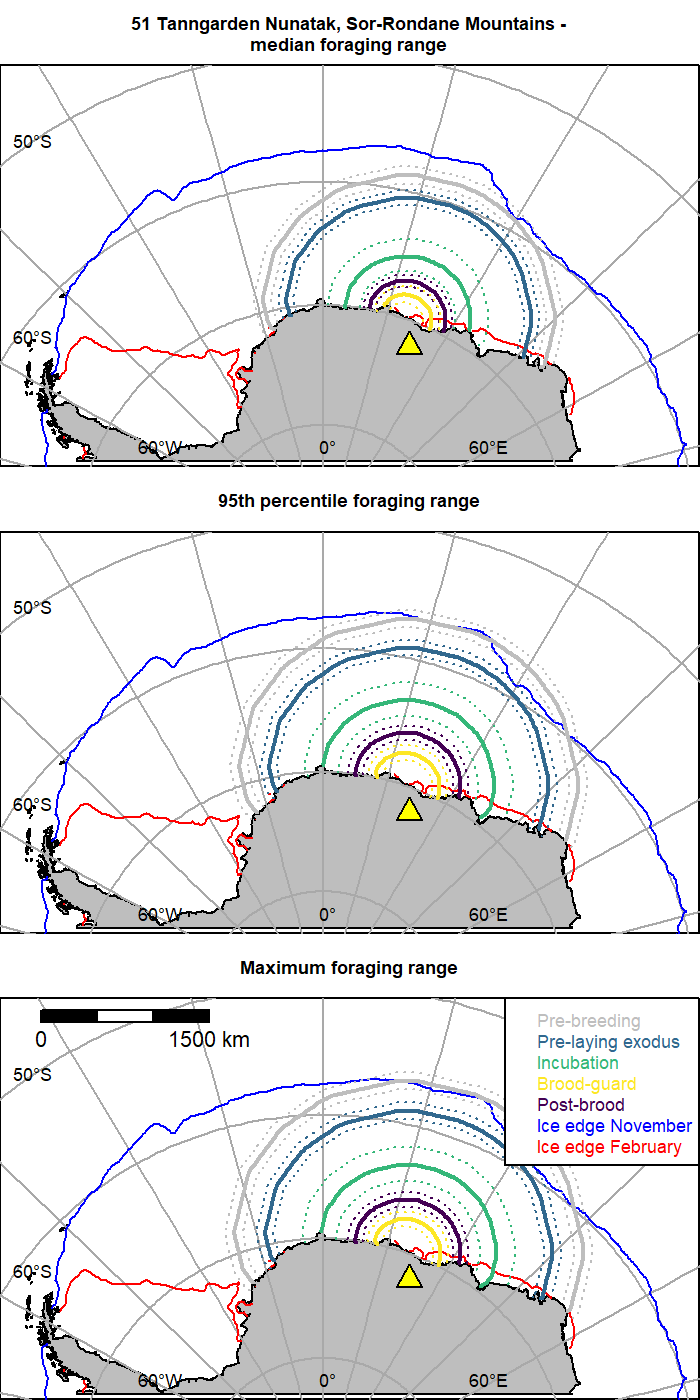

Supplement: Supplementary file 2 — Supplementary material 2 [file 40462_2025_609_MOESM2_ESM.zip › plots/bd_plot_51.png]

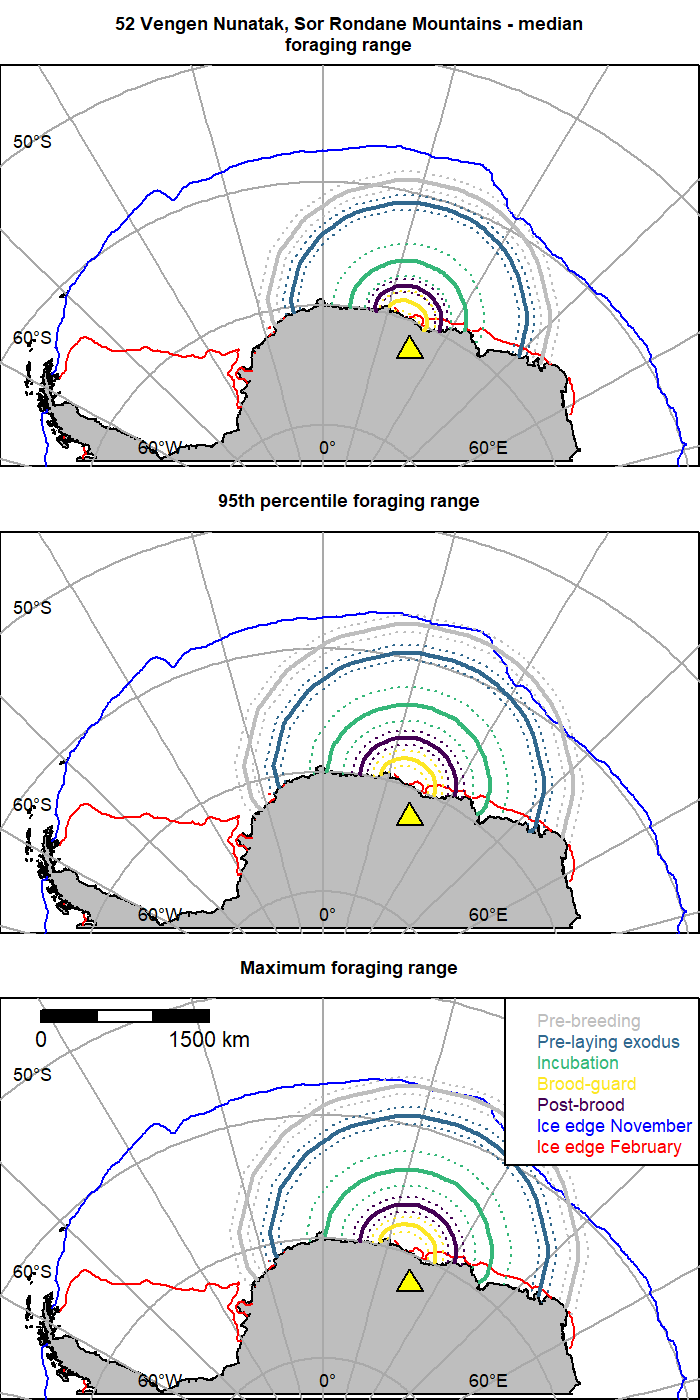

Supplement: Supplementary file 2 — Supplementary material 2 [file 40462_2025_609_MOESM2_ESM.zip › plots/bd_plot_52.png]

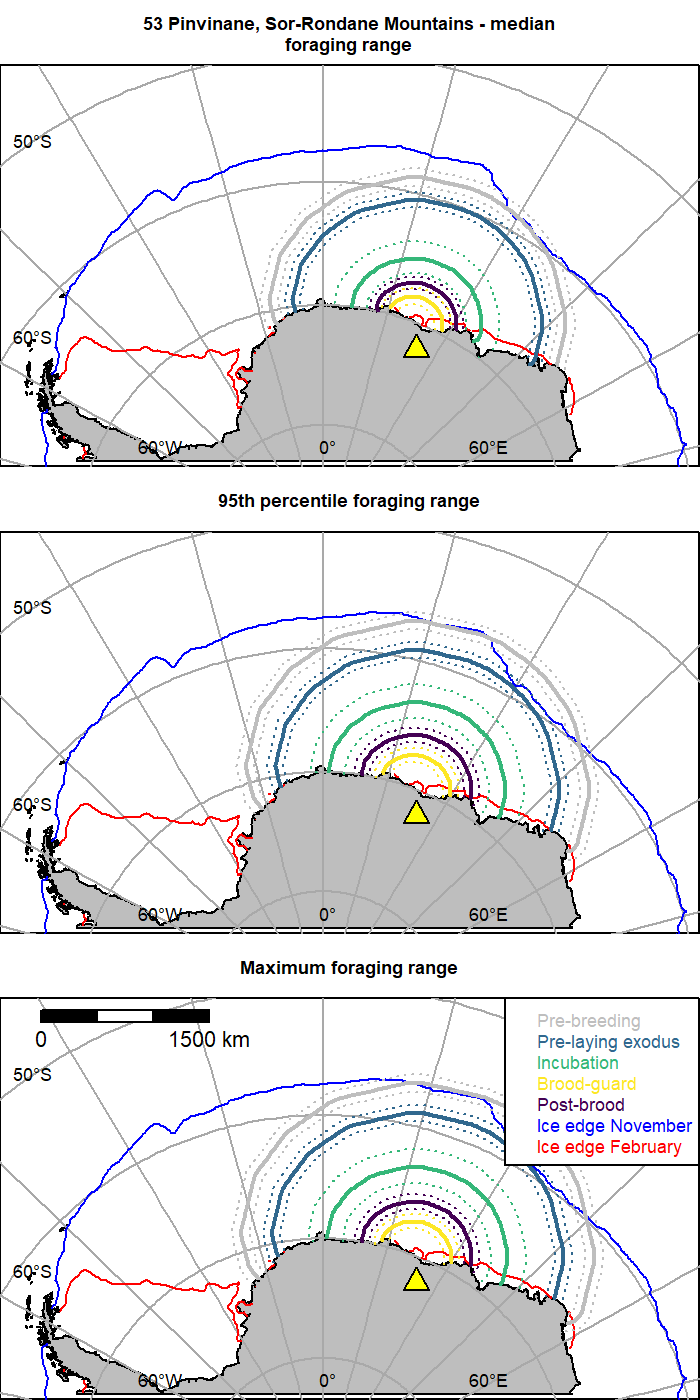

Supplement: Supplementary file 2 — Supplementary material 2 [file 40462_2025_609_MOESM2_ESM.zip › plots/bd_plot_53.png]

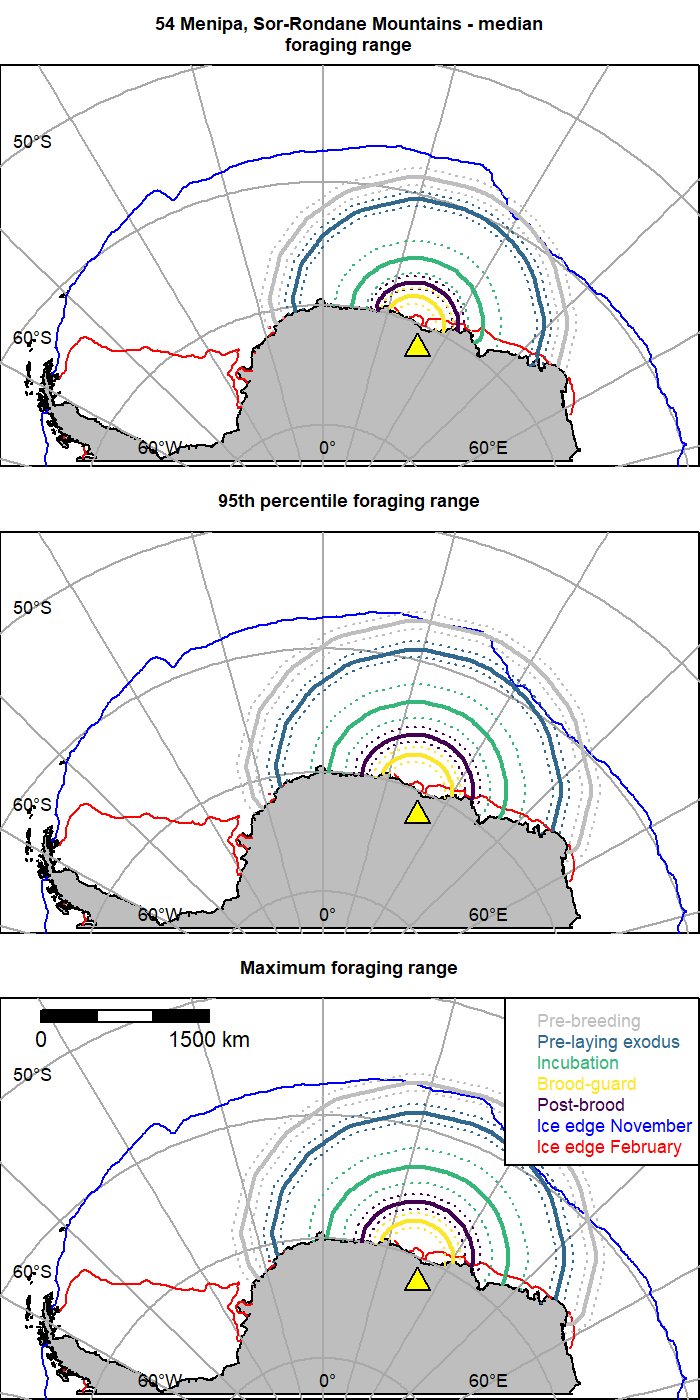

Supplement: Supplementary file 2 — Supplementary material 2 [file 40462_2025_609_MOESM2_ESM.zip › plots/bd_plot_54.png]
